# Possible Role of Gut Microbiota in Polypropylene Microplastics-Induced Immunity and Reproductive Dysfunction in Mice

**DOI:** 10.3390/toxics14080679

**Published:** 2026-07-31

**Authors:** Di Xu, Yunqi Liu, Deli Xu

**Affiliations:** College of Life Sciences, Qufu Normal University, Jingxuan West Street No. 57, Qufu 273165, China

**Keywords:** microplastics, gut microbiota, immunity, reproductive function, testicular gene expression

## Abstract

Polypropylene microplastics (PP-MPs) are ubiquitous in our daily lives, but their toxicological effects on mammals remain poorly understood. This study investigated the toxicity effects of PP-MPs on C57BL/6 mice using 16S rRNA gene amplicon sequencing and transcriptome sequencing. Female and male mice were randomly classified into the control and PP-MPs-treated groups, respectively, and the experiment lasted for 5 weeks. We found that PP-MPs exposure did not affect the levels of immunoglobulin G (IgG), interleukin-4 (IL-4), and interferon gamma (IFN-γ), indicating that humoral immunity and inflammatory levels were not influenced by PP-MPs treatment. However, PP-MPs exposure reduced the PHA response in female mice, but not in male mice. It also did not alter the wet mass of testicles and ovaries, nor the levels of testosterone and estradiol. Exposure to PP-MPs altered the expression of the testicular genes. G protein-coupled receptor signaling pathways, olfactory receptor activity, and protein digestion and absorption were downregulated in the PP group. Collagen genes (*Col9a3*, *Col11a2*, *Col27a1*, *Col26a1*, *Col7a1*) play a significant role in downregulating protein digestion and absorption. In addition, PP-MPs exposure caused a change in beta diversity of gut microbiota, indicating the alteration of their community structure. PP-MPs exposure reduced the relative abundance of the probiotic *Lactobacillus*. Changes in the gut microbiota may be related to the expression levels of testicular genes. Overall, PP-MPs exposure altered both the community structure of the gut microbiota and the expression levels of testicular genes in mice, and collagen genes may serve as a critical factor influencing testicular function.

## 1. Introduction

Microplastics are plastic fragments with a diameter of less than 5 mm, which are categorized into two distinct types: primary microplastics and secondary microplastics [1,2,3]. They pose very serious threats to physiological processes such as immune and reproductive function in terrestrial mammals [4,5].

The reproductive system is sensitive to environmental toxins [6]. Exposure to polystyrene microplastics (PS-MPs) was found to reduce sperm quality and serum testosterone levels in mice, while increasing the rate of sperm deformity and damaging spermatocytes [7,8]. Exposure to polytetrafluoroethylene (PTFE) significantly reduced the sperm number and concentration, disrupting the synthesis and secretion of testicular steroid hormones. [9]. Polylactic acid (PLA) microplastics caused seminiferous tubule degeneration, germ cell shedding, and testicular structural disruption [10]. Specifically, male reproductive dysfunction is associated with microplastic accumulation in the testes and alterations in testicular gene expression [11,12,13,14]. However, some studies have also shown that microplastics can alter testicular morphology but do not affect the secretion of sex hormones [15] and sperm physiological function [16].

The effects of microplastics on the immune system mainly involve immunosuppression, immune activation, and inflammatory responses [17,18]. For instance, PS-MPs can induce spleen damage, inhibit the phagocytic activity of macrophages, and destroy innate immune function [19,20]. Exposure to polyethylene (PE) microplastics has been shown to reduce the quantities of B cells and T helper cells in the spleens of mice [21]. Microplastics have been found to increase plasma IgG1 levels [22]. PP-MPs enhanced the hypersensitivity of mice by stimulating the immune system [22]. Inflammation is a biological response to harmful stimuli [23]. The accumulation of microplastics within immune cells induced the release of pro-inflammatory cytokines either directly or indirectly, thereby triggering inflammatory responses [18,23]. Research indicated that PP-MPs are associated with elevated levels of pro-inflammatory factors, thereby inducing intestinal inflammation [18]. Exposure to PS-MPs increased the IL-4 levels and decreased tumor necrosis factor levels [17].

Exposure to microplastics also can impair the intestinal barrier, increase intestinal inflammation, and alter the community structure of gut microbiota [24]. Specifically, exposure to polyvinyl chloride (PVC) microplastics has been shown to increase the relative abundance of Proteobacteria in rabbits [25]. Additionally, exposure to PS-MPs significantly reduced the relative abundance of Firmicutes, while increasing the relative abundance of Proteobacteria and Actinobacteria [26]. The gut microbiome is considered an additional “organ” that plays a crucial role in various physiological activities of the host, including immunity and reproduction [27]. For instance, gut microbiota can stimulate immune cells to secrete cytokines and inflammatory factors, thus regulating the development and function of immune cells [28]. Furthermore, the alpha diversity of gut microbiota exhibits a positive correlation with testosterone levels [29]. Gut microbiota also influences sperm production through the regulation of testosterone [30].

It is reported that PP is the most widely used in China and is also the most common polymer in freshwater resources [31]. They are also widely used in take-out food containers and infant feeding bottles [32,33]. However, the mechanisms through which PP-MPs affect immunity and reproductive function remain unclear, and we speculated that the gut microbiota may mediate the effect of PP-MPs on immune and reproductive processes. Therefore, this study employed microbiome and transcriptome sequencing to analyze the impacts of PP-MPs on the immune system and male reproductive function, from the perspective of the gut microbiota. This study will provide new insights into the potential health risks caused by microplastics, thereby offering critical scientific support for addressing the global challenge of microplastic pollution.

## 2. Materials and Methods

### 2.1. Ethics Statement

This study was approved by the Bioethics Committee of Qufu Normal University (protocol code: 2021013, date: 2 March 2023), and adhered to Chinese laws and the requirements of the China Animal Protection Association.

### 2.2. Experimental Design

Adult C57BL/6 mice were individually housed in plastic cages (30 cm × 15 cm × 20 cm) under controlled environmental conditions [34]. The temperature was maintained at 22 ± 1 °C, and the photoperiod was set at 12 h light: 12 h dark. The mice were randomly divided into the following four groups using a stratified randomization method: female control (CONF, *n* = 5), female PP (PPF, *n* = 6), male control (CONM, *n* = 8), male PP (PPM, *n* = 8). PP-MPs with a particle size of 5 μm were purchased from Run Hong Plastic Company (Shanghai, China). The microplastic powder was dissolved in sterile water in order to prepare an aqueous suspension with a concentration of 20 mg/mL. The PP groups received 0.2 mL of PP-MPs aqueous suspension, while the control groups received an equal volume of sterile deionized water, via oral gavage once daily. The experiment lasted for 5 weeks. Body mass was measured once a day, and the food intake was measured every week. The food intake was the average over a period of three days. At the end of the experiment (i.e., after 5-week treatment), all mice were euthanized with carbon dioxide, and trunk blood was collected. To minimize the influence of variation in operational timing, we employed a blocking strategy for sample collection. The organs (testis, cecum, spleen, ovary, uterus, thymus, seminal vesicle) were dissected, weighed, and stored at −80 °C until DNA and RNA were extracted. Serum was separated from venous blood for subsequent analysis.

### 2.3. Cellular Immunity Assay

The cellular immunity was detected by injecting exogenous immune-stimulating substances (phytohemagglutinin, PHA) [34,35]. Three days before they were killed, the footpad thickness of the left hind foot of mice was measured using a micrometer (547-301 Absolute Digimatic Indicator ID-C, Mitutoyo Co., Kanagawa, Japan) and recorded as the initial footpad thickness. Subsequently, 0.1 mg PHA solution was injected into the left footpad. The thickness of the footpad was measured at 6 h, 12 h, 24 h, and 48 h post-injection. Six measurements were taken at each time point, and their averages represent the footpad thickness. The cellular immunity level of mice was evaluated by (post-injection footpad thickness-initial footpad thickness)/initial footpad thickness.

### 2.4. Humoral Immunity Assay

To evaluate humoral immunity (anti-KLH IgG), mice in each group were subcutaneously injected with keyhole limpet hemocyanin (KLH, Sigma LH7017, FeimoBio Co. Ltd., Beijing, China) 10 days before they were killed. The trunk blood samples were centrifuged at 3500 rpm for 10 min at 4 °C to isolate the serum. Anti-KLH IgG was detected by Enzyme-linked immunosorbent assay (ELISA) according to our previous methods [36]. The detailed procedures can be found in the supporting materials (Supplementary Information S1).

### 2.5. Assay of Cytokines and Sex Hormone

The concentrations of IL-4 (ZY65009-A), IFN-γ (ZY65007-A), testosterone (ZY6015-A), and estradiol (ZY6009-A) in serum were detected using their respective ELISA Kits. All the kits were purchased from Shanghai Jiupin Biotechnology Co., Ltd., Shanghai, China. All testing procedures were carried out in accordance with the instructions provided by the manufacturer.

### 2.6. Transcriptome Sequencing

In our study, the TRIzol method was employed to extract total RNA from the testis, and the total amount and integrity of RNA were detected by the Bioanalyzer 2100 system (Agilent Technologies, Santa Clara, CA, USA). Then, mRNA with polyA tails was enriched by Oligo (dT) magnetic beads for library construction. The library was checked with Qubit2.0 Fluorometer (Thermo Fisher Scientific Inc., Waltham, MA, USA) and real-time PCR for quantification. After library quality control, different libraries were pooled based on the effective concentration and targeted data amount, then subjected to Illumina sequencing.

The clean reads were obtained by removing reads containing adapters, reads containing ploy-N, and low-quality reads from raw data. Concurrently, Q20, Q30, and GC content of the clean data were calculated. The clean reads were aligned to the reference genome using HISAT2 (Version: 2.0.5) software to obtain the positional information of the reads on the reference genome. The gene expression levels were quantitatively analyzed using the featureCounts tool in subread (Version: 1.5.0-p3) software. Due to the influence of sequencing depth and gene length, the gene expression level of RNA-seq is generally represented by FPKM. Therefore, the FPKM values of all genes in each sample were calculated. The correlation coefficients between samples were calculated based on the FPKM values of all genes in each sample. PCA was conducted to evaluate the differences between groups, as well as the replication of samples within groups. After the quantitative expression of genes, the DESeq2 (Version: 1.20.0) software was used to conduct differential expression analysis of genes (|log_2_ (FoldChange)| ≥ 1, *p*-adj ≤ 0.05). To analyze the functions of differentially expressed genes (DEGs), clusterProfiler (Version: 3.8.1) software was used to conduct Gene Ontology (GO) and Kyoto Encyclopedia of Genes and Genomes (KEGG) enrichment analysis on the differentially expressed gene set. To identify biologically important pathways without relying on the significance thresholds for individual genes, Gene Set Enrichment Analysis (GSEA) was conducted.

### 2.7. 16s rRNA Amplicon Sequencing

The DNA was extracted from the cecal contents using the cetyltrimethylammonium bromide (CTAB) method. After the DNA sample quality control was passed, the V3-V4 regions of the 16S rRNA genes were amplified using the specific primers (515F and 806R) with the barcode. After the PCR products were purified and mixed, they were detected, and target bands were recovered. Then, sequencing libraries were generated. The library was checked with Qubit and Q-PCR for quantification. After library quality control was passed, double-terminal sequencing was performed on the Illumina NovaSeq sequencing platforms.

To analyze the richness and diversity of the gut microbiota in each sample, the alpha diversity indices (observed otus, shannon, simpson, chao1, goods coverage, dominance and pielou_e) were calculated using the QIIME2 software. The differences in alpha diversity indices among groups were compared using the Wilcoxon rank-sum test and Kruskal–Wallis test. In order to evaluate the complexity of the community composition and compare the differences among groups, beta diversity was calculated based on weighted and unweighted Unifrac distances in QIIME2. Analyses including principal component analysis (PCA), principal co-ordinates analysis (PCoA), non-metric multi-dimensional scaling (NMDS), and unweighted pair-group method with arithmetic mean (UPGMA) were also performed. Additionally, the Adonis and Anosim analyses were employed to assess whether significant differences existed in the community structure among the different groups. The species with significant differences among different groups were analyzed by *t*-test and MetagenomeSeq analysis. The LEfSe analysis was employed to identify biomarkers that exhibited statistically significant differences among the groups.

### 2.8. Correlation Analysis

To analyze the role of the gut microbiota in male reproductive function, correlation analysis was performed between DEGs in the testis and gut microbiota relative abundance. These included Pearson correlation analysis, canonical correlation analysis (CCA), and orthogonalized partial least squares analysis (O2PLS). Differences were considered statistically significant at *p* < 0.05.

### 2.9. Statistical Analysis

Prior to all statistical analyses, data were examined for normality and homogeneity of variance, using Kolmogorov–Smirnov and Levene tests, respectively. All physiological index data tests were performed using IBM SPSS Statistics (version 27.0.1, IBM Inc., Armonk, NY, USA). Body mass was analyzed using a linear mixed model (LMM). Repeated measures ANOVA was used for the analysis of food intake and PHA responses. In addition, one-way analysis was used to analyze the PHA responses in females and males, respectively. The differences between groups of each index (spleen mass, thymus mass, IgG, IL-4, IFN-γ) were evaluated by two-way (gender × microplastics) ANOVA analysis. Differences in reproductive-related indicators (uterus mass, ovary mass, testis mass, and seminal vesicle mass) between groups were compared using a general linear model (GLM) multivariate analysis. Intergroup differences in estradiol and testosterone were analyzed using independent-samples *t*-tests. Analysis of organ mass differences was performed using final body mass as a covariate to control for body mass variation. Food intake was divided by body mass^0.67^ to eliminate the influence of body mass. All results were visualized using Graphpad Prism (version 9.5).

## 3. Results

### 3.1. Body Mass and Food Intake

**Figure 1:**
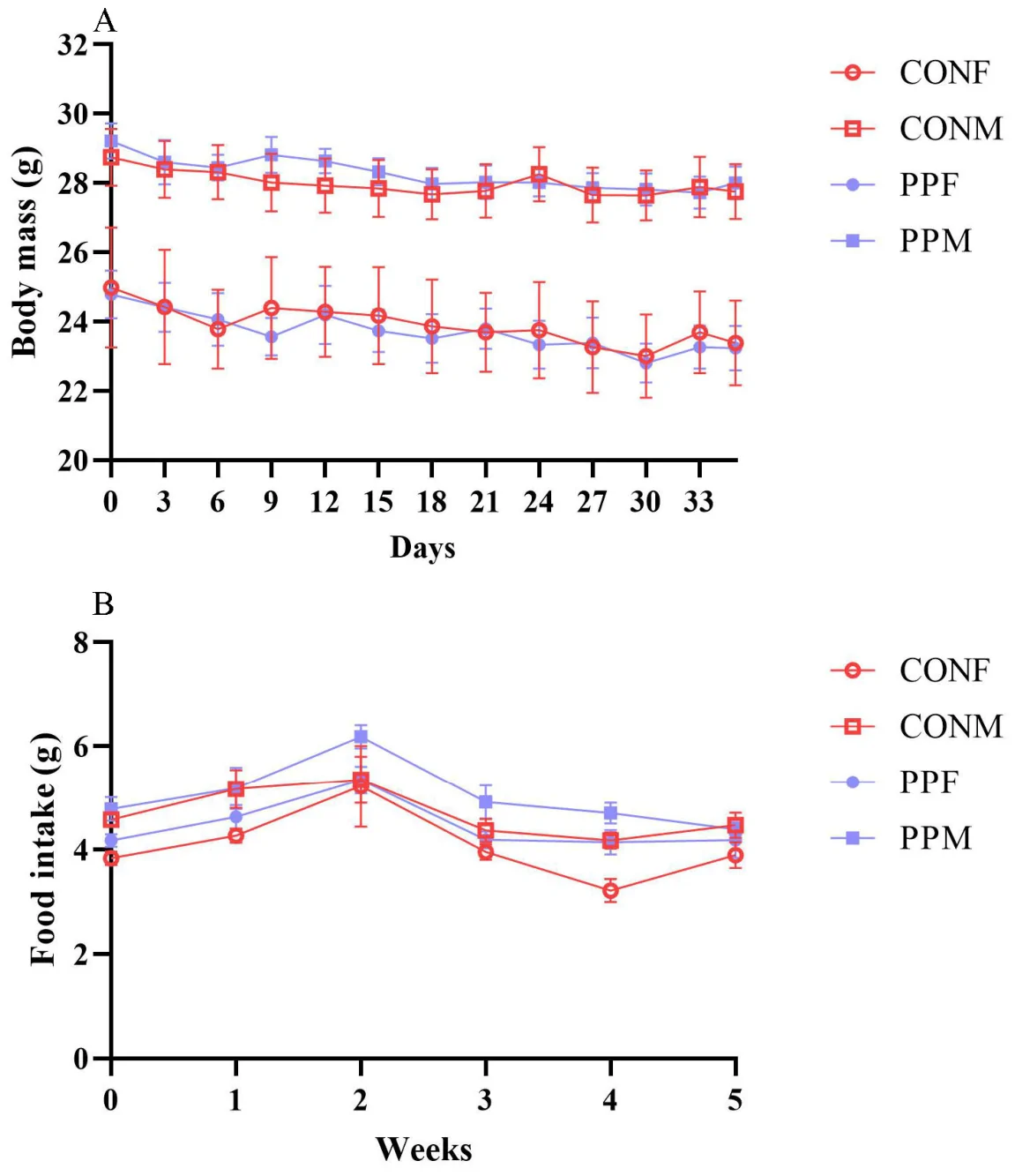
Changes in body mass (**A**) and food intake (**B**) after exposure to PP-MPs.

Body mass differed significantly between males and females (*F*_(1, 26.999)_ = 39.269, *p* < 0.001), but did not differ between the PP groups and the control groups (*F*_(1, 26.999)_ = 0.179, *p* = 0.676) (Figure 1A). The interaction of sex and PP-MPs had no effect on body mass (*F*_(1, 26.999)_ = 0.003, *p* = 0.956). Additionally, body mass was affected by time (*F*_(35, 943.999)_ = 8.313, *p* < 0.001), but not by the interaction of time and sex (*F*_(35, 943.999)_ = 1.037, *p* = 0.411) or the interaction of time and PP-MPs (*F*_(35, 943.999)_ = 0.758, *p* = 0.845).

Food intake was affected by PP-MPs (*F*_(1, 22)_ = 7.329, *p* = 0.013, η^2^_p_ = 0.250), not by sex (*F*_(1, 22)_ = 0.334, *p* = 0.569, η^2^_p_ = 0.015), and the interaction of sex and PP-MPs (*F*_(1, 22)_ = 0.437, *p* = 0.515, η^2^_p_ = 0.019). Additionally, food intake was affected by time (*F* _(2.327, 51.203)_ = 21.220, *p* < 0.001, η^2^_p_ = 0.491), but not by the interaction of time and sex (*F*_(2.327, 51.203)_ = 0.305, *p* = 0.771, η^2^_p_ = 0.014) or the interaction of time and PP-MPs (*F*_(2.327, 51.203)_ = 0.899, *p* = 0.426, η^2^_p_ = 0.039).

### 3.2. Cellular Immunity

**Figure 2:**
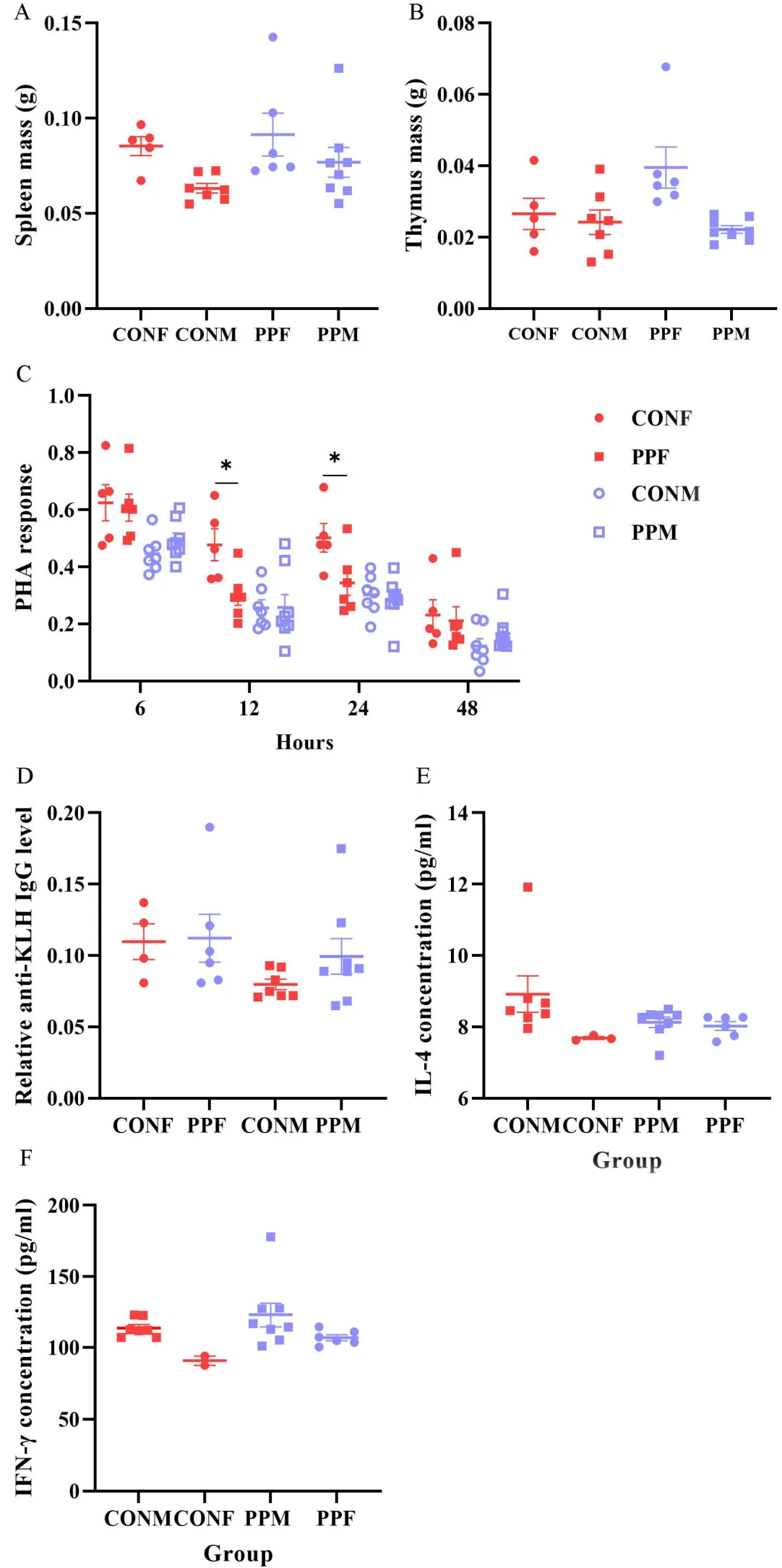
Following exposure to PP-MPs, changes in wet thymus mass (**A**), wet spleen mass (**B**), anti-KLH IgG (**D**), IL-4 (**E**), and IFN-γ (**F**). PHA reaction at 6 h,12 h, 24 h, and 48 h after PHA injection (**C**). The asterisk (*) indicates *p* < 0.05.

In this study, cellular immunity was evaluated by phytohemagglutinin (PHA) response. PHA response was significantly affected by sex (*F*_(1, 22)_ = 13.065, *p* = 0.002, η^2^_p_ = 0.373), but not by PP-MPs (*F*_(1, 22)_ = 1.230, *p* = 0.279, η^2^_p_ = 0.053), nor by the interaction of sex and PP-MPs (*F*_(1, 22)_ = 2.810, *p* = 0.108, η^2^_p_ = 0.113). Female PHA response was higher than that of males. Exposure to PP-MPs significantly reduced the PHA response of female mice at 12 h (*F*_(1, 9)_ = 7.751, *p* = 0.021, η^2^_p_ = 0.463) and 24 h (*F*_(1, 9)_ = 5.749, *p* = 0.040, η^2^_p_ = 0.390), while the PHA response of male mice at 6 h, 12 h, 24 h, and 48 h was not significantly affected by PP-MPs (Figure 2C). PHA response was significantly affected by time (*F*_(3, 66)_ = 198.531, *p* < 0.001, η^2^_p_ = 0.9). The highest PHA response was observed at 6 h after injection.

### 3.3. Humoral Immunity

Serum anti-KLH IgG concentration was not affected by sex (*F*_(1, 21)_ = 2.882, *p* = 0.104, η^2^_p_ = 0.121), PP-MPs treatment (*F*_(1, 21)_ = 0.766, *p* = 0.391, η^2^_p_ = 0.035), or their interaction of sex and PP-MPs treatment (*F*_(1, 21)_ = 0.467, *p* = 0.502, η^2^_p_ = 0.022) (Figure 2D).

### 3.4. Immune Organs and Cytokines

Wet thymus mass was not affected by sex (*F*_(1, 21)_ = 3.877, *p* = 0.062, η^2^_p_ = 0.156), PP-MPs (*F*_(1, 21)_ = 2.031, *p* = 0.169, η^2^_p_ = 0.088), and the interaction between PP-MPs and sex (*F*_(1, 21)_ = 3.908, *p* = 0.061, η^2^_p_ = 0.157) (Figure 2B). Wet spleen mass was affected by sex (*F*_(1, 21)_ = 7.468, *p* = 0.012, η^2^_p_ = 0.262), but not by PP-MPs (*F*_(1, 21)_ = 1.637, *p* = 0.215, η^2^_p_ = 0.072), and the interaction between PP-MPs and sex (*F*_(1, 21)_ = 0.303, *p* = 0.588, η^2^_p_ = 0.014) (Figure 2A). The spleen mass in females was higher than in males. A significant difference was observed in serum IFN-γ levels between males and females (*F*_(1, 19)_ = 6.841, *p* = 0.017, η^2^_p_ = 0.265) (Figure 2F). IL-4 level was not affected by PP-MPs (*F*_(1, 20)_ = 0.422, *p* = 0.523, η^2^_p_ = 0.021), sex (*F*_(1, 20)_ = 3.687, *p* = 0.069, η^2^_p_ = 0.156), or the interaction between sex and PP-MPs (*F*_(1, 20)_ = 2.617, *p* = 0.121, η^2^_p_ = 0.116) (Figure 2E).

### 3.5. Sex Organs and Sex Hormone

**Figure 3:**
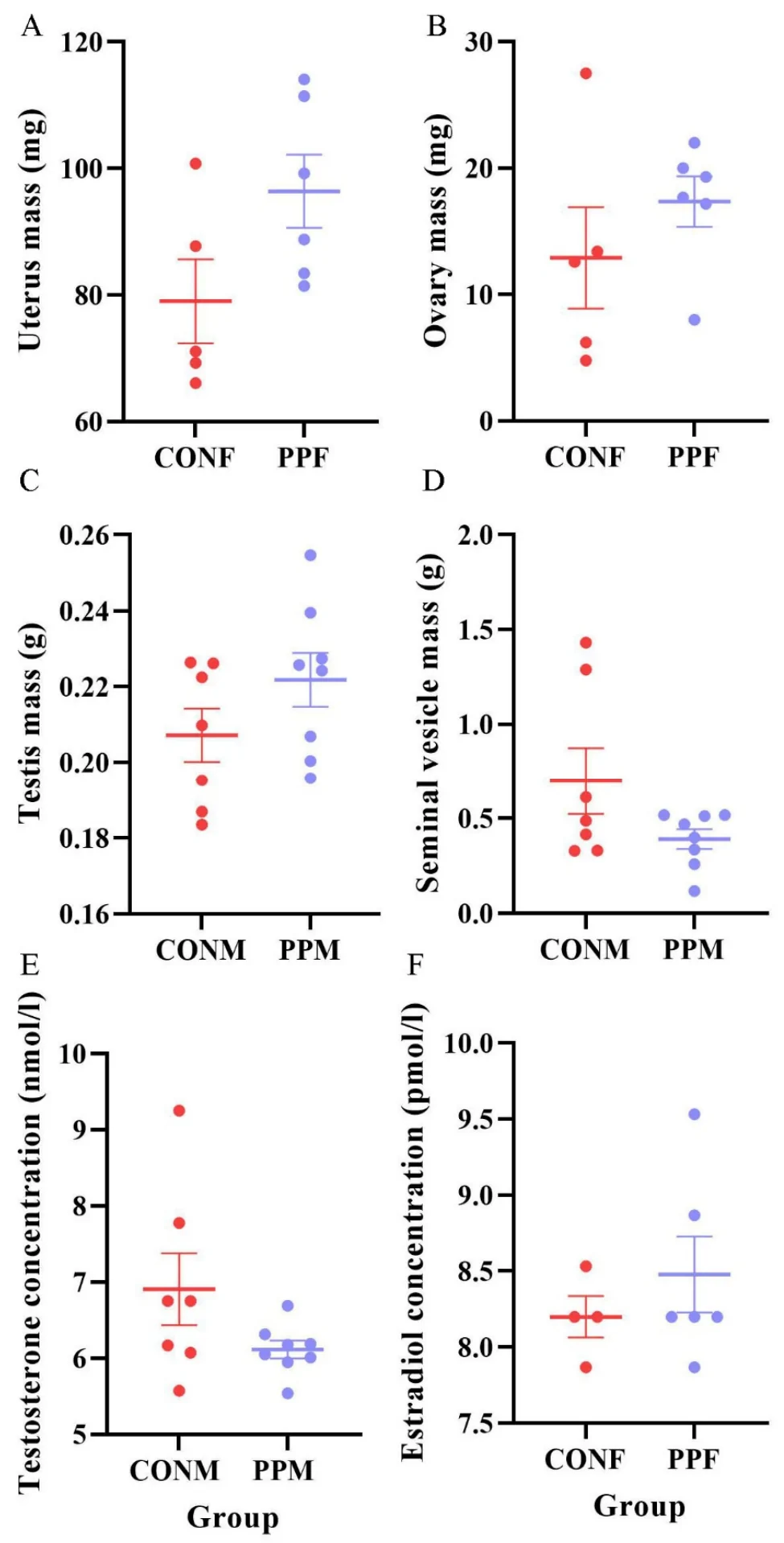
Effects of exposure to PP-MPs on the uterus (**A**), ovary (**B**), testis (**C**), seminal vesicle (**D**), testosterone (**E**), and estradiol (**F**).

The wet mass of the uterus (*F*_(1, 7)_ = 3.650, *p* = 0.098, η^2^_p_ = 0.343), ovary (*F*_(1, 7)_ = 0.185, *p* = 0.680, η^2^_p_ = 0.026), testis (*F*_(1, 11)_ = 0.076, *p* = 0.788, η^2^_p_ = 0.007), and seminal vesicle (*F*_(1, 11)_ = 1.066, *p* = 0.324, η^2^_p_ = 0.088), as well as the concentrations of serum estradiol (*t* = −0.841, *df* = 8, *p* = 0.425, η^2^ = 0.511) and testosterone (*t* = −1.631, *df* = 6.727, *p* = 0.149, η^2^ = 0.881), were not significantly affected by PP-MPs (Figure 3).

### 3.6. Transcriptome Analysis of Testis

**Figure 4:**
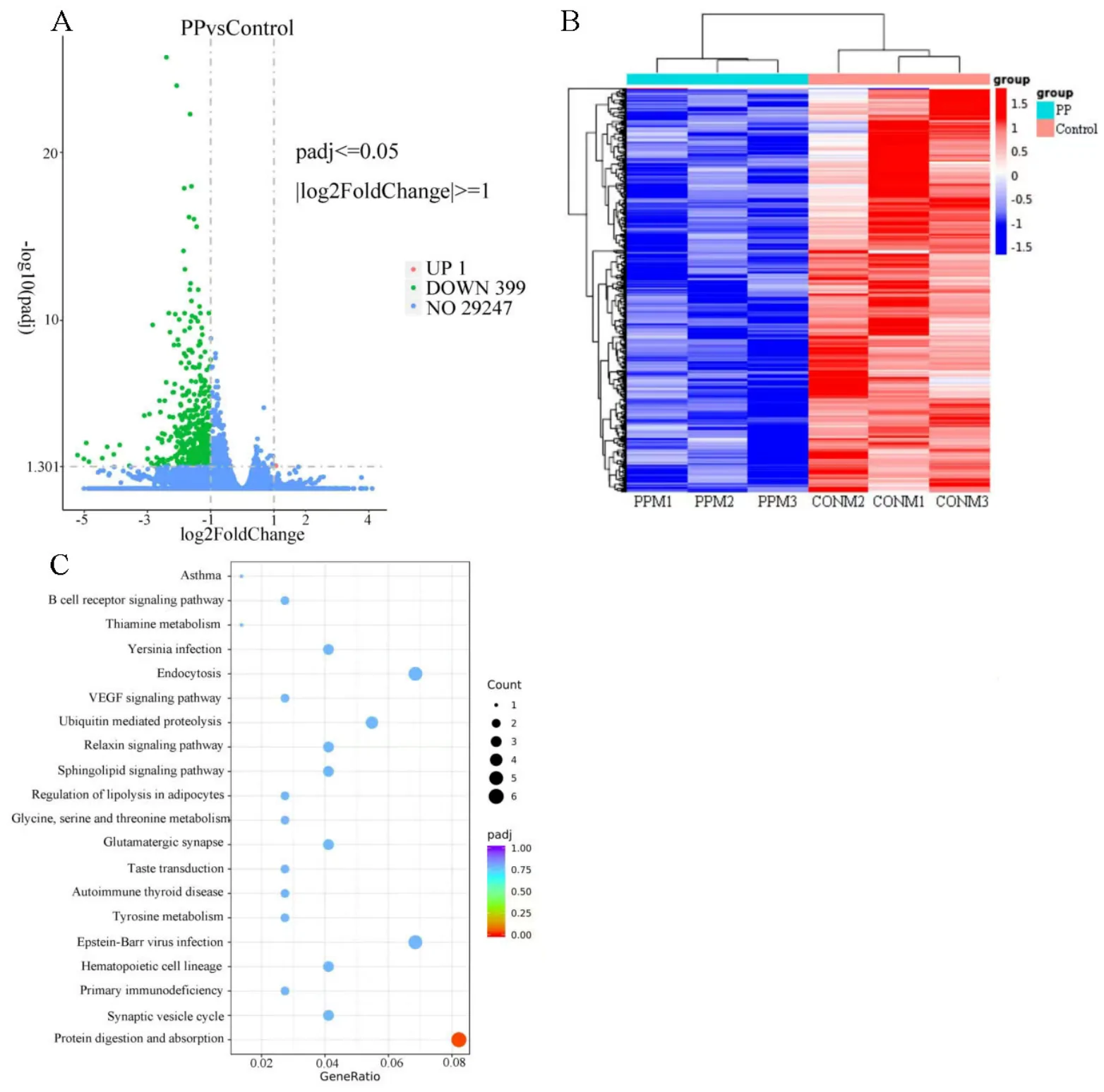
Distribution (**A**) and clustering (**B**) of differential genes between the PP group and the control group. Scatter plot of KEGG enrichment results (**C**).

The testis transcriptome was used to assess reproductive function in male mice. The results of the cluster analysis revealed that the gene expression patterns were similar within each group (Figure 4B). There were 400 differentially expressed genes (DEGs) in the control and PP groups, and 1 DEG was upregulated, and 399 DEGs were downregulated in the PP group (Figure 4A).

Based on the GO annotation results, DEGs between the control and PP groups were enriched in protein phosphorylation, regulation of hydrolase activity, phosphorylation, extracellular region part, extracellular region, ion channel complex, protein kinase activity, DNA polymerase activity, sodium ion transmembrane transporter activity, transmembrane transporter activity, and other terms (Supplementary Information S2). KEGG pathway enrichment analysis revealed that the downregulated DEGs were enriched in 196 pathways, including protein digestion and absorption, B cell receptor signaling pathway, ubiquitin-mediated proteolysis, regulation of lipolysis in adipocytes, hematopoietic cell lineage, and other pathways. Only the protein digestion and absorption pathway was significantly downregulated in the PP group, which was related to the downregulation of collagen genes (*Col9a3*, *Col11a2*, *Col27a1*, *Col26a1*, *Col7a1*) (Figure 4C). The upregulated DEGs were significantly enriched in RNA degradation and spliceosome pathways.

Gene Set Enrichment Analysis (GSEA) results revealed 437 gene sets within the GO gene set were found to be upregulated in the PP group, such as cell redox homeostasis, protein localization to organelle, regulation of transcription by RNA polymerase II, mitotic cell cycle, and whole membrane. There were 242 gene sets upregulated in the control group, including regulation of hydrolase activity, pheromone receptor activity, regulation of programmed cell death, regulation of cell death, regulation of apoptotic process, G protein coupled receptor signaling pathway, olfactory receptor activity, and others. In the KEGG dataset, 160 gene sets were upregulated in the PP group, including aminoacyl tRNA biosynthesis, citrate cycle (TCA cycle), selenocompound metabolism, glycosphingolipid biosynthesis ganglio series, and so on. In the control group, 173 pathways were found to be upregulated, such as lipoic acid metabolism, protein digestion and absorption, primary immunodeficiency, tyrosine metabolism (Supplementary Information S3–S6).

### 3.7. Diversity of Gut Microbiota

In this study, a total of 2,679,128 raw tags were obtained. After splicing and filtering out low- quality and short- length sequences, 2,590,811 clean tags were obtained. A total of 1,611,934 effective tags were obtained for subsequent analysis. The results of the sample rarefaction curves indicate that the amount of sequencing data and the sequencing depth were reasonable (Supplementary Information S7 and S8).

**Figure 5:**
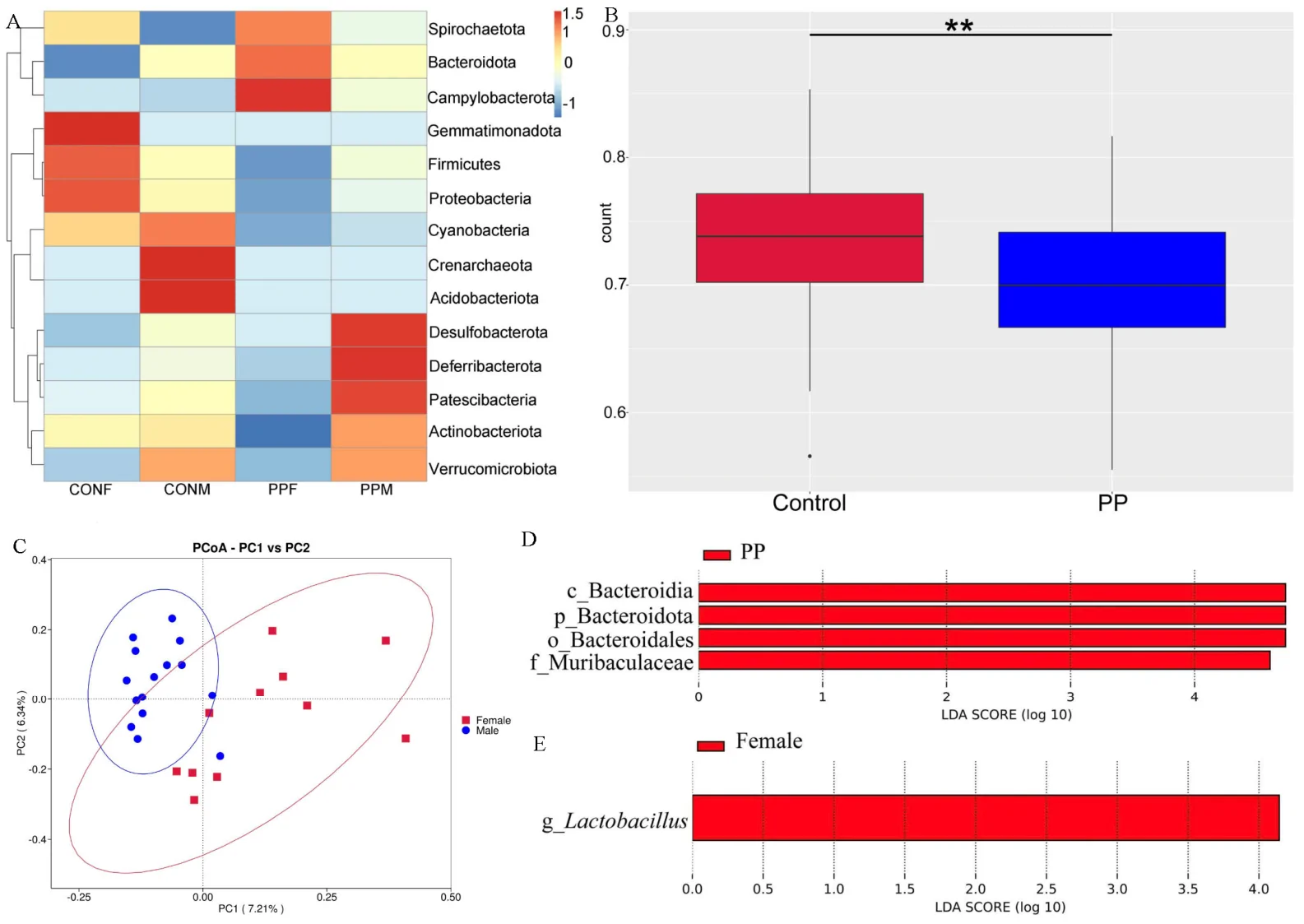
The composition of gut microbiota at the phylum level (**A**). Wilcoxon Rank-Sum test of beta diversity between the PP and control groups based on unweighted Unifrac distance (**B**). PCoA analysis between males and females based on the Jaccard distance (**C**). LEfSe analysis between PP and control groups (**D**), and between males and females (**E**). The symbol (**) indicates *p* < 0.01.

The dominant phyla in the gut microbiota of mice include Firmicutes, Bacteroidetes, Proteobacteria, and Actinobacteria (Figure 5A). The dominant families are Lachnospiraceae, Lactobacillaceae, Muribaculaceae, Prevotellaceae, Eggerthellaceae (Supplementary Information S9A). The genera *Lactobacillus*, *Enterobacter*, *Ligilactobacillus*, and *Brachyspira* are relatively abundant (Supplementary Information S9B). The analysis of the differences in the alpha diversity index between groups demonstrated that there were no significant differences between the PP group and the control group, nor between females and males (Supplementary Information S10).

The results of the Wilcoxon Rank-Sum test based on unweighted Unifrac distance showed that there was a significant difference in the beta diversity of the gut microbiota between the PP and control groups (Figure 5B). The result of the PCoA analysis, based on the Jaccard distance, showed a significant separation between males’ and females’ samples (Figure 5C). This result suggested that there were differences in the community diversity of the gut microbiota between males and females. The results of MRPP analysis revealed that the gut microbiota was significantly affected by sex but not by PP-MPs (Table 1). The LEfSe analysis results indicated that the biomarkers with statistical differences in the PP group were the family Muribaculaceae, class Bacteroidia, phylum Bacteroidota, and order Bacteroidales (Figure 5D). The biomarker with significant differences in females was the genus *Lactobacillus* (Figure 5E).

### 3.8. Correlation Analysis

**Figure 6:**
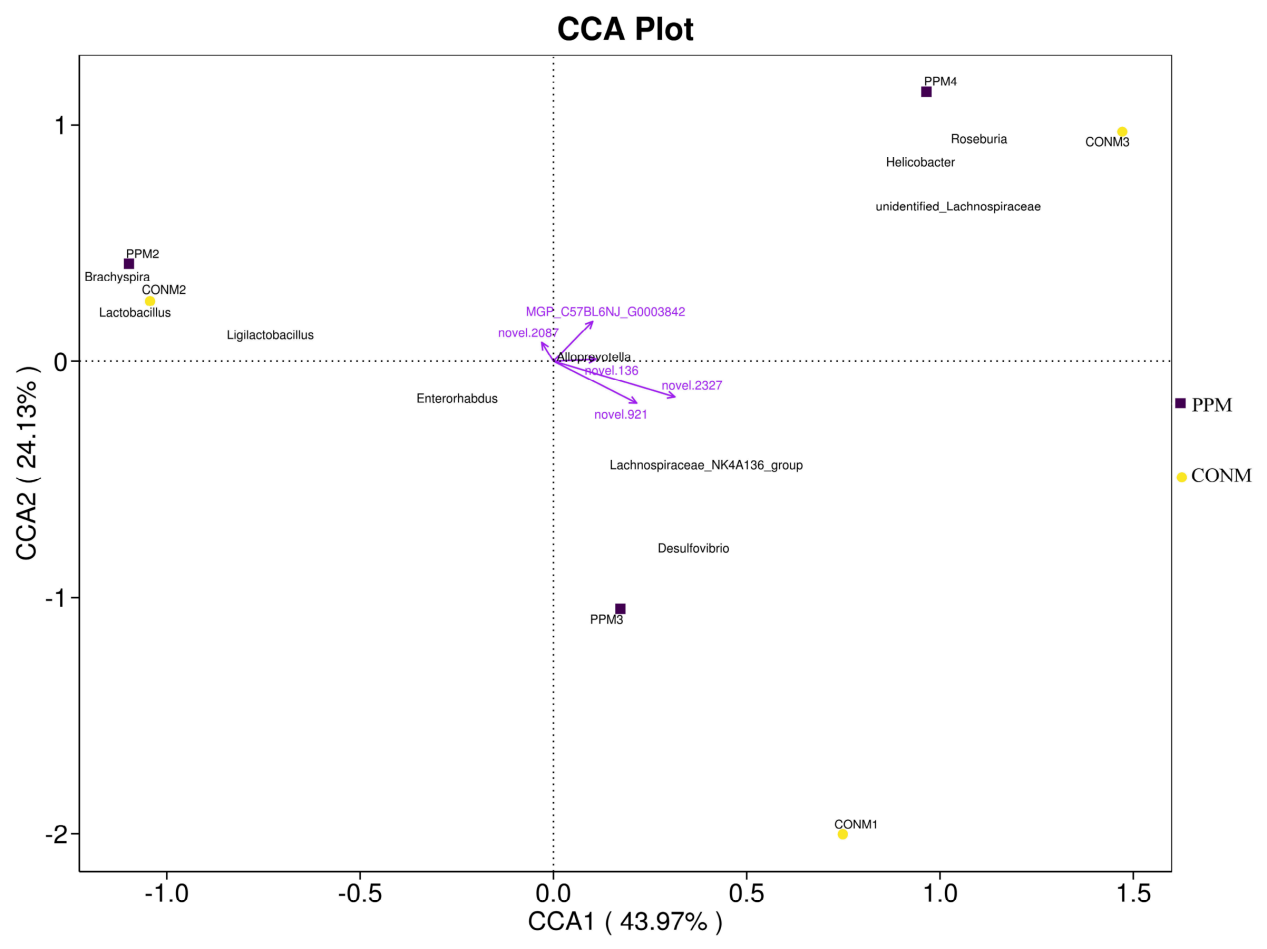
Canonical correlation analysis between the gut microbiota and gene expression. DEGs are denoted by arrows. The length of the arrow represents the strength of its correlation with the gut microbiota. Longer arrows indicate stronger correlations, and shorter arrows indicate weaker correlations.

This study indicated a significant relationship between the gut microbiota and gene expression in the testicles. *Candidatus Arthromitus* showed a significant positive correlation with the following genes: *novel.2639*, *novel.2646*, *novel.907*, *novel.964*, *novel.1853*, *MGP_C57BL6NJ_G0028099*, and *MGP_C57BL6NJ_G0017705*. The genus *ASF356* was significantly positively related to *novel.2053*, *novel.1878*, and *MGP_C57BL6NJ_G0021703*. *Lachnoclostridium* was significantly positively related to *novel.1639*, *MGP_C57BL6NJ_G0037448* (*Gm42868*), *MGP_C57BL6NJ_G0020130*, *MGP_C57BL6NJ_G0017705* (*Rufy2*), *MGP_C57BL6NJ_G0016252* (*Gdap1*), *MGP_C57BL6NJ_G0006432* (*Gm43053*). A significant positive correlation was observed between *Butyricoccus* and genes *MGP_C57BL6NJ_G0001464*, *MGP_C57BL6NJ_G0002012*, *MGP_C57BL6NJ_G0006869* (*Gm42916*). The *Parvibacter* genus has been found to be positively associated with the *MGP_C57BL6NJ_G0020130* gene (Supplementary Information S11). The CCA results indicated that the *novel.2327* and *novel.921* genes exhibited a higher correlation with the gut microbiota (Figure 6). The gene *novel.2327* exhibited a significantly negative correlation with *Dubosiella* and *Erysipelatoclostridium*, while exhibiting a significantly positive correlation with *Butyricicoccus*. The gene *novel.921* exhibited a significant positive correlation with *Butyricicoccus*. Based on KEGG enrichment analysis, the *MGP_C57BL6NJ_G0027419* (*Col9a3*) and *MGP_C57BL6NJ_G0023962* (*Col11a2*) genes were associated with the protein digestion and absorption pathway. *MGP_C57BL6NJ_G0027419* exhibited a negative correlation with *Candidatus Saccharimonas* and *Rikenella*, while *MGP_C57BL6NJ_G0023962* exhibited a positive correlation with *Jeotgalicoccus* and *Staphylococcuz*.

## 4. Discussion

Microplastics, as emerging pollutants, are pervasive in daily life, including water, air, household goods, and industrial products [37,38]. Their ability to accumulate in organisms through the food chain poses serious health risks [38,39]. PP-MPs are particularly relevant to daily life, found in terms like disposable take-out food containers and baby bottles [32,40,41]. A study on microplastic exposure among the Indonesian farming community identified polypropylene as the most common plastic pollutant to which individuals were exposed [42]. Thus, investigating the effects of PP-MPs on immunity and reproduction is vital for safeguarding animal health and ecological security.

Microplastics induce mechanical damage to organs, such as the spleen and liver, trigger inflammatory responses in immune cells and tissues, and alter the immune status of organisms [43,44,45]. However, our study found that exposure to PP-MPs for five weeks does not significantly affect body mass, wet immune organ mass, or the serum levels of IgG, IL-4, and IFN-γ. This suggests that exposure to PP-MPs does not significantly alter immune status in mice. Previous studies have shown that continuous exposure to PP-MPs for 4 weeks did not affect body mass, food intake, organ mass, or the histopathology of the heart, lung, and other tissues in ICR mice [46]. Additionally, no significant changes were observed in body mass, food intake, or relative organ mass of mice after 14 weeks of exposure to PS-MPs [47]. The toxicity of microplastics is closely related to both exposure time and concentration [48,49]. It is evident that short-term exposure to microplastics may not cause significant changes in humoral immunity [50].

Environmental pollution is recognized as an important factor influencing male reproductive health [51]. Long-term exposure to microplastics has been demonstrated to reduce sperm motility and cause abnormal morphology in male rats, decrease the number of follicles in female mice, and disrupt serum hormone levels, thereby impairing reproductive function [8,52]. For instance, PS-MPs have been shown to provoke severe testicular inflammation, decreasing testosterone levels and sperm quality [7,53]. PVC microplastics induce a significant reduction in the lumen diameter of the seminiferous tubules and increase the sperm deformity rate [54]. Furthermore, long-term exposure to PET microplastics adversely affected sperm maturation by down-regulating the *Meiosin* gene [55]. However, our study observed no adverse effects of PP-MPs on testicular morphology and testosterone levels in male mice.

However, GSEA revealed that PP-MPs led to the downregulation of 242 gene sets, such as pheromone receptor activity, the G protein coupled receptor (GPCR) signaling pathway, and olfactory receptor (OR) activity. Pheromone signal detection is essential for animals to display sex-specific behaviors [56]. The downregulation of genes associated with pheromone receptor activity may impair the ability of mice to distinguish pheromones from both the same and opposite sexes, disrupting their courtship and mating behaviors [57]. Furthermore, the downregulation of GPCR signaling may pose a potential threat to reproductive physiology. The ORs, a class of GPCRs, are widely expressed in various tissues [58]. In sperm, the OR can mediate chemotaxis toward the oocyte [59]. GPCRs play a pivotal role in cellular communication in physiological systems [60]. Class A GPCRs are known regulators of reproductive function, including spermatogenesis, sperm motility, the acrosome reaction, and testosterone-mediated signaling [59,61,62,63]. GPR30 was an important regulator of seasonal testicular activity in *Myodes glareolus* [64]. Barut et al. found that GPER is positively related to sperm motility and morphology, suggesting that its downregulation may be associated with male infertility [65]. Therefore, we propose that PP-MPs may disrupt mating behavior and impair reproductive capacity in male mice by downregulating the expression of genes associated with pheromone receptors and G protein-coupled receptor signaling pathways.

Among the gene sets annotated to the KEGG database, 173 gene sets were downregulated in the PP group, including lipoic acid metabolism, protein digestion and absorption, and tyrosine metabolism. The protein digestion and absorption pathway was very important for testicular development and spermatogenesis [66,67,68]. We found that the collagen genes (*Col9a3*, *Col11a2*, *Col27a1*, *Col26a1*, *Col7a1*) played a key role in the downregulation of this pathway. Both *Col9a3* and *Col11a2* are regulated by the transcription factor *Sox 9* and play crucial roles in testis development [69,70,71]. *Sox9* is an important factor in regulating testicular differentiation, and it remains expressed in adults [72,73]. *Sox9* not only maintains the structure of adult male testis but is also crucial for spermatogenesis [74]. Since *Col9a3* and *Col11a2* are targets of *Sox9*, the downregulation of *Col9a3* and *Col11a2* gene expression may indicate reduced *Sox9* activity, which may cause reproductive system disorders in mice from the PP group [71].

We hypothesized that the gut microbiota plays a significant role in the reproductive toxicity induced by PP-MPs. Therefore, we analyzed the diversity of the gut microbiota and the association between testicular gene expression and gut microbiota. Microplastics have been shown to cause intestinal dysfunction, altering the intestinal microecological environment and affecting the community structure and diversity of the gut microbiota [75]. In this study, we found that PP-MPs significantly altered the community structure of the gut microbiota, rather than its abundance and diversity. Previous studies on microplastics exposure have yielded similar results [25,47,76]. For example, exposure to PS-MPs with a particle size of 0.5 and 5 μm did not cause significant differences in the Shannon and Simpson indices of the gut microbiota in C57BL/6 mice [76]. PVC microplastics did not significantly affect the Shannon index of gut microbiota in New Zealand white rabbits [25]. But microplastics have been confirmed to disrupt the dynamic balance of gut microbiota such as Firmicutes, Bacteroidetes, Actinobacteriota, and Proteobacteria [26,77,78,79,80]. The Firmicutes and Bacteroidetes are pivotal members in maintaining intestinal homeostasis and host health [81]. After exposure to microplastics, the ratio of Firmicutes to Bacteroides was imbalanced [82,83]. Short-term (7-day) exposure to PE, PET, and PP microplastics increased the F/B ratio in the gut microbiota of mice [84]. Exposure to PS-MPs for 4 weeks significantly reduced the F/B ratio in the mice [83]. Exposure to polyethylene terephthalate (PET) microplastics for 28 days the ratio of F/B in the gut microbiota of pigs increased. However, the continuous intake of high-dose PET microplastics might mitigate this situation [85]. We found that the F/B ratio was higher in female mice than in males, which was the same as previous research [81]. After exposure to PP-MPs, the F/B ratio in the gut microbiota decreased in both male (7.42%) and female mice (91.75%), and the reduction was more pronounced in females. The genus *Bacteroida* was the statistically significant difference biomarker in the PPF group. However, no statistically significant biomarkers were found in the male groups. Thus, the effects of PP-MPs on the gut microbiota of female mice were more significant than those on males. The relative abundance of *Lactobacillus* in the CONF group was higher than in the PPF group, and similar findings were observed in males. *Lactobacillus* is a common probiotic belonging to the phylum Firmicutes [86], which plays a significant role in improving gastrointestinal inflammation, regulating host immunity and nutrient metabolism [87]. *Lactobacillus acidophilus* and *Lactobacillus plantarum* have been observed to stimulate the expression of IL-10 and IL-17F in marron (*Cherax cainii*), thereby regulating their inflammatory response [88]. Moreover, *Lactobacillus* can produce L-ascorbic acid, which plays a vital role in human health and longevity [89]. It was evident that PP-MPs may induce gut microbiota dysbiosis, leading to a reduction in the relative abundance of beneficial bacteria.

The gut microbiota has been demonstrated to regulate testicular function via the gut-testis axis [90]. To further analyze the role of gut microbiota in reproductive toxicity induced by microplastics, a correlation analysis was conducted between gut microbiota and DEGs. We found that the *Col9a3* gene expression was negatively related to *Candidatus_Saccharimonas* and *Rikenella*. The expression of the *Col11a2* gene showed a significant positive correlation with *Jeotgalicoccus* and *Staphylococcus*. *Jeotgalicoccus* is the dominant genus in the testis and epididymis, which is closely related to male reproduction [91]. Gut microbiota imbalance can affect testicular development and testosterone levels, alter the morphology of testicular tissue, and impair male reproductive function [92,93,94]. Therefore, we speculate that the gut microbiota may be associated with gene expression patterns in the testes of male mice.

## 5. Conclusions

In conclusion, PP-MPs exposure did not significantly affect humoral immunity (anti-KLH IgG). For cellular immunity, PP-MPs exposure reduced the PHA response in females, whereas no effect was observed in males. Additionally, exposure to PP-MPs differently altered the community structure of the gut microbiota in male and female mice. Notably, a reduction in the relative abundance of the probiotic *Lactobacillus* was observed in both sexes, although this did not reach statistical significance. Furthermore, we found that PP-MPs exposure altered gene expression in the testis of male mice. Pheromone receptor activity, G protein-coupled receptor signaling pathway, and protein digestion and absorption pathways were downregulated after exposure to microplastics. The downregulation of the protein digestion and absorption pathway was primarily mediated by collagen genes, including *Col9a3*, *Col11a2*, *Col27a1*, *Col26a1*, and *Col7a1*. These genes downregulation might be associated with the gut microbiota. Supporting this, the *Col9a3* gene was negatively related to *Candidatus_Saccharimonas* and *Rikenella*. The expression of the *Col11a2* gene was positively related to *Jeotgalicoccus* and *Staphylococcuz*. Our findings suggest that exposure to PP-MPs may impair the reproductive function of male mice by downregulating the expression of collagen genes, and the gut microbiota may be involved in this process. While the present study assessed the effects of PP-MPs on reproductive function solely based on testicular gene expression, the associated physiological phenotypic changes were not examined and thus need further investigation. Furthermore, the role of the gut microbiota has not been verified through fecal microbiota transplantation (FMT). Therefore, we will further explore the impact of PP-MPS on immune and reproductive functions through multi-omics integration and FMT methods.

## Figures and Tables

**Table 1:** The MRPP differences analysis between groups. The significance value less than 0.05 indicates a statistically significant difference.

Group	A	Observed-Delta	Expected-Delta	Significance
Control vs. PP	0.002	0.721	0.723	0.313
Female vs. Male	0.013	0.714	0.723	0.043

## Data Availability

The original contributions presented in this study are included in the article/Supplementary Materials. Further inquiries can be directed to the corresponding author. The sequence data for 16S amplicons and transcriptomes are available from NCBI (https://www.ncbi.nlm.nih.gov/, accessed on 24 July 2026) under accession numbers: PRJNA1406490 and PRJNA1411278.

## Supplementary Materials

The following supporting information can be downloaded at: https://www.mdpi.com/article/10.3390/toxics14080679/s1, Supplementary Information S1: Method of Humoral Immunity Assays; Supplementary Information S2: The bar chart of GO enrichment analysis; Supplementary Information S3: GSEA of enriched GO Terms in the PP group; Supplementary Information S4: GSEA of enriched GO Terms in the control group; Supplementary Information S5: GSEA of enriched KEGG Terms in the PP group; Supplementary Information S6: GSEA of enriched KEGG Terms in the control group; Supplementary Information S7: The species rarefaction curve indicates that the sequencing data volume was sufficient; Supplementary Information S8: Statistical results of data processing; Supplementary Information S9: The composition of gut microbiota at the family (A), and genus (B) levels in each group; Supplementary Information S10: The *p* values of the Wilcoxon Rank-Sum test for the alpha diversity index between the PP and the control groups, as well as between males and females; Supplementary Information S11: The correlation between gut microbiota and DEGs.
