# Supplementary material for: Possible Role of Gut Microbiota in Polypropylene Microplastics-Induced Immunity and Reproductive Dysfunction in Mice
Source: Toxics. 2026 Jul 31;14(8):679. doi: 10.3390/toxics14080679 (PMC13517389; doi:10.3390/toxics14080679)
Supplement: Supplementary file 1 [file toxics-14-00679-s001.zip › Supplementary Information S5.pdf]

Supplementary Information S5. GSEA of enriched KEGG Terms in the PP group.

NAME	SIZE	ES	NES	NOM p-value	FDR q-value
AMINOACYL_TRNA_BIOSYNTHESIS(MMU00970)	47	0.417691	1.624923	0	0.550568
CITRATE_CYCLE_(TCA_CYCLE)(MMU00020)	35	0.533821	1.60827	0	0.376416
SELENOCOMPOUND_METABOLISM(MMU00450)	18	0.566341	1.541761	0	0.669941
GLYCOSPHINGOLIPID_BIOSYNTHESIS_GANGLIO_SERIES(MMU00604)	16	0.55211	1.523191	0	0.574095
MUCIN_TYPE_O_GLYCAN_BIOSYNTHESIS(MMU00512)	33	0.485176	1.515568	0	0.484147
OOCYTE_MEIOSIS(MMU04114)	138	0.327956	1.513624	0	0.429127
PENTOSE_PHOSPHATE_PATHWAY(MMU00030)	41	0.461483	1.451136	0	0.647541
PROTEIN_PROCESSING_IN_ENDOPLASMIC_RETICULUM(MMU04141)	206	0.353204	1.427837	0	0.697302
BUTANOATE_METABOLISM(MMU00650)	26	0.566178	1.414397	0.274793	0.646399
PURINE_METABOLISM(MMU00230)	151	0.303819	1.339392	0	1
MTOR_SIGNALING_PATHWAY(MMU04150)	169	0.36764	1.29488	0.17505	1
ALANINE__ASPARTATE_AND_GLUTAMATE_METABOLISM(MMU00250)	43	0.353481	1.291389	0.179752	1
CELL_CYCLE(MMU04110)	183	0.242956	1.28889	0.177645	1
PANTOTHENATE_AND_COA_BIOSYNTHESIS(MMU00770)	21	0.358831	1.259882	0.097713	1
VALINE__LEUCINE_AND_ISOLEUCINE_DEGRADATION(MMU00280)	59	0.380985	1.251477	0.082495	1
ENDOCRINE_AND_OTHER_FACTOR_REGULATED_CALCIUM_REABSORPTION(MMU04961)	69	0.365921	1.249373	0	1
N_GLYCAN_BIOSYNTHESIS(MMU00510)	53	0.328671	1.245777	0.176471	1
AUTOPHAGY_OTHER(MMU04136)	38	0.330831	1.216942	0.407787	1
RENAL_CELL_CARCINOMA(MMU05211)	76	0.308861	1.211707	0.17505	1
FC_GAMMA_R_MEDIATED_PHAGOCYTOSIS(MMU04666)	109	0.319246	1.210793	0.176471	1
VARIOUS_TYPES_OF_N_GLYCAN_BIOSYNTHESIS(MMU00513)	42	0.324063	1.197534	0.17505	1
PROGESTERONE_MEDIATED_OOCYTE_MATURATION(MMU04914)	110	0.282338	1.179833	0.180527	1
LONG_TERM_POTENTIATION(MMU04720)	78	0.31204	1.165206	0.207585	1
AUTOPHAGY_ANIMAL(MMU04140)	212	0.236147	1.156591	0.17505	1
MELANOGENESIS(MMU04916)	99	0.360289	1.147335	0.287726	1
NUCLEOTIDE_METABOLISM(MMU01232)	97	0.258923	1.146582	0.198039	1
FATTY_ACID_ELONGATION(MMU00062)	33	0.326378	1.139019	0.220039	1
TERPENOID_BACKBONE_BIOSYNTHESIS(MMU00900)	24	0.37269	1.133939	0.358149	1
ALDOSTERONE_REGULATED_SODIUM_REABSORPTION(MMU04960)	38	0.392294	1.12883	0.195171	1
TOXOPLASMOSIS(MMU05145)	120	0.277273	1.126343	0.303983	1
COMPLEMENT_AND_COAGULATION_CASCADES(MMU04610)	90	0.312138	1.125244	0.290448	1
LONGEVITY_REGULATING_PATHWAY_MULTIPLE_SPECIES(MMU04213)	74	0.278642	1.11869	0.201663	1
PYRIMIDINE_METABOLISM(MMU00240)	61	0.304828	1.107936	0.395112	1
TGF_BETA_SIGNALING_PATHWAY(MMU04350)	118	0.288717	1.078888	0.303983	1
LYSOSOME(MMU04142)	145	0.295781	1.078507	0.29979	1
PROTEOGLYCANS_IN_CANCER(MMU05205)	231	0.275454	1.066635	0.287726	1
B_CELL_RECEPTOR_SIGNALING_PATHWAY(MMU04662)	82	0.2403	1.058916	0.498986	1
SPHINGOLIPID_METABOLISM(MMU00600)	54	0.303329	1.058062	0.287726	1
GLYCOSAMINOGLYCAN_DEGRADATION(MMU00531)	23	0.34599	1.049277	0.278826	1
LONG_TERM_DEPRESSION(MMU04730)	66	0.30798	1.045889	0.278826	1
NUCLEOCYTOPLASMIC_TRANSPORT(MMU03013)	168	0.21507	1.039162	0.283859	1
OVARIAN_STEROIDOGENESIS(MMU04913)	52	0.356644	1.038827	0.359504	1
FC_EPSILON_RI_SIGNALING_PATHWAY(MMU04664)	70	0.245399	1.032171	0.49	1
PROSTATE_CANCER(MMU05215)	121	0.272126	1.028399	0.294118	1

NAME	SIZE	ES	NES	NOM p-value	FDR q-value
ADHERENS_JUNCTION(MMU04520)	119	0.209541	1.019547	0.378	1
NEUROTROPHIN_SIGNALING_PATHWAY(MMU04722)	133	0.268287	1.013714	0.384306	1
EGFR_TYROSINE_KINASE_INHIBITOR_RESISTANCE(MMU01521)	89	0.283231	1.012275	0.400419	1
CHEMOKINE_SIGNALING_PATHWAY(MMU04062)	185	0.244828	1.010917	0.195171	1
GLIOMA(MMU05214)	86	0.273531	0.999427	0.496982	1
ERBB_SIGNALING_PATHWAY(MMU04012)	92	0.246173	0.997511	0.384306	1
PROLACTIN_SIGNALING_PATHWAY(MMU04917)	75	0.293872	0.992093	0.584179	1
SALIVARY_SECRETION(MMU04970)	86	0.318382	0.991285	0.496982	1
STEROID_BIOSYNTHESIS(MMU00100)	24	0.415398	0.989277	0.381053	1
THYROID_HORMONE_SIGNALING_PATHWAY(MMU04919)	147	0.260566	0.988731	0.382294	1
ACUTE_MYELOID_LEUKEMIA(MMU05221)	76	0.314343	0.979337	0.382536	1
PROPANOATE_METABOLISM(MMU00640)	41	0.269525	0.979225	0.478873	1
AXON_GUIDANCE(MMU04360)	197	0.305883	0.976609	0.503145	1
GNRH_SIGNALING_PATHWAY(MMU04912)	94	0.284204	0.973753	0.466801	1
CHRONIC_MYELOID_LEUKEMIA(MMU05220)	88	0.282611	0.972075	0.579477	1
MITOPHAGY_ANIMAL(MMU04137)	140	0.166961	0.970335	0.507634	1
INSULIN_SIGNALING_PATHWAY(MMU04910)	151	0.26946	0.960926	0.513078	1
VASCULAR_SMOOTH_MUSCLE_CONTRACTION(MMU04270)	154	0.251608	0.956467	0.51782	1
CARBOHYDRATE_DIGESTION_AND_ABSORPTION(MMU04973)	47	0.31055	0.956168	0.482897	1
COLORECTAL_CANCER(MMU05210)	106	0.221283	0.955266	0.513078	1
FRUCTOSE_AND_MANNOSE_METABOLISM(MMU00051)	51	0.242818	0.951295	0.51927	1
APELIN_SIGNALING_PATHWAY(MMU04371)	153	0.283378	0.948205	0.466801	1
SALMONELLA_INFECTION(MMU05132)	316	0.178235	0.944718	0.613095	1
GLYCOSAMINOGLYCAN_BIOSYNTHESIS_CHONDROITIN_SULFATE_DERMATAN_SULFATE(MMU00532)	23	0.348104	0.944199	0.382536	1
PHOSPHOLIPASE_D_SIGNALING_PATHWAY(MMU04072)	157	0.25689	0.94155	0.603774	1
P53_SIGNALING_PATHWAY(MMU04115)	97	0.228822	0.936043	0.693712	1
HEPATOCELLULAR_CARCINOMA(MMU05225)	195	0.256506	0.935748	0.471311	1
HIF_1_SIGNALING_PATHWAY(MMU04066)	140	0.213992	0.927242	0.478873	1
GASTRIC_ACID_SECRETION(MMU04971)	89	0.282322	0.921065	0.720322	1
MICRORNAS_IN_CANCER(MMU05206)	185	0.239274	0.920731	0.584179	1
MISMATCH_REPAIR(MMU03430)	26	0.250928	0.915836	0.506098	1
FATTY_ACID_METABOLISM(MMU01212)	73	0.284926	0.915093	0.713996	1
BIOSYNTHESIS_OF_UNSATURATED_FATTY_ACIDS(MMU01040)	37	0.277761	0.914683	0.698113	1
RIBOSOME_BIOGENESIS_IN_EUKARYOTES(MMU03008)	90	0.21576	0.913362	0.611328	1
ALDOSTERONE_SYNTHESIS_AND_SECRETION(MMU04925)	104	0.2927	0.912606	0.382536	1
BIOSYNTHESIS_OF_AMINO_ACIDS(MMU01230)	109	0.213331	0.905699	0.688017	1
PROXIMAL_TUBULE_BICARBONATE_RECLAMATION(MMU04964)	24	0.304588	0.900269	0.585317	1
PHOSPHATIDYLINOSITOL_SIGNALING_SYSTEM(MMU04070)	106	0.261987	0.89652	0.694387	1
NON_SMALL_CELL_LUNG_CANCER(MMU05223)	85	0.239057	0.8962	0.456349	1
HEPATITIS_B(MMU05161)	167	0.213061	0.895113	0.579477	1
OTHER_TYPES_OF_O_GLYCAN_BIOSYNTHESIS(MMU00514)	46	0.251253	0.890443	0.480885	1
LONGEVITY_REGULATING_PATHWAY(MMU04211)	100	0.239097	0.889818	0.598753	1
CUSHING_SYNDROME(MMU04934)	170	0.273031	0.883343	0.47817	1
CHOLINE_METABOLISM_IN_CANCER(MMU05231)	113	0.217625	0.882385	0.482328	1
FOXO_SIGNALING_PATHWAY(MMU04068)	148	0.19762	0.881067	0.482328	1
CARBON_METABOLISM(MMU01200)	158	0.195865	0.880989	0.675676	1

NAME	SIZE	ES	NES	NOM p-value	FDR q-value
GASTRIC_CANCER(MMU05226)	155	0.233129	0.880369	0.47817	1
RNA_POLYMERASE(MMU03020)	42	0.188213	0.880119	0.706767	1
INFLAMMATORY_MEDIATOR_REGULATION_OF_TRP_CHANNELS(MMU04750)	121	0.240339	0.87627	0.615694	1
OTHER_GLYCAN_DEGRADATION(MMU00511)	19	0.33875	0.876236	0.480167	1
RENIN_SECRETION(MMU04924)	82	0.254889	0.874874	0.607967	1
HEPATITIS_C(MMU05160)	173	0.186638	0.872712	0.894523	1
ETHER_LIPID_METABOLISM(MMU00565)	53	0.185939	0.871456	0.665263	1
PANCREATIC_CANCER(MMU05212)	89	0.224699	0.870989	0.598753	1
GAP_JUNCTION(MMU04540)	111	0.238935	0.869245	0.482328	1
FATTY_ACID_BIOSYNTHESIS(MMU00061)	21	0.27417	0.868289	0.713996	1
PRIMARY_BILE_ACID_BIOSYNTHESIS(MMU00120)	18	0.352092	0.868256	0.495183	1
GLUCAGON_SIGNALING_PATHWAY(MMU04922)	125	0.22229	0.855634	0.595573	1
NITROGEN_METABOLISM(MMU00910)	21	0.338322	0.855048	0.698545	1
EFFEROCYTOSIS(MMU04148)	168	0.22954	0.854914	0.498961	1
PLATINUM_DRUG_RESISTANCE(MMU01524)	98	0.185959	0.854821	0.696017	1
DRUG_METABOLISM_OTHER_ENZYMES(MMU00983)	98	0.243667	0.853515	0.892562	1
RENIN_ANGIOTENSIN_SYSTEM(MMU04614)	37	0.253577	0.853413	0.693642	1
SPHINGOLIPID_SIGNALING_PATHWAY(MMU04071)	131	0.21369	0.847818	0.584179	1
FERROPTOSIS(MMU04216)	56	0.213973	0.83803	0.566	1
HIPPO_SIGNALING_PATHWAY_MULTIPLE_SPECIES(MMU04392)	31	0.279315	0.836141	0.802817	1
NF_KAPPA_B_SIGNALING_PATHWAY(MMU04064)	99	0.226491	0.834442	0.680328	1
SMALL_CELL_LUNG_CANCER(MMU05222)	111	0.214689	0.823657	0.579477	1
PERTUSSIS(MMU05133)	82	0.207417	0.817313	0.787402	1
CENTRAL_CARBON_METABOLISM_IN_CANCER(MMU05230)	93	0.254368	0.813274	0.802817	1
SIGNALING_PATHWAYS_REGULATING_PLURIPOTENCY_OF_STEM_CELLS(MMU04550)	148	0.232889	0.805956	0.694387	1
GROWTH_HORMONE_SYNTHESIS__SECRETION_AND_ACTION(MMU04935)	123	0.267455	0.803084	0.70825	1
CGMP_PKG_SIGNALING_PATHWAY(MMU04022)	203	0.205766	0.797255	0.672032	1
METABOLIC_PATHWAYS(MMU01100)	1791	0.15276	0.794925	0.891892	1
PYRUVATE_METABOLISM(MMU00620)	64	0.202956	0.792177	0.893443	1
PATHWAYS_IN_CANCER(MMU05200)	587	0.200575	0.791225	0.694387	1
HISTIDINE_METABOLISM(MMU00340)	25	0.264832	0.791131	0.807851	1
PPAR_SIGNALING_PATHWAY(MMU03320)	99	0.210094	0.788341	0.800805	1
GLYCOSYLPHOSPHATIDYLINOSITOL_(GPI)_ANCHOR_BIOSYNTHESIS(MMU00563)	28	0.221685	0.783078	0.68472	1
MINERAL_ABSORPTION(MMU04978)	56	0.227528	0.772402	0.795082	1
VIRAL_CARCINOGENESIS(MMU05203)	240	0.172007	0.763842	0.895372	1
MOTOR_PROTEINS(MMU04814)	287	0.18878	0.763574	0.895372	1
VASOPRESSIN_REGULATED_WATER_REABSORPTION(MMU04962)	45	0.233104	0.76304	0.890985	1
TUBERCULOSIS(MMU05152)	177	0.172688	0.762415	0.890985	1
AMPK_SIGNALING_PATHWAY(MMU04152)	141	0.215213	0.760038	0.677485	1
PROTEIN_EXPORT(MMU03060)	42	0.193384	0.759941	0.791908	1
PORPHYRIN_METABOLISM(MMU00860)	39	0.198273	0.755184	0.785124	1
ENDOMETRIAL_CANCER(MMU05213)	68	0.211365	0.751563	0.70825	1
CORTISOL_SYNTHESIS_AND_SECRETION(MMU04927)	75	0.272174	0.750337	0.694387	1

NAME	SIZE	ES	NES	NOM p-value	FDR q-value
INSULIN_SECRETION(MMU04911)	87	0.256324	0.745679	0.895372	1
DNA_REPLICATION(MMU03030)	43	0.17617	0.739073	0.898039	1
INOSITOL_PHOSPHATE_METABOLISM(MMU00562)	79	0.241539	0.734486	0.895372	1
THYROID_HORMONE_SYNTHESIS(MMU04918)	76	0.224693	0.720419	0.698545	1
ABC_TRANSPORTERS(MMU02010)	54	0.225382	0.719469	0.672032	1
THYROID_CANCER(MMU05216)	41	0.200137	0.718919	0.698545	1
TNF_SIGNALING_PATHWAY(MMU04668)	117	0.176462	0.711816	0.780933	1
MALARIA(MMU05144)	47	0.226582	0.706814	0.890985	1
BREAST_CANCER(MMU05224)	157	0.199627	0.7065	0.893443	1
CAMP_SIGNALING_PATHWAY(MMU04024)	236	0.187513	0.69887	0.895372	1
SYSTEMIC_LUPUS_ERYTHEMATOSUS(MMU05322)	116	0.188053	0.697592	0.902256	1
ENDOCRINE_RESISTANCE(MMU01522)	102	0.202463	0.696762	0.713996	0.996245
AMINO_SUGAR_AND_NUCLEOTIDE_SUGAR_METABOLISM(MMU00520)	59	0.131565	0.689682	0.89495	0.996263
PARATHYROID_HORMONE_SYNTHESIS__SECRETION_AND_ACTION(MMU04928)	111	0.203044	0.686368	0.800805	0.993119
GLUTATHIONE_METABOLISM(MMU00480)	81	0.18356	0.682779	0.896825	0.989679
ADIPOCYTOKINE_SIGNALING_PATHWAY(MMU04920)	79	0.200102	0.662902	0.895372	0.994474
BACTERIAL_INVASION_OF_EPITHELIAL_CELLS(MMU05100)	94	0.153727	0.654479	0.895372	0.995244
TRANSCRIPTIONAL_MISREGULATION_IN_CANCER(MMU05202)	198	0.155405	0.652803	0.891892	0.99146
CHOLESTEROL_METABOLISM(MMU04979)	59	0.215904	0.648033	0.891892	0.987917
CHOLINERGIC_SYNAPSE(MMU04725)	123	0.20081	0.631149	0.895372	0.992629
MANNOSE_TYPE_O_GLYCAN_BIOSYNTHESIS(MMU00515)	20	0.19091	0.62933	0.765657	0.988965
HEDGEHOG_SIGNALING_PATHWAY(MMU04340)	58	0.190007	0.623572	0.794549	0.986372
CIRCADIAN_ENTRAINMENT(MMU04713)	103	0.196293	0.62139	0.895372	0.982114
PROTEASOME(MMU03050)	63	0.155311	0.616388	0.902256	0.978546
BETA_ALANINE_METABOLISM(MMU00410)	34	0.199235	0.592342	0.888889	0.983151
NOTCH_SIGNALING_PATHWAY(MMU04330)	74	0.163202	0.533319	0.895372	0.99558
BIOSYNTHESIS_OF_NUCLEOTIDE_SUGARS(MMU01250)	45	0.105343	0.485268	0.899807	0.996901
