# Supplementary material for: Possible Role of Gut Microbiota in Polypropylene Microplastics-Induced Immunity and Reproductive Dysfunction in Mice
Source: Toxics. 2026 Jul 31;14(8):679. doi: 10.3390/toxics14080679 (PMC13517389; doi:10.3390/toxics14080679)
Supplement: Supplementary file 1 [file toxics-14-00679-s001.zip › Supplementary Information S6.pdf]

Supplementary Information S6. GSEA of enriched KEGG Terms in the control group.

| NAME | SIZE | ES | NES | NOM p-value | FDR q-value |
|---|---|---|---|---|---|
| LIPOIC_ACID_METABOLISM(MMU00785) | 24 | -0.40819 | -1.54952 | 0 | 1 |
| PROTEIN_DIGESTION_AND_ABSORPTION(MMU04974) | 106 | -0.51071 | -1.51316 | 0 | 1 |
| PRIMARY_IMMUNODEFICIENCY(MMU05340) | 40 | -0.4613 | -1.47203 | 0 | 1 |
| TYROSINE_METABOLISM(MMU00350) | 44 | -0.45741 | -1.44657 | 0 | 1 |
| SNARE_INTERACTIONS_IN_VESICULAR_TRANSPORT(MMU04130) | 36 | -0.41983 | -1.42332 | 0 | 1 |
| DIABETIC_CARDIOMYOPATHY(MMU05415) | 268 | -0.25128 | -1.40282 | 0 | 1 |
| NICOTINATE_AND_NICOTINAMIDE_METABOLISM(MMU00760) | 44 | -0.35815 | -1.38519 | 0 | 1 |
| TIGHT_JUNCTION(MMU04530) | 229 | -0.2988 | -1.3851 | 0 | 0.993204 |
| TRYPTOPHAN_METABOLISM(MMU00380) | 49 | -0.38269 | -1.38429 | 0 | 0.892584 |
| GLYOXYLATE_AND_DICARBOXYLATE_METABOLISM(MMU00630) | 40 | -0.41568 | -1.35576 | 0.108949 | 0.982159 |
| SYNAPTIC_VESICLE_CYCLE(MMU04721) | 85 | -0.35393 | -1.35156 | 0 | 0.938709 |
| ONE_CARBON_POOL_BY_FOLATE(MMU00670) | 22 | -0.40736 | -1.34493 | 0 | 0.946197 |
| OLFACTORY_TRANSDUCTION(MMU04740) | 298 | -0.44374 | -1.33586 | 0 | 0.921588 |
| AUTOIMMUNE_THYROID_DISEASE(MMU05320) | 50 | -0.40656 | -1.32746 | 0 | 0.91178 |
| APOPTOSIS_MULTIPLE_SPECIES(MMU04215) | 43 | -0.32676 | -1.32744 | 0 | 0.854462 |
| GRAFT_VERSUS_HOST_DISEASE(MMU05332) | 42 | -0.44022 | -1.30892 | 0 | 0.936538 |
| HEMATOPOIETIC_CELL_LINEAGE(MMU04640) | 99 | -0.40769 | -1.30514 | 0.127237 | 0.914646 |
| FOLATE_BIOSYNTHESIS(MMU00790) | 33 | -0.39012 | -1.30499 | 0 | 0.868255 |
| GLYCOSPHINGOLIPID_BIOSYNTHESIS_GLOBO_AND_ISOGLOBO_SERIES (MMU00603) | 16 | -0.48317 | -1.2731 | 0.084586 | 0.978683 |
| RIBOSOME(MMU03010) | 598 | -0.39708 | -1.27155 | 0 | 0.939766 |
| EPSTEIN_BARR_VIRUS_INFECTION(MMU05169) | 245 | -0.30413 | -1.26634 | 0.090909 | 0.91715 |
| INTESTINAL_IMMUNE_NETWORK_FOR_IGA_PRODUCTION(MMU04672) | 37 | -0.39237 | -1.26432 | 0 | 0.888771 |
| TYPE_I_DIABETES_MELLITUS(MMU04940) | 54 | -0.38967 | -1.25584 | 0 | 0.906172 |
| DRUG_METABOLISM_CYTOCHROME_P450(MMU00982) | 73 | -0.35353 | -1.25519 | 0 | 0.873372 |
| CYTOKINE_CYTOKINE_RECEPTOR_INTERACTION(MMU04060) | 243 | -0.35332 | -1.2514 | 0.077855 | 0.860194 |
| TASTE_TRANSDUCTION(MMU04742) | 57 | -0.41132 | -1.25123 | 0.187381 | 0.82911 |
| VITAMIN_DIGESTION_AND_ABSORPTION(MMU04977) | 26 | -0.41515 | -1.24695 | 0.109375 | 0.805756 |
| RNA_DEGRADATION(MMU03018) | 102 | -0.29429 | -1.24587 | 0.115702 | 0.778837 |
| CORONAVIRUS_DISEASE_COVID_19(MMU05171) | 676 | -0.36774 | -1.24262 | 0 | 0.769559 |
| PHENYLALANINE_METABOLISM(MMU00360) | 22 | -0.45174 | -1.24128 | 0.27553 | 0.74564 |
| NICOTINE_ADDICTION(MMU05033) | 41 | -0.42939 | -1.23759 | 0 | 0.731183 |

| NAME | SIZE | ES | NES | NOM p-value | FDR q-value |
|---|---|---|---|---|---|
| HERPES_SIMPLEX_VIRUS_1_INFECTION(MMU05168) | 426 | -0.24979 | -1.23472 | 0 | 0.716271 |
| MRNA_SURVEILLANCE_PATHWAY(MMU03015) | 133 | -0.26179 | -1.23393 | 0.097363 | 0.70117 |
| THERMOGENESIS(MMU04714) | 289 | -0.23778 | -1.23055 | 0.098291 | 0.694277 |
| ALLOGRAFT_REJECTION(MMU05330) | 42 | -0.38409 | -1.22947 | 0 | 0.678097 |
| CYTOSOLIC_DNA_SENSING_PATHWAY(MMU04623) | 78 | -0.33573 | -1.22866 | 0.118393 | 0.661811 |
| UBIQUITIN_MEDIATED_PROTEOLYSIS(MMU04120) | 219 | -0.24028 | -1.22461 | 0.111336 | 0.664101 |
| INFLUENZA_A(MMU05164) | 193 | -0.27392 | -1.19548 | 0.09375 | 0.769486 |
| VIRAL_LIFE_CYCLE_HIV_1(MMU03250) | 80 | -0.2663 | -1.18849 | 0.098121 | 0.781326 |
| NATURAL_KILLER_CELL_MEDIATED_CYTOTOXICITY(MMU04650) | 96 | -0.28871 | -1.18493 | 0.086705 | 0.777817 |
| FAT_DIGESTION_AND_ABSORPTION(MMU04975) | 44 | -0.36658 | -1.1759 | 0.199605 | 0.796448 |
| METABOLISM_OF_XENOBIOTICS_BY_CYTOCHROME_P450(MMU00980) | 75 | -0.33017 | -1.15245 | 0.127237 | 0.879044 |
| ASTHMA(MMU05310) | 20 | -0.39373 | -1.13991 | 0.272549 | 0.925266 |
| COCAINE_ADDICTION(MMU05030) | 50 | -0.34193 | -1.13902 | 0.185468 | 0.909015 |
| APOPTOSIS(MMU04210) | 177 | -0.2654 | -1.1359 | 0.085487 | 0.904272 |
| CIRCADIAN_RHYTHM(MMU04710) | 45 | -0.28643 | -1.12243 | 0.175908 | 0.936168 |
| SPLICEOSOME(MMU03040) | 188 | -0.25775 | -1.11925 | 0.370221 | 0.926378 |
| GLYCINE__SERINE_AND_THREONINE_METABOLISM(MMU00260) | 60 | -0.32101 | -1.11138 | 0.311321 | 0.94079 |
| PRION_DISEASE(MMU05020) | 375 | -0.24219 | -1.10778 | 0.190574 | 0.932185 |
| NOD_LIKE_RECEPTOR_SIGNALING_PATHWAY(MMU04621) | 199 | -0.24573 | -1.10634 | 0.296367 | 0.930562 |
| ALZHEIMER_DISEASE(MMU05010) | 494 | -0.21247 | -1.10361 | 0.262042 | 0.925509 |
| NEUTROPHIL_EXTRACELLULAR_TRAP_FORMATION(MMU04613) | 204 | -0.25271 | -1.09813 | 0.180873 | 0.934521 |
| CYSTEINE_AND_METHIONINE_METABOLISM(MMU00270) | 80 | -0.25227 | -1.09705 | 0.12549 | 0.919758 |
| RETINOL_METABOLISM(MMU00830) | 73 | -0.33687 | -1.08454 | 0.197895 | 0.953913 |
| CHEMICAL_CARCINOGENESIS_REACTIVE_OXYGEN_SPECIES(MMU05208) | 287 | -0.21359 | -1.08303 | 0.305328 | 0.939717 |
| ENDOCYTOSIS(MMU04144) | 315 | -0.22265 | -1.08249 | 0.298113 | 0.927871 |
| RETROGRADE_ENDOCANNABINOID_SIGNALING(MMU04723) | 168 | -0.25074 | -1.06581 | 0.29899 | 0.988429 |
| CARDIAC_MUSCLE_CONTRACTION(MMU04260) | 103 | -0.26929 | -1.05695 | 0.359841 | 1 |
| NON_ALCOHOLIC_FATTY_LIVER_DISEASE(MMU04932) | 197 | -0.2211 | -1.05555 | 0.290188 | 0.996239 |
| AFRICAN_TRYPANOSOMIASIS(MMU05143) | 27 | -0.34381 | -1.05124 | 0.416834 | 0.994031 |
| COLLECTING_DUCT_ACID_SECRETION(MMU04966) | 30 | -0.30167 | -1.04871 | 0.388235 | 0.986326 |
| IL_17_SIGNALING_PATHWAY(MMU04657) | 100 | -0.26912 | -1.04286 | 0.38833 | 0.990222 |
| ARGININE_BIOSYNTHESIS(MMU00220) | 26 | -0.35053 | -1.03446 | 0.206501 | 1 |

| NAME | SIZE | ES | NES | NOM p-value | FDR q-value |
|---|---|---|---|---|---|
| AMPHETAMINE_ADDICTION(MMU05031) | 77 | -0.24563 | -1.03362 | 0.41358 | 0.996599 |
| NEUROACTIVE_LIGAND_RECEPTOR_INTERACTION(MMU04080) | 323 | -0.2838 | -1.03217 | 0.28827 | 0.989836 |
| ATP_DEPENDENT_CHROMATIN_REMODELING(MMU03082) | 148 | -0.23474 | -1.02325 | 0.275654 | 1 |
| HUMAN_IMMUNODEFICIENCY_VIRUS_1_INFECTION(MMU05170) | 241 | -0.21381 | -1.02121 | 0.302041 | 1 |
| MELANOMA(MMU05218) | 80 | -0.24958 | -1.01244 | 0.396617 | 1 |
| HYPERTROPHIC_CARDIOMYOPATHY(MMU05410) | 114 | -0.26467 | -1.01226 | 0.38343 | 1 |
| CELL_ADHESION_MOLECULES(MMU04514) | 174 | -0.25129 | -1.01003 | 0.411321 | 1 |
| CELLULAR_SENESCENCE(MMU04218) | 211 | -0.22326 | -1.00923 | 0.298113 | 0.998207 |
| NECROPTOSIS(MMU04217) | 195 | -0.2134 | -1.00798 | 0.497018 | 0.990343 |
| STEROID_HORMONE_BIOSYNTHESIS(MMU00140) | 70 | -0.31053 | -1.00621 | 0.410506 | 0.984415 |
| POLYCOMB_REPRESSIVE_COMPLEX(MMU03083) | 104 | -0.23679 | -1.00511 | 0.622568 | 0.979655 |
| GLUTAMATERGIC_SYNAPSE(MMU04724) | 127 | -0.28566 | -1.00464 | 0.413208 | 0.96984 |
| PARKINSON_DISEASE(MMU05012) | 384 | -0.21157 | -0.99288 | 0.698718 | 1 |
| ARGININE_AND_PROLINE_METABOLISM(MMU00330) | 61 | -0.26418 | -0.99035 | 0.399602 | 0.996229 |
| STAPHYLOCOCCUS_AUREUS_INFECTION(MMU05150) | 76 | -0.28786 | -0.98983 | 0.379921 | 0.984513 |
| PENTOSE_AND_GLUCURONATE_INTERCONVERSIONS(MMU00040) | 38 | -0.30284 | -0.9891 | 0.360656 | 0.97448 |
| ARACHIDONIC_ACID_METABOLISM(MMU00590) | 76 | -0.25742 | -0.98406 | 0.466926 | 0.978369 |
| OXIDATIVE_PHOSPHORYLATION(MMU00190) | 182 | -0.24431 | -0.98121 | 0.48218 | 0.975143 |
| RAS_SIGNALING_PATHWAY(MMU04014) | 251 | -0.2211 | -0.97747 | 0.318868 | 0.975731 |
| PEROXISOME(MMU04146) | 102 | -0.23213 | -0.97486 | 0.508876 | 0.976328 |
| ARRHYTHMOGENIC_RIGHT_VENTRICULAR_CARDIOMYOPATHY(MMU05412) | 101 | -0.25783 | -0.96936 | 0.510597 | 0.986688 |
| MATURITY_ONSET_DIABETES_OF_THE_YOUNG(MMU04950) | 25 | -0.31568 | -0.96933 | 0.481405 | 0.975692 |
| T_CELL_RECEPTOR_SIGNALING_PATHWAY(MMU04660) | 128 | -0.18426 | -0.96766 | 0.499044 | 0.968366 |
| FLUID_SHEAR_STRESS_AND_ATHEROSCLEROSIS(MMU05418) | 187 | -0.2067 | -0.96737 | 0.466038 | 0.95829 |
| BLADDER_CANCER(MMU05219) | 47 | -0.27267 | -0.96461 | 0.506692 | 0.959011 |
| VIRAL_PROTEIN_INTERACTION_WITH_CYTOKINE_AND_CYTOKINE_RECEPTOR(MMU04061) | 74 | -0.29665 | -0.96172 | 0.469903 | 0.96045 |
| ALPHA_LINOLENIC_ACID_METABOLISM(MMU00592) | 27 | -0.2813 | -0.95966 | 0.424419 | 0.956232 |
| BILE_SECRETION(MMU04976) | 95 | -0.27902 | -0.95788 | 0.3859 | 0.948096 |
| PANCREATIC_SECRETION(MMU04972) | 111 | -0.28039 | -0.95693 | 0.584493 | 0.940755 |
| SPINOCEREBELLAR_ATAXIA(MMU05017) | 186 | -0.21045 | -0.95553 | 0.368821 | 0.936079 |
| LEUKOCYTE_TRANSENDOTHELIAL_MIGRATION(MMU04670) | 136 | -0.22738 | -0.94961 | 0.528517 | 0.945374 |
| LIPID_AND_ATHEROSCLEROSIS(MMU05417) | 233 | -0.19137 | -0.94821 | 0.539063 | 0.939981 |

| NAME | SIZE | ES | NES | NOM p-value | FDR q-value |
|---|---|---|---|---|---|
| REGULATION_OF_ACTIN_CYTOSKELETON(MMU04810) | 271 | -0.18826 | -0.9397 | 0.509434 | 0.959726 |
| ANTIGEN_PROCESSING_AND_PRESENTATION(MMU04612) | 89 | -0.27066 | -0.93821 | 0.395112 | 0.955978 |
| PD_L1_EXPRESSION_AND_PD_1_CHECKPOINT_PATHWAY_IN_CANCER(MMU05235) | 98 | -0.20102 | -0.92956 | 0.717899 | 0.977553 |
| MEASLES(MMU05162) | 154 | -0.20849 | -0.92197 | 0.706478 | 0.990829 |
| JAK_STAT_SIGNALING_PATHWAY(MMU04630) | 152 | -0.25759 | -0.91968 | 0.506692 | 0.991033 |
| GLYCEROLIPID_METABOLISM(MMU00561) | 73 | -0.24348 | -0.91535 | 0.611321 | 0.991963 |
| DILATED_CARDIOMYOPATHY(MMU05414) | 116 | -0.25222 | -0.90462 | 0.807322 | 1 |
| TH17_CELL_DIFFERENTIATION(MMU04659) | 122 | -0.20683 | -0.90322 | 0.801619 | 1 |
| AMYOTROPHIC_LATERAL_SCLEROSIS(MMU05014) | 544 | -0.17074 | -0.90294 | 0.471545 | 0.995942 |
| LINOLEIC_ACID_METABOLISM(MMU00591) | 34 | -0.25413 | -0.89532 | 0.605932 | 1 |
| CHEMICAL_CARCINOGENESIS_DNA_ADDUCTS(MMU05204) | 66 | -0.26672 | -0.892 | 0.809426 | 1 |
| VIRAL_MYOCARDITIS(MMU05416) | 97 | -0.2418 | -0.89088 | 0.786561 | 0.997932 |
| TAURINE_AND_HYPOTAURINE_METABOLISM(MMU00430) | 20 | -0.35416 | -0.88989 | 0.606589 | 0.995686 |
| INFLAMMATORY_BOWEL_DISEASE(MMU05321) | 59 | -0.24506 | -0.88696 | 0.512295 | 0.993124 |
| BASAL_TRANSCRIPTION_FACTORS(MMU03022) | 54 | -0.23926 | -0.88472 | 0.605372 | 0.990175 |
| FANCONI_ANEMIA_PATHWAY(MMU03460) | 55 | -0.20766 | -0.87764 | 0.61657 | 1 |
| RHEUMATOID_ARTHRITIS(MMU05323) | 81 | -0.24279 | -0.87083 | 0.805882 | 1 |
| KAPOSI_SARCOMA_ASSOCIATED_HERPESVIRUS_INFECTION(MMU05167) | 236 | -0.20004 | -0.86659 | 0.898833 | 1 |
| ESTROGEN_SIGNALING_PATHWAY(MMU04915) | 154 | -0.2167 | -0.86609 | 0.622351 | 1 |
| YERSINIA_INFECTION(MMU05135) | 156 | -0.17211 | -0.86572 | 0.604207 | 0.9994 |
| OSTEOCLAST_DIFFERENTIATION(MMU04380) | 131 | -0.20941 | -0.86376 | 0.807393 | 0.993927 |
| C_TYPE_LECTIN_RECEPTOR_SIGNALING_PATHWAY(MMU04625) | 115 | -0.19301 | -0.86006 | 0.706226 | 0.996253 |
| GLYCOSAMINOGLYCAN_BIOSYNTHESIS_HEPARAN_SULFATE_HEPARIN(MMU00534) | 25 | -0.31146 | -0.85478 | 0.60566 | 1 |
| DOPAMINERGIC_SYNAPSE(MMU04728) | 149 | -0.21315 | -0.85448 | 0.813726 | 0.994294 |
| CHEMICAL_CARCINOGENESIS_RECEPTOR_ACTIVATION(MMU05207) | 226 | -0.20097 | -0.85065 | 0.803922 | 0.993892 |
| PHAGOSOME(MMU04145) | 203 | -0.2018 | -0.84561 | 0.788235 | 0.997738 |
| MAPK_SIGNALING_PATHWAY(MMU04010) | 324 | -0.18719 | -0.84156 | 0.590566 | 0.998588 |
| LEGIONELLOSIS(MMU05134) | 84 | -0.23979 | -0.84109 | 0.890985 | 0.991802 |
| THIAMINE_METABOLISM(MMU00730) | 18 | -0.28163 | -0.83701 | 0.805882 | 0.995312 |
| ANTIFOLATE_RESISTANCE(MMU01523) | 28 | -0.2609 | -0.83699 | 0.5 | 0.987766 |
| ECM_RECEPTOR_INTERACTION(MMU04512) | 97 | -0.27011 | -0.83345 | 0.541109 | 0.988328 |

| NAME | SIZE | ES | NES | NOM p-value | FDR q-value |
|---|---|---|---|---|---|
| PI3K_AKT_SIGNALING_PATHWAY(MMU04151) | 387 | -0.20863 | -0.82666 | 0.781132 | 0.995301 |
| BIOSYNTHESIS_OF_COFACTORS(MMU01240) | 166 | -0.16605 | -0.82635 | 0.900574 | 0.988474 |
| HUNTINGTON_DISEASE(MMU05016) | 459 | -0.17236 | -0.82192 | 0.805846 | 0.991471 |
| LEISHMANIASIS(MMU05140) | 80 | -0.20364 | -0.81556 | 0.802444 | 0.996614 |
| PATHWAYS_OF_NEURODEGENERATION_MULTIPLE_DISEASES(MMU05022) | 654 | -0.16469 | -0.81391 | 0.796078 | 0.992316 |
| AGE_RAGE_SIGNALING_PATHWAY_IN_DIABETIC_COMPLICATIONS(MMU04933) | 108 | -0.23131 | -0.81238 | 0.533074 | 0.985995 |
| ALCOHOLISM(MMU05034) | 189 | -0.19047 | -0.80869 | 0.89495 | 0.98314 |
| GLYCOLYSIS_GLUCONEOGENESIS(MMU00010) | 97 | -0.1994 | -0.80539 | 0.8 | 0.981243 |
| NUCLEOTIDE_EXCISION_REPAIR(MMU03420) | 74 | -0.19295 | -0.80426 | 0.793684 | 0.978357 |
| PLATELET_ACTIVATION(MMU04611) | 148 | -0.18256 | -0.80041 | 0.813688 | 0.98096 |
| ALCOHOLIC_LIVER_DISEASE(MMU04936) | 136 | -0.20996 | -0.7992 | 0.805447 | 0.977319 |
| VEGF_SIGNALING_PATHWAY(MMU04370) | 66 | -0.19059 | -0.79646 | 0.89662 | 0.975106 |
| RAP1_SIGNALING_PATHWAY(MMU04015) | 240 | -0.17975 | -0.79079 | 0.803774 | 0.978265 |
| HUMAN_PAPILLOMAVIRUS_INFECTION(MMU05165) | 370 | -0.20802 | -0.7885 | 0.697719 | 0.973958 |
| PHOTOTRANSDUCTION(MMU04744) | 27 | -0.28786 | -0.78393 | 0.661157 | 0.974212 |
| CALCIUM_SIGNALING_PATHWAY(MMU04020) | 276 | -0.20396 | -0.78236 | 0.697719 | 0.969606 |
| ASCORBATE_AND_ALDARATE_METABOLISM(MMU00053) | 26 | -0.2722 | -0.77864 | 0.824253 | 0.967391 |
| HIPPO_SIGNALING_PATHWAY(MMU04390) | 189 | -0.18965 | -0.77596 | 0.813208 | 0.967876 |
| CHAGAS_DISEASE(MMU05142) | 101 | -0.19307 | -0.77373 | 0.712245 | 0.964583 |
| FATTY_ACID_DEGRADATION(MMU00071) | 59 | -0.23616 | -0.77138 | 0.707457 | 0.964105 |
| HOMOLOGOUS_RECOMBINATION(MMU03440) | 45 | -0.16233 | -0.76911 | 0.811359 | 0.961485 |
| RIG_I_LIKE_RECEPTOR_SIGNALING_PATHWAY(MMU04622) | 61 | -0.18419 | -0.76499 | 0.902256 | 0.960493 |
| HUMAN_T_CELL_LEUKEMIA_VIRUS_1_INFECTION(MMU05166) | 259 | -0.18262 | -0.76158 | 0.813726 | 0.9593 |
| INSULIN_RESISTANCE(MMU04931) | 121 | -0.22123 | -0.76081 | 0.523901 | 0.953577 |
| TH1_AND_TH2_CELL_DIFFERENTIATION(MMU04658) | 89 | -0.19601 | -0.75831 | 0.89662 | 0.951091 |
| HUMAN_CYTOMEGALOVIRUS_INFECTION(MMU05163) | 250 | -0.18651 | -0.74965 | 0.807393 | 0.958797 |
| MORPHINE_ADDICTION(MMU05032) | 95 | -0.19743 | -0.74928 | 0.898039 | 0.954182 |
| OXYTOCIN_SIGNALING_PATHWAY(MMU04921) | 183 | -0.18003 | -0.74747 | 0.898039 | 0.949015 |
| RELAXIN_SIGNALING_PATHWAY(MMU04926) | 133 | -0.22068 | -0.70648 | 0.717899 | 0.985609 |
| FOCAL_ADHESION(MMU04510) | 235 | -0.18197 | -0.69159 | 0.807393 | 0.999675 |
| REGULATION_OF_LIPOLYSIS_IN_ADIPOCYTES(MMU04923) | 60 | -0.23933 | -0.68729 | 0.803922 | 0.997373 |
| GLYCEROPHOSPHOLIPID_METABOLISM(MMU00564) | 109 | -0.17464 | -0.68297 | 0.71869 | 0.995884 |

| NAME | SIZE | ES | NES | NOM p-value | FDR q-value |
|---|---|---|---|---|---|
| ADRENERGIC_SIGNALING_IN_CARDIOMYOCYTES(MMU04261) | 165 | -0.1756 | -0.67639 | 0.898833 | 0.994477 |
| SEROTONERGIC_SYNAPSE(MMU04726) | 123 | -0.1853 | -0.67054 | 0.898039 | 0.992281 |
| WNT_SIGNALING_PATHWAY(MMU04310) | 202 | -0.17896 | -0.66815 | 0.898039 | 0.988394 |
| 2_OXOCARBOXYLIC_ACID_METABOLISM(MMU01210) | 41 | -0.16816 | -0.66699 | 0.780303 | 0.984078 |
| GALACTOSE_METABOLISM(MMU00052) | 36 | -0.16562 | -0.66465 | 0.902256 | 0.980094 |
| GNRH_SECRETION(MMU04929) | 70 | -0.21164 | -0.66438 | 0.813726 | 0.974435 |
| BASE_EXCISION_REPAIR(MMU03410) | 48 | -0.16378 | -0.65825 | 0.790745 | 0.971764 |
| GABAERGIC_SYNAPSE(MMU04727) | 96 | -0.17133 | -0.65012 | 0.898833 | 0.969993 |
| GLYCOSPHINGOLIPID_BIOSYNTHESIS_LACTO_AND_NEOLACTO_SERIES (MMU00601) | 26 | -0.21564 | -0.64291 | 0.901887 | 0.971171 |
| STARCH_AND_SUCROSE_METABOLISM(MMU00500) | 32 | -0.17701 | -0.63583 | 0.899225 | 0.970655 |
| AMOEBIASIS(MMU05146) | 99 | -0.20958 | -0.63074 | 0.813208 | 0.968429 |
| TOLL_LIKE_RECEPTOR_SIGNALING_PATHWAY(MMU04620) | 92 | -0.16227 | -0.60523 | 0.898833 | 0.978005 |
| BASAL_CELL_CARCINOMA(MMU05217) | 68 | -0.19034 | -0.57233 | 0.819392 | 0.98689 |
| TYPE_II_DIABETES_MELLITUS(MMU04930) | 53 | -0.20524 | -0.55483 | 0.790196 | 0.986986 |
| LYSINE_DEGRADATION(MMU00310) | 68 | -0.13256 | -0.49114 | 0.89662 | 0.994731 |
