# Supplementary material for: Possible Role of Gut Microbiota in Polypropylene Microplastics-Induced Immunity and Reproductive Dysfunction in Mice
Source: Toxics. 2026 Jul 31;14(8):679. doi: 10.3390/toxics14080679 (PMC13517389; doi:10.3390/toxics14080679)
Supplement: Supplementary file 1 [file toxics-14-00679-s001.zip › Supplementary Information S10.pdf]

Supplementar Information S10. The *P* values of the Wilcoxon Rank-Sum test for the Alpha diversity index between the PP and the control groups, as well as between males and females.

| Group | Chao 1 | Goods coverage | Observed features | Pielou_e | Shannon | Simpson |
|---|---|---|---|---|---|---|
| Control vs PP | 0.46 | 0.10 | 0.56 | 0.50 | 0.70 | 0.53 |
| Female vs Male | 0.18 | 0.40 | 0.16 | 0.96 | 0.61 | 0.68 |
