# Supplementary material for: Possible Role of Gut Microbiota in Polypropylene Microplastics-Induced Immunity and Reproductive Dysfunction in Mice
Source: Toxics. 2026 Jul 31;14(8):679. doi: 10.3390/toxics14080679 (PMC13517389; doi:10.3390/toxics14080679)
Supplement: Supplementary file 1 [file toxics-14-00679-s001.zip › Supplementary Information S4.pdf]

Supplementary Information S4. GSEA of enriched GO Terms in the control group.

| NAME | SIZE | ES | NES | NOM p-value | FDR q-value |
|---|---|---|---|---|---|
| REGULATION_OF_HYDROLASE_ACTIVITY(GO:0051336) | 16 | -0.44414 | -1.54304 | 0 | 1 |
| PHEROMONE_RECEPTOR_ACTIVITY(GO:0016503) | 30 | -0.64631 | -1.48741 | 0 | 1 |
| REGULATION_OF_PROGRAMMED_CELL_DEATH(GO:0043067) | 35 | -0.45249 | -1.47879 | 0.096774 | 1 |
| REGULATION_OF_CELL_DEATH(GO:0010941) | 35 | -0.45249 | -1.47879 | 0.096774 | 1 |
| REGULATION_OF_APOPTOTIC_PROCESS(GO:0042981) | 35 | -0.45249 | -1.47879 | 0.096774 | 1 |
| CELL_DEATH(GO:0008219) | 44 | -0.40564 | -1.4736 | 0 | 1 |
| APOPTOTIC_PROCESS(GO:0006915) | 44 | -0.40564 | -1.4736 | 0 | 1 |
| PROGRAMMED_CELL_DEATH(GO:0012501) | 44 | -0.40564 | -1.4736 | 0 | 1 |
| CARGO_RECEPTOR_ACTIVITY(GO:0038024) | 28 | -0.52745 | -1.40765 | 0 | 1 |
| SCAVENGER_RECEPTOR_ACTIVITY(GO:0005044) | 28 | -0.52745 | -1.40765 | 0 | 1 |
| OLFACTORY_RECEPTOR_ACTIVITY(GO:0004984) | 236 | -0.47608 | -1.37077 | 0 | 1 |
| SERINE_TYPE_ENDOPEPTIDASE_INHIBITOR_ACTIVITY(GO:0004867) | 18 | -0.48874 | -1.36849 | 0 | 1 |
| EXTRACELLULAR_REGION_PART(GO:0044421) | 42 | -0.47446 | -1.35603 | 0.094303 | 1 |
| SERINE_TYPE_ENDOPEPTIDASE_ACTIVITY(GO:0004252) | 120 | -0.36347 | -1.35291 | 0 | 1 |
| G_PROTEIN_COUPLED_RECEPTOR_SIGNALING_PATHWAY(GO:0007186) | 535 | -0.40829 | -1.3521 | 0 | 1 |
| SUPRAMOLECULAR_FIBER(GO:0099512) | 20 | -0.57363 | -1.35111 | 0.09505 | 1 |
| SUPRAMOLECULAR_COMPLEX(GO:0099080) | 20 | -0.57363 | -1.35111 | 0.09505 | 1 |
| SUPRAMOLECULAR_POLYMER(GO:0099081) | 20 | -0.57363 | -1.35111 | 0.09505 | 1 |
| METAL_CLUSTER_BINDING(GO:0051540) | 23 | -0.33763 | -1.35047 | 0.102713 | 1 |
| IRON_SULFUR_CLUSTER_BINDING(GO:0051536) | 23 | -0.33763 | -1.35047 | 0.102713 | 1 |
| SIGNALING_RECEPTOR_ACTIVITY(GO:0038023) | 532 | -0.40225 | -1.34375 | 0 | 1 |

| NAME | SIZE | ES | NES | NOM p-value | FDR q-value |
|---|---|---|---|---|---|
| MOLECULAR_TRANSDUCER_ACTIVITY(GO:0060089) | 532 | -0.40225 | -1.34375 | 0 | 0.972866 |
| EXTRACELLULAR_REGION(GO:0005576) | 199 | -0.36321 | -1.34348 | 0 | 0.937361 |
| TRANSMEMBRANE_SIGNALING_RECEPTOR_ACTIVITY(GO:0004888) | 525 | -0.40648 | -1.34305 | 0 | 0.904037 |
| SIGNAL_TRANSDUCER_ACTIVITY(GO:0004871) | 536 | -0.39975 | -1.34031 | 0 | 0.87542 |
| INTEGRAL_COMPONENT_OF_MEMBRANE(GO:0016021) | 997 | -0.34417 | -1.33619 | 0 | 0.86807 |
| INTRINSIC_COMPONENT_OF_MEMBRANE(GO:0031224) | 999 | -0.34314 | -1.33545 | 0 | 0.845209 |
| RECEPTOR_REGULATOR_ACTIVITY(GO:0030545) | 109 | -0.41548 | -1.33488 | 0 | 0.821511 |
| RECEPTOR_LIGAND_ACTIVITY(GO:0048018) | 109 | -0.41548 | -1.33488 | 0 | 0.793183 |
| MEMBRANE_PART(GO:0044425) | 1072 | -0.32642 | -1.32766 | 0 | 0.813767 |
| SODIUM_ION_TRANSMEMBRANE_TRANSPORTER_ACTIVITY(GO:0015081) | 21 | -0.51369 | -1.32365 | 0 | 0.807177 |
| GROWTH_FACTOR_ACTIVITY(GO:0008083) | 45 | -0.41672 | -1.31747 | 0.097959 | 0.804801 |
| LIPOPROTEIN_METABOLIC_PROCESS(GO:0042157) | 19 | -0.42369 | -1.30518 | 0.097276 | 0.836104 |
| ANION_TRANSPORT(GO:0006820) | 41 | -0.43068 | -1.30512 | 0.185185 | 0.813072 |
| HORMONE_ACTIVITY(GO:0005179) | 36 | -0.47651 | -1.29764 | 0.098361 | 0.829918 |
| SERINE_TYPE_PEPTIDASE_ACTIVITY(GO:0008236) | 129 | -0.34046 | -1.29351 | 0 | 0.835979 |
| SERINE_HYDROLASE_ACTIVITY(GO:0017171) | 129 | -0.34046 | -1.29351 | 0 | 0.813385 |
| PROTEIN_DEPHOSPHORYLATION(GO:0006470) | 31 | -0.3234 | -1.29026 | 0 | 0.801579 |
| ENDOPEPTIDASE_ACTIVITY(GO:0004175) | 229 | -0.28471 | -1.27849 | 0 | 0.837866 |
| SIGNALING_RECEPTOR_BINDING(GO:0005102) | 163 | -0.38825 | -1.27437 | 0 | 0.848114 |
| ANION_TRANSMEMBRANE_TRANSPORTER_ACTIVITY(GO:0008509) | 25 | -0.48168 | -1.26757 | 0.305051 | 0.847517 |
| EXTRACELLULAR_SPACE(GO:0005615) | 30 | -0.49186 | -1.26209 | 0.106299 | 0.854802 |
| REGULATION_OF_MOLECULAR_FUNCTION(GO:0065009) | 21 | -0.34503 | -1.25477 | 0.203884 | 0.869449 |

| NAME | SIZE | ES | NES | NOM p-value | FDR q-value |
| --- | --- | --- | --- | --- | --- |
| REGULATION_OF_CATALYTIC_ACTIVITY(GO:0050790) | 21 | -0.34503 | -1.25477 | 0.203884 | 0.849689 |
| G_PROTEIN_COUPLED_RECEPTOR_ACTIVITY(GO:0004930) | 265 | -0.35407 | -1.2469 | 0 | 0.871321 |
| SYMPORTER_ACTIVITY(GO:0015293) | 20 | -0.49616 | -1.24398 | 0.291242 | 0.870429 |
| SECONDARY_ACTIVE_TRANSMEMBRANE_TRANSPORTER_ACTIVITY(GO:0015291) | 32 | -0.40189 | -1.24049 | 0.184 | 0.868589 |
| MEMBRANE(GO:0016020) | 1606 | -0.29858 | -1.22369 | 0 | 0.897361 |
| RESPONSE_TO_ORGANIC_SUBSTANCE(GO:0010033) | 18 | -0.46755 | -1.22131 | 0.112676 | 0.89314 |
| CELLULAR_COMPONENT(GO:0005575) | 2950 | -0.24229 | -1.20842 | 0 | 0.915961 |
| IMMUNE_RESPONSE(GO:0006955) | 57 | -0.38262 | -1.20446 | 0.181452 | 0.913858 |
| DNA_METABOLIC_PROCESS(GO:0006259) | 93 | -0.28192 | -1.20147 | 0.221782 | 0.911368 |
| PYRIDOXAL_PHOSPHATE_BINDING(GO:0030170) | 24 | -0.4337 | -1.19908 | 0.207767 | 0.907037 |
| VITAMIN_B6_BINDING(GO:0070279) | 24 | -0.4337 | -1.19908 | 0.207767 | 0.89024 |
| SIGNALING(GO:0023052) | 903 | -0.29991 | -1.19489 | 0 | 0.892332 |
| SIGNAL_TRANSDUCTION(GO:0007165) | 896 | -0.29797 | -1.19482 | 0 | 0.878492 |
| IMMUNE_SYSTEM_PROCESS(GO:0002376) | 60 | -0.37734 | -1.18957 | 0.181452 | 0.885247 |
| CELL_COMMUNICATION(GO:0007154) | 906 | -0.29855 | -1.18769 | 0 | 0.878338 |
| CELLULAR_RESPONSE_TO_STIMULUS(GO:0051716) | 949 | -0.28993 | -1.18411 | 0 | 0.872849 |
| RESPONSE_TO_STIMULUS(GO:0050896) | 1036 | -0.28685 | -1.17913 | 0 | 0.880931 |
| CYTOKINE_RECEPTOR_BINDING(GO:0005126) | 34 | -0.42426 | -1.16778 | 0.201629 | 0.91646 |
| RIBONUCLEASE_ACTIVITY(GO:0004540) | 17 | -0.35215 | -1.16298 | 0.392354 | 0.918435 |
| CATION_CHANNEL_ACTIVITY(GO:0005261) | 32 | -0.3131 | -1.15074 | 0.2 | 0.947301 |
| MOLECULAR_FUNCTION_REGULATOR(GO:0098772) | 298 | -0.26363 | -1.15073 | 0.096579 | 0.933328 |
| RESPONSE_TO_CHEMICAL(GO:0042221) | 28 | -0.37536 | -1.14366 | 0.200382 | 0.938914 |
| INTRAMOLECULAR_TRANSFERASE_ACTIVITY(GO:0016866) | 18 | -0.3607 | -1.14069 | 0.202479 | 0.940532 |

| NAME | SIZE | ES | NES | NOM p-value | FDR q-value |
|---|---|---|---|---|---|
| SECRETION(GO:0046903) | 23 | -0.33129 | -1.1241 | 0.292776 | 1 |
| DEPHOSPHORYLATION(GO:0016311) | 60 | -0.21883 | -1.12124 | 0.091603 | 1 |
| INORGANIC_ANION_TRANSPORT(GO:0015698) | 18 | -0.38896 | -1.10748 | 0.27767 | 1 |
| CYTOKINE_ACTIVITY(GO:0005125) | 28 | -0.43135 | -1.10745 | 0.190574 | 1 |
| CELL_CELL_ADHESION(GO:0098609) | 80 | -0.35326 | -1.09732 | 0.093023 | 1 |
| HOMOPHILIC_CELL_ADHESION_VIA_PLASMA_MEMBRANE_ ADHESION_MOLECULES(GO:0007156) | 80 | -0.35326 | -1.09732 | 0.093023 | 1 |
| CELL_CELL_ADHESION_VIA_PLASMA_MEMBRANE_ADHESIO N_MOLECULES(GO:0098742) | 80 | -0.35326 | -1.09732 | 0.093023 | 1 |
| REGULATION_OF_BIOLOGICAL_PROCESS(GO:0050789) | 1525 | -0.23694 | -1.09719 | 0 | 1 |
| BIOLOGICAL_REGULATION(GO:0065007) | 1551 | -0.23675 | -1.09649 | 0 | 1 |
| METAL_ION_TRANSMEMBRANE_TRANSPORTER_ACTIVITY(G O:0046873) | 64 | -0.33421 | -1.09349 | 0.307536 | 1 |
| LIPID_TRANSPORT(GO:0006869) | 29 | -0.3853 | -1.09022 | 0.203156 | 1 |
| REGULATION_OF_CELLULAR_PROCESS(GO:0050794) | 1505 | -0.23668 | -1.08805 | 0 | 1 |
| POLYMERIC_CYTOSKELETAL_FIBER(GO:0099513) | 15 | -0.48809 | -1.086 | 0.284333 | 1 |
| RNA_MODIFICATION(GO:0009451) | 24 | -0.27883 | -1.07836 | 0.366667 | 1 |
| PROTEASOME_COMPLEX(GO:0000502) | 24 | -0.37569 | -1.07732 | 0.392079 | 1 |
| ENDOPEPTIDASE_COMPLEX(GO:1905369) | 24 | -0.37569 | -1.07732 | 0.392079 | 1 |
| LIGAND_GATED_ION_CHANNEL_ACTIVITY(GO:0015276) | 43 | -0.35764 | -1.07672 | 0.27459 | 1 |
| LIGAND_GATED_CHANNEL_ACTIVITY(GO:0022834) | 43 | -0.35764 | -1.07672 | 0.27459 | 0.998588 |
| EXTRACELLULAR_LIGAND_GATED_ION_CHANNEL_ACTIVIT Y(GO:0005230) | 41 | -0.35932 | -1.07629 | 0.27459 | 0.988283 |
| ENDOPLASMIC_RETICULUM_SUBCOMPARTMENT(GO:0098827 ) | 27 | -0.26316 | -1.07111 | 0.394175 | 0.996427 |
| ENDOPLASMIC_RETICULUM_MEMBRANE(GO:0005789) | 27 | -0.26316 | -1.07111 | 0.394175 | 0.984974 |
| ENDOPLASMIC_RETICULUM_PART(GO:0044432) | 28 | -0.26003 | -1.07071 | 0.394175 | 0.9751 |

| NAME | SIZE | ES | NES | NOM p-value | FDR q-value |
|---|---|---|---|---|---|
| LIPID_LOCALIZATION(GO:0010876) | 30 | -0.37212 | -1.06963 | 0.203156 | 0.972308 |
| INORGANIC_ANION_TRANSMEMBRANE_TRANSPORTER_ACTIVITY(GO:0015103) | 15 | -0.4158 | -1.06875 | 0.286573 | 0.967018 |
| ORGANELLE_SUBCOMPARTMENT(GO:0031984) | 40 | -0.23823 | -1.05504 | 0.20381 | 1 |
| TRANSMEMBRANE_TRANSPORTER_COMPLEX(GO:1902495) | 17 | -0.39694 | -1.0424 | 0.503018 | 1 |
| TRANSPORTER_COMPLEX(GO:1990351) | 17 | -0.39694 | -1.0424 | 0.503018 | 1 |
| CATALYTIC_ACTIVITY__ACTING_ON_DNA(GO:0140097) | 30 | -0.26102 | -1.03863 | 0.507014 | 1 |
| PROTEASOME_CORE_COMPLEX(GO:0005839) | 23 | -0.38018 | -1.03392 | 0.392079 | 1 |
| THREONINE_TYPE_PEPTIDASE_ACTIVITY(GO:0070003) | 23 | -0.38018 | -1.03392 | 0.392079 | 1 |
| THREONINE_TYPE_ENDOPEPTIDASE_ACTIVITY(GO:0004298) | 23 | -0.38018 | -1.03392 | 0.392079 | 1 |
| NUCLEAR_OUTER_MEMBRANE_ENDOPLASMIC_RETICULUM_MEMBRANE_NETWORK(GO:0042175) | 28 | -0.24147 | -1.02892 | 0.6 | 1 |
| VITAMIN_BINDING(GO:0019842) | 31 | -0.33061 | -1.02845 | 0.4 | 1 |
| PHOSPHOLIPASE_ACTIVITY(GO:0004620) | 21 | -0.35272 | -1.02493 | 0.385659 | 1 |
| ACTIN_CYTOSKELETON(GO:0015629) | 44 | -0.28454 | -1.02045 | 0.39961 | 1 |
| CARBOXYLIC_ESTER_HYDROLASE_ACTIVITY(GO:0052689) | 21 | -0.32017 | -1.01694 | 0.415058 | 1 |
| PROTEIN_TYROSINE_PHOSPHATASE_ACTIVITY(GO:0004725) | 27 | -0.26508 | -1.015 | 0.610778 | 1 |
| REGULATION_OF_CELLULAR_PROTEIN_METABOLIC_PROCESS(GO:0032268) | 18 | -0.33214 | -1.00954 | 0.404255 | 1 |
| REGULATION_OF_PROTEIN_METABOLIC_PROCESS(GO:0051246) | 18 | -0.33214 | -1.00954 | 0.404255 | 1 |
| ORGANONITROGEN_COMPOUND_CATABOLIC_PROCESS(GO:1901565) | 58 | -0.28848 | -1.00435 | 0.591085 | 1 |
| PROTEIN_CONTAINING_COMPLEX_BINDING(GO:0044877) | 33 | -0.29592 | -1.00323 | 0.342685 | 1 |
| MICROTUBULE_CYTOSKELETON_ORGANIZATION(GO:0000226) | 18 | -0.30827 | -0.99951 | 0.385567 | 1 |
| HYDROLASE_ACTIVITY__HYDROLYZING_O_GLYCOSYL_COMPOUNDS(GO:0004553) | 36 | -0.31424 | -0.9994 | 0.614173 | 1 |

| NAME | SIZE | ES | NES | NOM p-value | FDR q-value |
|---|---|---|---|---|---|
| ORGANIC_ANION_TRANSPORT(GO:0015711) | 16 | -0.38623 | -0.99151 | 0.484556 | 1 |
| ION_TRANSMEMBRANE_TRANSPORTER_ACTIVITY(GO:0015075) | 238 | -0.26801 | -0.99124 | 0.303823 | 1 |
| DEFENSE_RESPONSE(GO:0006952) | 16 | -0.4883 | -0.98866 | 0.52449 | 1 |
| LIPASE_ACTIVITY(GO:0016298) | 22 | -0.33082 | -0.98779 | 0.379771 | 1 |
| ION_TRANSPORT(GO:0006811) | 251 | -0.26025 | -0.98164 | 0.303823 | 1 |
| ACTIVE_TRANSMEMBRANE_TRANSPORTER_ACTIVITY(GO:0022804) | 59 | -0.30195 | -0.98124 | 0.3861 | 1 |
| ION_CHANNEL_ACTIVITY(GO:0005216) | 130 | -0.28543 | -0.97309 | 0.517312 | 1 |
| SUBSTRATE_SPECIFIC_CHANNEL_ACTIVITY(GO:0022838) | 130 | -0.28543 | -0.97309 | 0.517312 | 1 |
| MULTI_ORGANISM_PROCESS(GO:0051704) | 24 | -0.32543 | -0.97069 | 0.391566 | 1 |
| COFACTOR_BINDING(GO:0048037) | 199 | -0.22761 | -0.96996 | 0.480885 | 1 |
| PHOSPHATASE_ACTIVITY(GO:0016791) | 69 | -0.21604 | -0.9686 | 0.694657 | 1 |
| PEPTIDASE_ACTIVITY__ACTING_ON_L_AMINO_ACID_PEPTIDES(GO:0070011) | 339 | -0.20553 | -0.96692 | 0.612205 | 1 |
| TRANSMEMBRANE_TRANSPORTER_ACTIVITY(GO:0022857) | 341 | -0.26697 | -0.96475 | 0.303823 | 1 |
| AROMATIC_COMPOUND_CATABOLIC_PROCESS(GO:0019439) | 37 | -0.28683 | -0.96322 | 0.715953 | 1 |
| MONOVALENT_INORGANIC_CATION_TRANSMEMBRANE_TRANSPORTER_ACTIVITY(GO:0015077) | 70 | -0.23941 | -0.96296 | 0.509542 | 1 |
| TRANSPORTER_ACTIVITY(GO:0005215) | 380 | -0.26068 | -0.96086 | 0.404427 | 0.999487 |
| ORGANIC_CYCLIC_COMPOUND_CATABOLIC_PROCESS(GO:1901361) | 38 | -0.28041 | -0.95432 | 0.715953 | 1 |
| PASSIVE_TRANSMEMBRANE_TRANSPORTER_ACTIVITY(GO:0022803) | 138 | -0.27762 | -0.95394 | 0.404427 | 1 |
| CHANNEL_ACTIVITY(GO:0015267) | 138 | -0.27762 | -0.95394 | 0.404427 | 0.997909 |
| INTRINSIC_COMPONENT_OF_PLASMA_MEMBRANE(GO:0031226) | 16 | -0.36057 | -0.95224 | 0.387597 | 0.995364 |
| PEPTIDASE_ACTIVITY(GO:0008233) | 349 | -0.20437 | -0.95019 | 0.698819 | 0.993233 |

| NAME | SIZE | ES | NES | NOM p-value | FDR q-value |
|---|---|---|---|---|---|
| CELLULAR_PROCESS(GO:0009987) | 3068 | -0.16618 | -0.94585 | 0.793893 | 0.994632 |
| STRUCTURAL_MOLECULE_ACTIVITY(GO:0005198) | 260 | -0.23976 | -0.93812 | 0.472441 | 1 |
| OXIDOREDUCTASE_ACTIVITY__ACTING_ON_THE_ALDEHYDE_OR_OXO_GROUP_OF_DONORS(GO:0016903) | 50 | -0.29062 | -0.93333 | 0.605941 | 1 |
| BIOLOGICAL_PROCESS(GO:0008150) | 4493 | -0.17567 | -0.93182 | 0.698819 | 1 |
| INORGANIC_MOLECULAR_ENTITY_TRANSMEMBRANE_TRANSPORTER_ACTIVITY(GO:0015318) | 213 | -0.25344 | -0.92834 | 0.507042 | 1 |
| OXIDOREDUCTASE_ACTIVITY__ACTING_ON_THE_ALDEHYDE_OR_OXO_GROUP_OF_DONORS__NAD_OR_NADP_AS_ACCEPTOR(GO:0016620) | 43 | -0.31304 | -0.92369 | 0.614458 | 1 |
| PROTEOLYSIS(GO:0006508) | 347 | -0.18806 | -0.91754 | 0.612205 | 1 |
| CATION_TRANSMEMBRANE_TRANSPORTER_ACTIVITY(GO:0008324) | 117 | -0.2143 | -0.91701 | 0.724409 | 1 |
| TRANSFERASE_ACTIVITY__TRANSFERRING_ONE_CARBON_GROUPS(GO:0016741) | 55 | -0.20024 | -0.9131 | 0.517103 | 1 |
| ION_GATED_CHANNEL_ACTIVITY(GO:0022839) | 62 | -0.28924 | -0.91302 | 0.596311 | 1 |
| GATED_CHANNEL_ACTIVITY(GO:0022836) | 62 | -0.28924 | -0.91302 | 0.596311 | 1 |
| PEPTIDASE_REGULATOR_ACTIVITY(GO:0061134) | 57 | -0.24812 | -0.91089 | 0.897683 | 1 |
| PEPTIDASE_INHIBITOR_ACTIVITY(GO:0030414) | 57 | -0.24812 | -0.91089 | 0.897683 | 1 |
| TRANSMEMBRANE_TRANSPORT(GO:0055085) | 303 | -0.24696 | -0.90536 | 0.402415 | 1 |
| MAGNESIUM_ION_BINDING(GO:0000287) | 24 | -0.2235 | -0.89951 | 0.772 | 1 |
| COENZYME_BINDING(GO:0050662) | 90 | -0.226 | -0.89509 | 0.710891 | 1 |
| CHROMATIN_BINDING(GO:0003682) | 16 | -0.3109 | -0.89497 | 0.490982 | 1 |
| HEME_BINDING(GO:0020037) | 82 | -0.24176 | -0.89465 | 0.792757 | 1 |
| TRANSPORT(GO:0006810) | 586 | -0.20413 | -0.89373 | 0.6 | 1 |
| ESTABLISHMENT_OF_LOCALIZATION(GO:0051234) | 588 | -0.20377 | -0.89258 | 0.6 | 1 |
| RIBOSOME(GO:0005840) | 229 | -0.24396 | -0.88852 | 0.472441 | 1 |

| NAME | SIZE | ES | NES | NOM p-value | FDR q-value |
|---|---|---|---|---|---|
| ENDONUCLEASE_ACTIVITY(GO:0004519) | 16 | -0.33622 | -0.88692 | 0.596422 | 1 |
| INORGANIC_CATION_TRANSMEMBRANE_TRANSPORTER_ACTIVITY(GO:0022890) | 102 | -0.21962 | -0.87974 | 0.722441 | 1 |
| CARBOHYDRATE_BINDING(GO:0030246) | 30 | -0.33932 | -0.87296 | 0.698473 | 1 |
| HYDROLASE_ACTIVITY__ACTING_ON_GLYCOSYL_BONDS(GO:0016798) | 45 | -0.27521 | -0.86967 | 0.687023 | 1 |
| PHOSPHOPROTEIN_PHOSPHATASE_ACTIVITY(GO:0004721) | 58 | -0.17789 | -0.86782 | 0.898855 | 1 |
| STRUCTURAL_CONSTITUENT_OF_RIBOSOME(GO:0003735) | 228 | -0.24685 | -0.8676 | 0.536913 | 1 |
| LOCALIZATION(GO:0051179) | 602 | -0.19827 | -0.86642 | 0.6 | 1 |
| CALCIUM_ION_BINDING(GO:0005509) | 259 | -0.18728 | -0.86041 | 0.599624 | 1 |
| REGULATION_OF_PHOSPHATE_METABOLIC_PROCESS(GO:0019220) | 18 | -0.27576 | -0.85952 | 0.589494 | 1 |
| REGULATION_OF_PHOSPHORUS_METABOLIC_PROCESS(GO:0051174) | 18 | -0.27576 | -0.85952 | 0.589494 | 1 |
| CARBOHYDRATE_METABOLIC_PROCESS(GO:0005975) | 106 | -0.19588 | -0.85944 | 0.814886 | 1 |
| REGULATION_OF_CELL_CYCLE(GO:0051726) | 20 | -0.22608 | -0.85885 | 0.717391 | 1 |
| TRANSFERASE_ACTIVITY__TRANSFERRING_ACYL_GROUPS_OTHER_THAN_AMINO_ACYL_GROUPS(GO:0016747) | 32 | -0.29132 | -0.85769 | 0.70229 | 1 |
| NAD_BINDING(GO:0051287) | 20 | -0.23552 | -0.85533 | 0.686235 | 1 |
| TRNA_PROCESSING(GO:0008033) | 18 | -0.22828 | -0.85515 | 0.57377 | 1 |
| CELL_ADHESION(GO:0007155) | 116 | -0.26253 | -0.8551 | 0.702703 | 0.998159 |
| BIOLOGICAL_ADHESION(GO:0022610) | 116 | -0.26253 | -0.8551 | 0.702703 | 0.992218 |
| NUCLEASE_ACTIVITY(GO:0004518) | 34 | -0.22723 | -0.85501 | 0.724753 | 0.98666 |
| ANATOMICAL_STRUCTURE_DEVELOPMENT(GO:0048856) | 58 | -0.30323 | -0.85403 | 0.617357 | 0.984242 |
| DEVELOPMENTAL_PROCESS(GO:0032502) | 59 | -0.30112 | -0.85078 | 0.617357 | 0.986906 |
| HYDROLASE_ACTIVITY__ACTING_ON_ESTER_BONDS(GO:0016788) | 180 | -0.17335 | -0.84657 | 0.795802 | 0.991393 |

| NAME | SIZE | ES | NES | NOM p-value | FDR q-value |
|---|---|---|---|---|---|
| MULTICELLULAR_ORGANISMAL_PROCESS(GO:0032501) | 86 | -0.24996 | -0.84583 | 0.897881 | 0.98744 |
| ENZYME_INHIBITOR_ACTIVITY(GO:0004857) | 70 | -0.23995 | -0.84314 | 0.792636 | 0.987175 |
| MACROMOLECULE_METABOLIC_PROCESS(GO:0043170) | 1888 | -0.14126 | -0.83931 | 0.814889 | 0.990211 |
| CELL_CYCLE_PROCESS(GO:0022402) | 29 | -0.18578 | -0.83913 | 0.688109 | 0.985069 |
| ENDOPEPTIDASE_INHIBITOR_ACTIVITY(GO:0004866) | 49 | -0.23204 | -0.83388 | 0.801527 | 0.990085 |
| ENDOPEPTIDASE_REGULATOR_ACTIVITY(GO:0061135) | 49 | -0.23204 | -0.83388 | 0.801527 | 0.984522 |
| RIBONUCLEOPROTEIN_COMPLEX(GO:1990904) | 250 | -0.22745 | -0.83347 | 0.580709 | 0.980262 |
| MONOOXYGENASE_ACTIVITY(GO:0004497) | 16 | -0.36209 | -0.83284 | 0.597586 | 0.976921 |
| CELLULAR_NITROGEN_COMPOUND_CATABOLIC_PROCESS(GO:0044270) | 36 | -0.24926 | -0.82834 | 0.815891 | 0.979191 |
| HETEROCYCLE_CATABOLIC_PROCESS(GO:0046700) | 36 | -0.24926 | -0.82834 | 0.815891 | 0.973811 |
| PEPTIDYL_AMINO_ACID_MODIFICATION(GO:0018193) | 46 | -0.19956 | -0.8265 | 0.598425 | 0.971732 |
| METAL_ION_TRANSPORT(GO:0030001) | 58 | -0.21024 | -0.82464 | 0.679208 | 0.969925 |
| RIBONUCLEOPROTEIN_COMPLEX_BIOGENESIS(GO:0022613) | 30 | -0.19605 | -0.8226 | 0.621094 | 0.968505 |
| PROTEIN_METABOLIC_PROCESS(GO:0019538) | 1132 | -0.15278 | -0.81383 | 0.818898 | 0.98129 |
| PEPTIDYL_PROLYL_CIS_TRANS_ISOMERASE_ACTIVITY(GO:0003755) | 25 | -0.29877 | -0.81279 | 0.785425 | 0.977656 |
| CIS_TRANS_ISOMERASE_ACTIVITY(GO:0016859) | 25 | -0.29877 | -0.81279 | 0.785425 | 0.972456 |
| TETRAPYRROLE_BINDING(GO:0046906) | 86 | -0.21424 | -0.80289 | 0.895669 | 0.98106 |
| INTRACELLULAR_NON_MEMBRANE_BOUNDED_ORGANELLE(GO:0043232) | 424 | -0.18043 | -0.80149 | 0.897287 | 0.978354 |
| NON_MEMBRANE_BOUNDED_ORGANELLE(GO:0043228) | 424 | -0.18043 | -0.80149 | 0.897287 | 0.973232 |
| CARBON_CARBON_LYASE_ACTIVITY(GO:0016830) | 16 | -0.27089 | -0.80006 | 0.688109 | 0.971474 |
| CELLULAR_NITROGEN_COMPOUND_METABOLIC_PROCESS(GO:0034641) | 1142 | -0.12791 | -0.79889 | 0.893788 | 0.969706 |
| CYTOSKELETAL_PART(GO:0044430) | 85 | -0.24033 | -0.79139 | 0.79922 | 0.973991 |

| NAME | SIZE | ES | NES | NOM p-value | FDR q-value |
|---|---|---|---|---|---|
| ORGANONITROGEN_COMPOUND_METABOLIC_PROCESS(GO:1901564) | 1248 | -0.14406 | -0.7903 | 0.895669 | 0.970376 |
| MULTICELLULAR_ORGANISM_DEVELOPMENT(GO:0007275) | 50 | -0.27223 | -0.78439 | 0.704142 | 0.975518 |
| OXIDOREDUCTASE_ACTIVITY__ACTING_ON_PAIRED_DONORS__WITH_INCORPORATION_OR_REDUCTION_OF_MOLECULAR_OXYGEN(GO:0016705) | 81 | -0.24256 | -0.78408 | 0.788538 | 0.970965 |
| PHOSPHOLIPID_METABOLIC_PROCESS(GO:0006644) | 35 | -0.20407 | -0.78355 | 0.698225 | 0.966966 |
| RESPONSE_TO_STRESS(GO:0006950) | 90 | -0.17921 | -0.78306 | 0.898855 | 0.963173 |
| METHYLTRANSFERASE_ACTIVITY(GO:0008168) | 50 | -0.16081 | -0.77976 | 0.89505 | 0.962385 |
| ENZYME_REGULATOR_ACTIVITY(GO:0030234) | 118 | -0.17519 | -0.77664 | 0.900376 | 0.963088 |
| PEPTIDASE_COMPLEX(GO:1905368) | 31 | -0.25103 | -0.77578 | 0.710794 | 0.959429 |
| CELLULAR_MACROMOLECULE_CATABOLIC_PROCESS(GO:0044265) | 49 | -0.19236 | -0.76366 | 0.717054 | 0.968074 |
| PLASMA_MEMBRANE_PROTEIN_COMPLEX(GO:0098797) | 27 | -0.21049 | -0.75742 | 0.801181 | 0.970856 |
| PHOSPHORIC_ESTER_HYDROLASE_ACTIVITY(GO:0042578) | 102 | -0.16234 | -0.75215 | 0.795802 | 0.973293 |
| PROTEIN_DIMERIZATION_ACTIVITY(GO:0046983) | 160 | -0.18784 | -0.75143 | 0.898855 | 0.970047 |
| TRANSFERASE_COMPLEX__TRANSFERRING_PHOSPHORUS_CONTAINING_GROUPS(GO:0061695) | 20 | -0.23884 | -0.74812 | 0.793456 | 0.96964 |
| PEPTIDE_BIOSYNTHETIC_PROCESS(GO:0043043) | 283 | -0.18615 | -0.74428 | 0.897287 | 0.971639 |
| AMIDE_BIOSYNTHETIC_PROCESS(GO:0043604) | 284 | -0.18464 | -0.74055 | 0.897287 | 0.970934 |
| SULFOTRANSFERASE_ACTIVITY(GO:0008146) | 30 | -0.25552 | -0.73575 | 0.894422 | 0.974059 |
| TRANSLATION(GO:0006412) | 281 | -0.18421 | -0.73268 | 0.897287 | 0.972271 |
| CATION_TRANSPORT(GO:0006812) | 110 | -0.16026 | -0.72747 | 0.894632 | 0.976088 |
| RNA_SPLICING(GO:0008380) | 18 | -0.24785 | -0.72607 | 0.897287 | 0.972537 |
| PEPTIDE_METABOLIC_PROCESS(GO:0006518) | 288 | -0.17824 | -0.70897 | 0.897287 | 0.987321 |
| CELLULAR_AMIDE_METABOLIC_PROCESS(GO:0043603) | 290 | -0.17518 | -0.70021 | 0.897287 | 0.988945 |
| TRANSFERASE_ACTIVITY__TRANSFERRING_SULFUR_CONTA | 32 | -0.23658 | -0.69781 | 0.897683 | 0.986383 |

| NAME | SIZE | ES | NES | NOM p-value | FDR q-value |
|---|---|---|---|---|---|
| INING_GROUPS(GO:0016782) | | | | | |
| NUCLEAR_DNA_DIRECTED_RNA_POLYMERASE_COMPLEX(GO:0055029) | 16 | -0.26111 | -0.69767 | 0.793456 | 0.982455 |
| DNA_DIRECTED_RNA_POLYMERASE_COMPLEX(GO:0000428) | 16 | -0.26111 | -0.69767 | 0.793456 | 0.977948 |
| PEPTIDYL_PROLINE_MODIFICATION(GO:0018208) | 22 | -0.27241 | -0.69561 | 0.785425 | 0.976636 |
| PROTEIN_PEPTIDYL_PROLYL_ISOMERIZATION(GO:0000413) | 22 | -0.27241 | -0.69561 | 0.785425 | 0.972197 |
| CYTOSKELETON(GO:0005856) | 99 | -0.20868 | -0.68902 | 0.896686 | 0.970894 |
| ISOMERASE_ACTIVITY(GO:0016853) | 56 | -0.15344 | -0.68739 | 0.892713 | 0.968242 |
| IRON_ION_BINDING(GO:0005506) | 77 | -0.20814 | -0.68585 | 0.897287 | 0.965873 |
| PLASMA_MEMBRANE(GO:0005886) | 40 | -0.19695 | -0.6788 | 0.791423 | 0.969906 |
| CELL_CELL_SIGNALING(GO:0007267) | 24 | -0.28718 | -0.67799 | 0.897087 | 0.967461 |
| MACROMOLECULE_CATABOLIC_PROCESS(GO:0009057) | 53 | -0.17249 | -0.65679 | 0.804971 | 0.977278 |
| GTP_BINDING(GO:0005525) | 177 | -0.14388 | -0.64823 | 0.893788 | 0.981773 |
| PURINE_RIBONUCLEOSIDE_BINDING(GO:0032550) | 177 | -0.14388 | -0.64823 | 0.893788 | 0.977467 |
| PURINE_NUCLEOSIDE_BINDING(GO:0001883) | 177 | -0.14388 | -0.64823 | 0.893788 | 0.973199 |
| RIBONUCLEOSIDE_BINDING(GO:0032549) | 177 | -0.14388 | -0.64823 | 0.893788 | 0.968968 |
| GUANYL_RIBONUCLEOTIDE_BINDING(GO:0032561) | 177 | -0.14388 | -0.64823 | 0.893788 | 0.964773 |
| NUCLEOSIDE_BINDING(GO:0001882) | 177 | -0.14388 | -0.64823 | 0.893788 | 0.960614 |
| PROTEIN_PHOSPHORYLATION(GO:0006468) | 328 | -0.16505 | -0.6357 | 0.789474 | 0.967001 |
| CELL_CELL_SIGNALING_BY_WNT(GO:0198738) | 19 | -0.25941 | -0.62917 | 0.896686 | 0.969146 |
| CELL_SURFACE_RECEPTOR_SIGNALING_PATHWAY_INVOLVED_IN_CELL_CELL_SIGNALING(GO:1905114) | 19 | -0.25941 | -0.62917 | 0.896686 | 0.965022 |
| WNT_SIGNALING_PATHWAY(GO:0016055) | 19 | -0.25941 | -0.62917 | 0.896686 | 0.960933 |
| RNA_POLYMERASE_COMPLEX(GO:0030880) | 18 | -0.2106 | -0.61261 | 0.891616 | 0.967348 |
| MYOSIN_COMPLEX(GO:0016459) | 30 | -0.24161 | -0.61136 | 0.896686 | 0.963507 |

| NAME | SIZE | ES | NES | NOM p-value | FDR q-value |
|---|---|---|---|---|---|
| CELLULAR_DEVELOPMENTAL_PROCESS(GO:0048869) | 15 | -0.23279 | -0.5785 | 0.895669 | 0.977213 |
| G_PROTEIN_COUPLED_RECEPTOR_BINDING(GO:0001664) | 16 | -0.22559 | -0.56875 | 0.897087 | 0.976564 |
| DIVALENT_INORGANIC_CATION_TRANSPORT(GO:0072511) | 15 | -0.22311 | -0.51222 | 0.89505 | 0.989968 |
| DIVALENT_METAL_ION_TRANSPORT(GO:0070838) | 15 | -0.22311 | -0.51222 | 0.89505 | 0.985877 |
