# Supplementary material for: Possible Role of Gut Microbiota in Polypropylene Microplastics-Induced Immunity and Reproductive Dysfunction in Mice
Source: Toxics. 2026 Jul 31;14(8):679. doi: 10.3390/toxics14080679 (PMC13517389; doi:10.3390/toxics14080679)
Supplement: Supplementary file 1 [file toxics-14-00679-s001.zip › Supplementary Information S3.pdf]

Supplementary Information S3. GSEA of enriched GO Terms in the PP group.

NAME	SIZE	ES	NES	NOM p-value	FDR q-value
CELL_REDOX_HOMEOSTASIS(GO:0045454)	30	0.569181	1.692243	0	1
PROTEIN_LOCALIZATION_TO_ORGANELLE(GO:0033365)	21	0.471667	1.557709	0	1
REGULATION_OF_TRANSCRIPTION_BY_RNA_POLYMERASE_II(GO:0006357)	21	0.473981	1.53206	0	1
MITOTIC_CELL_CYCLE(GO:0000278)	15	0.376382	1.492514	0	1
WHOLE_MEMBRANE(GO:0098805)	31	0.420798	1.487404	0	1
TRANSCRIPTION_FACTOR_ACTIVITY__TRANSCRIPTION_FACTOR_BINDING(GO:0000989)	26	0.450783	1.484732	0	1
TRANSCRIPTION_FACTOR_ACTIVITY__PROTEIN_BINDING(GO:0000988)	26	0.450783	1.484732	0	1
TRANSCRIPTION_COFACTOR_ACTIVITY(GO:0003712)	26	0.450783	1.484732	0	1
ORGANIC_ACID_BIOSYNTHETIC_PROCESS(GO:0016053)	20	0.606327	1.475293	0	1
CARBOXYLIC_ACID_BIOSYNTHETIC_PROCESS(GO:0046394)	20	0.606327	1.475293	0	1
THIOL_DEPENDENT_UBIQUITINYL_HYDROLASE_ACTIVITY(GO:0036459)	41	0.42036	1.469917	0	1
UBIQUITINYL_HYDROLASE_ACTIVITY(GO:0101005)	41	0.42036	1.469917	0	1
UBIQUITIN_LIKE_PROTEIN_SPECIFIC_PROTEASE_ACTIVITY(GO:0019783)	41	0.42036	1.469917	0	1
TRNA_AMINOACYLATION_FOR_PROTEIN_TRANSLATION(GO:0006418)	30	0.482843	1.44815	0	1
CELLULAR_LOCALIZATION(GO:0051641)	107	0.38624	1.440167	0	1
AMIDE_TRANSPORT(GO:0042886)	91	0.365547	1.440032	0	1
PROTEIN_LOCALIZATION(GO:0008104)	95	0.366549	1.43745	0.1125	1
CELLULAR_PROTEIN_LOCALIZATION(GO:0034613)	83	0.377197	1.431961	0.1125	1
CELLULAR_MACROMOLECULE_LOCALIZATION(GO:0070727)	83	0.377197	1.431961	0.1125	1
PEPTIDE_TRANSPORT(GO:0015833)	89	0.368661	1.431828	0	1
PROTEIN_TRANSPORT(GO:0015031)	89	0.368661	1.431828	0	1
ESTABLISHMENT_OF_PROTEIN_LOCALIZATION(GO:0045184)	90	0.368266	1.431468	0	0.989683
INTRACELLULAR_TRANSPORT(GO:0046907)	89	0.380491	1.426947	0	0.972281
PURINE_RIBONUCLEOTIDE_BIOSYNTHETIC_PROCESS(GO:0009152)	33	0.386311	1.421426	0.09697	0.969856
RIBOSE_PHOSPHATE_BIOSYNTHETIC_PROCESS(GO:0046390)	33	0.386311	1.421426	0.09697	0.931062
RIBONUCLEOTIDE_BIOSYNTHETIC_PROCESS(GO:0009260)	33	0.386311	1.421426	0.09697	0.895252
CYTOPLASMIC_VESICLE(GO:0031410)	22	0.497238	1.420076	0	0.869542
VESICLE(GO:0031982)	22	0.497238	1.420076	0	0.838487
INTRACELLULAR_VESICLE(GO:0097708)	22	0.497238	1.420076	0	0.809574
INTRACELLULAR_PROTEIN_TRANSPORT(GO:0006886)	76	0.372099	1.416717	0	0.802267
ESTABLISHMENT_OF_LOCALIZATION_IN_CELL(GO:0051649)	90	0.377557	1.413481	0	0.798911
RIBONUCLEOTIDE_METABOLIC_PROCESS(GO:0009259)	43	0.375389	1.409197	0	0.789025
PURINE_RIBONUCLEOTIDE_METABOLIC_PROCESS(GO:0009150)	43	0.375389	1.409197	0	0.765115
RIBOSE_PHOSPHATE_METABOLIC_PROCESS(GO:0019693)	44	0.369753	1.400446	0	0.785177
ENDOMEMBRANE_SYSTEM(GO:0012505)	91	0.374803	1.39928	0	0.780505
MONOCARBOXYLIC_ACID_METABOLIC_PROCESS(GO:0032787)	21	0.528613	1.395392	0.098361	0.767758
PURINE_CONTAINING_COMPOUND_METABOLIC_PROCESS(GO:0072521)	47	0.35555	1.390051	0	0.773812
MONOCARBOXYLIC_ACID_BIOSYNTHETIC_PROCESS(GO:0072330)	15	0.605065	1.385897	0.100604	0.770988
CARBOHYDRATE_DERIVATIVE_METABOLIC_PROCESS(GO:1901135)	110	0.346865	1.381257	0	0.783062
NITROGEN_COMPOUND_TRANSPORT(GO:0071705)	106	0.348901	1.380929	0.1125	0.764811
UBIQUITIN_PROTEIN_TRANSFERASE_ACTIVITY(GO:0004842)	29	0.501098	1.378215	0	0.760335
UBIQUITIN_LIKE_PROTEIN_TRANSFERASE_ACTIVITY(GO:0019787)	29	0.501098	1.378215	0	0.742232

NAME	SIZE	ES	NES	NOM p-value	FDR q-value
PURINE_NUCLEOTIDE_METABOLIC_PROCESS(GO:0006163)	46	0.362054	1.375757	0	0.732076
LIGASE_ACTIVITY(GO:0016874)	61	0.371136	1.358471	0.106719	0.789569
NUCLEOSIDE_MONOPHOSPHATE_BIOSYNTHETIC_PROCESS(GO:0009124)	21	0.527818	1.355276	0.100604	0.799771
PURINE_NUCLEOSIDE_MONOPHOSPHATE_BIOSYNTHETIC_PROCESS(GO:0009127)	21	0.527818	1.355276	0.100604	0.782384
PURINE_RIBONUCLEOSIDE_MONOPHOSPHATE_BIOSYNTHETIC_PROCESS(GO:0009168)	21	0.527818	1.355276	0.100604	0.765738
RIBONUCLEOSIDE_MONOPHOSPHATE_BIOSYNTHETIC_PROCESS(GO:0009156)	21	0.527818	1.355276	0.100604	0.749785
ACTIN_CYTOSKELETON_ORGANIZATION(GO:0030036)	27	0.42977	1.35401	0.28373	0.742114
ACTIN_FILAMENT_BASED_PROCESS(GO:0030029)	27	0.42977	1.35401	0.28373	0.727272
ORGANELLE_ORGANIZATION(GO:0006996)	145	0.27216	1.349197	0.185111	0.727662
NUCLEOBASE_CONTAINING_SMALL_MOLECULE_BIOSYNTHETIC_PROCESS(GO:0034404)	23	0.485625	1.339658	0.100604	0.766703
REGULATION_OF_ANATOMICAL_STRUCTURE_SIZE(GO:0090066)	17	0.573396	1.337422	0.194274	0.760658
MRNA_METABOLIC_PROCESS(GO:0016071)	38	0.2608	1.336633	0	0.747553
PURINE_CONTAINING_COMPOUND_BIOSYNTHETIC_PROCESS(GO:0072522)	37	0.347223	1.335949	0.09697	0.73569
PURINE_NUCLEOTIDE_BIOSYNTHETIC_PROCESS(GO:0006164)	36	0.353909	1.331891	0.19798	0.742738
ESTABLISHMENT_OF_PROTEIN_LOCALIZATION_TO_ORGANELLE(GO:0072594)	17	0.471509	1.325254	0.18591	0.752629
PURINE_NUCLEOSIDE_MONOPHOSPHATE_METABOLIC_PROCESS(GO:0009126)	29	0.491329	1.324261	0.100604	0.745696
PURINE_RIBONUCLEOSIDE_MONOPHOSPHATE_METABOLIC_PROCESS(GO:0009167)	29	0.491329	1.324261	0.100604	0.733057
RIBONUCLEOSIDE_MONOPHOSPHATE_METABOLIC_PROCESS(GO:0009161)	29	0.491329	1.324261	0.100604	0.720839
NUCLEOSIDE_MONOPHOSPHATE_METABOLIC_PROCESS(GO:0009123)	29	0.491329	1.324261	0.100604	0.709022
PROTEIN_MODIFICATION_BY_SMALL_PROTEIN_CONJUGATION_OR_REMOVAL(GO:0070647)	49	0.419729	1.314216	0.199584	0.744106
COFACTOR_METABOLIC_PROCESS(GO:0051186)	48	0.356011	1.309789	0.194059	0.747152
ORGANELLE_FISSION(GO:0048285)	15	0.295522	1.309399	0.097959	0.738811
RNA_POLYMERASE_ACTIVITY(GO:0097747)	16	0.329031	1.308396	0	0.732147
5'_3'_RNA_POLYMERASE_ACTIVITY(GO:0034062)	16	0.329031	1.308396	0	0.721054
DNA_DIRECTED_5'_3'_RNA_POLYMERASE_ACTIVITY(GO:0003899)	16	0.329031	1.308396	0	0.710292
GOLGI_APPARATUS(GO:0005794)	26	0.428325	1.306837	0.098361	0.714252
CHROMOSOME_ORGANIZATION(GO:0051276)	69	0.282467	1.305693	0.187755	0.706639
NUCLEOCYTOPLASMIC_TRANSPORT(GO:0006913)	15	0.471256	1.295489	0.084291	0.747857
NUCLEAR_TRANSPORT(GO:0051169)	15	0.471256	1.295489	0.084291	0.737324
PHOSPHATIDYLINOSITOL_BINDING(GO:0035091)	40	0.372992	1.294916	0.104449	0.729268
PROTEIN_DEUBIQUITINATION(GO:0016579)	36	0.435794	1.294223	0.199584	0.725627
PROTEIN_MODIFICATION_BY_SMALL_PROTEIN_REMOVAL(GO:0070646)	36	0.435794	1.294223	0.199584	0.715821
TUBULIN_BINDING(GO:0015631)	49	0.357493	1.289311	0.197292	0.726303
DRUG_METABOLIC_PROCESS(GO:0017144)	41	0.355676	1.287983	0.202405	0.722002
GOLGI_APPARATUS_PART(GO:0044431)	21	0.399875	1.287332	0.096386	0.713314
RAS_GTPASE_BINDING(GO:0017016)	62	0.431277	1.283813	0.195122	0.723589
SMALL_GTPASE_BINDING(GO:0031267)	62	0.431277	1.283813	0.195122	0.71443
PHOSPHORIC_DIESTER_HYDROLASE_ACTIVITY(GO:0008081)	30	0.344459	1.282867	0.100402	0.708153
NUCLEOSIDE_PHOSPHATE_CATABOLIC_PROCESS(GO:1901292)	22	0.500248	1.282001	0.100604	0.70594
MEDIATOR_COMPLEX(GO:0016592)	20	0.412233	1.2801	0	0.707248
MICROTUBULE_BINDING(GO:0008017)	42	0.389449	1.278478	0.197292	0.704689
ORGANELLE_ENVELOPE(GO:0031967)	50	0.400065	1.278182	0.194274	0.697562
ENVELOPE(GO:0031975)	50	0.400065	1.278182	0.194274	0.689355

NAME	SIZE	ES	NES	NOM p-value	FDR q-value
PHOSPHOLIPID_BINDING(GO:0005543)	51	0.333754	1.274055	0.106719	0.695988
VESICLE_MEDIATED_TRANSPORT(GO:0016192)	78	0.342224	1.271409	0.217842	0.705716
CARBOHYDRATE_CATABOLIC_PROCESS(GO:0016052)	15	0.601453	1.271148	0.197183	0.698862
ATP_METABOLIC_PROCESS(GO:0046034)	28	0.505391	1.269857	0.100604	0.697211
NUCLEOSIDE_PHOSPHATE_METABOLIC_PROCESS(GO:0006753)	77	0.306705	1.26617	0.104294	0.697556
NUCLEOSIDE_TRIPHOSPHATE_METABOLIC_PROCESS(GO:0009141)	32	0.475167	1.262437	0.100604	0.704831
SECRETION_BY_CELL(GO:0032940)	15	0.358812	1.261251	0.078629	0.701898
CARBOHYDRATE_DERIVATIVE_BIOSYNTHETIC_PROCESS(GO:1901137)	81	0.370722	1.260241	0.104294	0.695464
MITOCHONDRIAL_PART(GO:0044429)	49	0.375932	1.257599	0.19	0.694553
NUCLEOBASE_CONTAINING_SMALL_MOLECULE_METABOLIC_PROCESS(GO:0055086)	86	0.281824	1.255687	0.10099	0.695535
MEMBRANE_COAT(GO:0030117)	18	0.560302	1.253156	0.207317	0.696307
COATED_MEMBRANE(GO:0048475)	18	0.560302	1.253156	0.207317	0.689128
ATP_BIOSYNTHETIC_PROCESS(GO:0006754)	20	0.544969	1.251569	0.100604	0.688669
RIBONUCLEOSIDE_TRIPHOSPHATE_BIOSYNTHETIC_PROCESS(GO:0009201)	20	0.544969	1.251569	0.100604	0.681713
PURINE_NUCLEOSIDE_TRIPHOSPHATE_BIOSYNTHETIC_PROCESS(GO:0009145)	20	0.544969	1.251569	0.100604	0.674896
NUCLEOSIDE_TRIPHOSPHATE_BIOSYNTHETIC_PROCESS(GO:0009142)	20	0.544969	1.251569	0.100604	0.668214
PURINE_RIBONUCLEOSIDE_TRIPHOSPHATE_BIOSYNTHETIC_PROCESS(GO:0009206)	20	0.544969	1.251569	0.100604	0.661663
CYTOSKELETAL_PROTEIN_BINDING(GO:0008092)	106	0.296213	1.250917	0.3	0.656291
TRANSLATION_INITIATION_FACTOR_ACTIVITY(GO:0003743)	20	0.51268	1.23959	0.19	0.690306
HELICASE_ACTIVITY(GO:0004386)	23	0.381379	1.239157	0.200787	0.685018
ACTIN_FILAMENT_ORGANIZATION(GO:0007015)	19	0.45813	1.238447	0.273946	0.680734
MEMBRANE_ORGANIZATION(GO:0061024)	19	0.518654	1.238385	0.114519	0.67542
CELLULAR_HOMEOSTASIS(GO:0019725)	39	0.343471	1.236047	0.263581	0.676435
SMALL_MOLECULE_BIOSYNTHETIC_PROCESS(GO:0044283)	41	0.368567	1.231213	0.100402	0.686697
ENDOPLASMIC_RETICULUM(GO:0005783)	39	0.32133	1.229851	0.188912	0.683761
MICROTUBULE_BASED_MOVEMENT(GO:0007018)	52	0.419557	1.229702	0.318087	0.678079
MITOCHONDRION(GO:0005739)	59	0.318201	1.229158	0.188119	0.673607
GENERATION_OF_PRECURSOR_METABOLITES_AND_ENERGY(GO:0006091)	24	0.54	1.226816	0.202405	0.673867
CYSTEINE_TYPE_PEPTIDASE_ACTIVITY(GO:0008234)	75	0.303471	1.222866	0.201581	0.678228
NUCLEOTIDE_METABOLIC_PROCESS(GO:0009117)	74	0.290378	1.214797	0.104294	0.695442
NICOTINAMIDE_NUCLEOTIDE_BIOSYNTHETIC_PROCESS(GO:0019359)	18	0.489279	1.211931	0.197183	0.694242
NUCLEOTIDE_CATABOLIC_PROCESS(GO:0009166)	19	0.488122	1.210216	0.179074	0.691984
DNA_REPLICATION(GO:0006260)	31	0.290487	1.201986	0.293886	0.706566
PROTEIN_POLYMERIZATION(GO:0051258)	17	0.448762	1.197735	0.191919	0.706969
ORGANOPHOSPHATE_CATABOLIC_PROCESS(GO:0046434)	27	0.404253	1.19653	0.180894	0.704563
ANTIOXIDANT_ACTIVITY(GO:0016209)	18	0.482569	1.192408	0.093567	0.712518
MACROMOLECULE_LOCALIZATION(GO:0033036)	125	0.280377	1.191533	0.196721	0.710306
SUPRAMOLECULAR_FIBER_ORGANIZATION(GO:0097435)	20	0.435399	1.185464	0.273946	0.720047
ORGANIC_SUBSTANCE_TRANSPORT(GO:0071702)	134	0.270721	1.185253	0.19917	0.715277
MRNA_PROCESSING(GO:0006397)	32	0.266823	1.183863	0.282609	0.714944
MICROTUBULE_MOTOR_ACTIVITY(GO:0003777)	49	0.423267	1.181908	0.405405	0.716447
PURINE_RIBONUCLEOSIDE_TRIPHOSPHATE_METABOLIC_PROCESS(GO:0009205)	29	0.452254	1.174223	0.100604	0.738431
PURINE_NUCLEOSIDE_TRIPHOSPHATE_METABOLIC_PROCESS(GO:0009144)	29	0.452254	1.174223	0.100604	0.732662

NAME	SIZE	ES	NES	NOM p-value	FDR q-value
RIBONUCLEOSIDE_TRIPHOSPHATE_METABOLIC_PROCESS(GO:0009199)	29	0.452254	1.174223	0.100604	0.726982
NUCLEAR_PART(GO:0044428)	91	0.216248	1.163049	0.287726	0.765725
NCRNA_METABOLIC_PROCESS(GO:0034660)	64	0.223334	1.155254	0.298137	0.784823
MOVEMENT_OF_CELL_OR_SUBCELLULAR_COMPONENT(GO:0006928)	58	0.405098	1.15479	0.398773	0.780899
ORGANELLE_MEMBRANE(GO:0031090)	72	0.294255	1.15273	0.299795	0.784238
MICROTUBULE_BASED_PROCESS(GO:0007017)	72	0.307024	1.150926	0.27853	0.785952
MEMBRANE_BOUNDED_ORGANELLE(GO:0043227)	511	0.211676	1.148577	0.191667	0.791869
LIGASE_ACTIVITY__FORMING_CARBON_OXYGEN_BONDS(GO:0016875)	33	0.328887	1.146369	0.294479	0.796596
AMINO_ACID_ACTIVATION(GO:0043038)	33	0.328887	1.146369	0.294479	0.790782
TRNA_AMINOACYLATION(GO:0043039)	33	0.328887	1.146369	0.294479	0.785051
AMINOACYL_TRNA_LIGASE_ACTIVITY(GO:0004812)	33	0.328887	1.146369	0.294479	0.779404
PROTEIN_CONTAINING_COMPLEX_DISASSEMBLY(GO:0032984)	16	0.401175	1.144898	0.195072	0.779703
CELLULAR_PROTEIN_COMPLEX_DISASSEMBLY(GO:0043624)	16	0.401175	1.144898	0.195072	0.774173
TRANSLATIONAL_INITIATION(GO:0006413)	17	0.401285	1.134635	0.289738	0.804422
NUCLEOBASE_CONTAINING_COMPOUND_CATABOLIC_PROCESS(GO:0034655)	30	0.392332	1.132	0.279592	0.804511
CARBOXYLIC_ACID_METABOLIC_PROCESS(GO:0019752)	82	0.277597	1.130885	0.201581	0.802736
ORGANIC_ACID_METABOLIC_PROCESS(GO:0006082)	82	0.277597	1.130885	0.201581	0.7972
OXOACID_METABOLIC_PROCESS(GO:0043436)	82	0.277597	1.130885	0.201581	0.79174
INTRACELLULAR_MEMBRANE_BOUNDED_ORGANELLE(GO:0043231)	494	0.205429	1.130153	0.191667	0.788768
CELLULAR_COMPONENT_ORGANIZATION(GO:0016043)	235	0.21288	1.128584	0.081761	0.789977
TRANSFERASE_ACTIVITY__TRANSFERRING_PENTOSYL_GROUPS(GO:0016763)	21	0.383709	1.128272	0.379921	0.788001
ORGANELLE_LUMEN(GO:0043233)	78	0.21411	1.127988	0.287726	0.784762
INTRACELLULAR_ORGANELLE_LUMEN(GO:0070013)	78	0.21411	1.127988	0.287726	0.779564
MEMBRANE_ENCLOSED_LUMEN(GO:0031974)	78	0.21411	1.127988	0.287726	0.774436
CHROMATIN_ORGANIZATION(GO:0006325)	42	0.307931	1.125511	0.292276	0.781342
TRANSLATION_FACTOR_ACTIVITY__RNA_BINDING(GO:0008135)	29	0.364184	1.123182	0.194274	0.786123
CYTOSKELETON_ORGANIZATION(GO:0007010)	52	0.253483	1.119964	0.400786	0.788446
NUCLEOTIDE_BIOSYNTHETIC_PROCESS(GO:0009165)	58	0.265048	1.118936	0.202454	0.785239
NUCLEOSIDE_PHOSPHATE_BIOSYNTHETIC_PROCESS(GO:1901293)	58	0.265048	1.118936	0.202454	0.780238
NUCLEOTIDYLTRANSFERASE_ACTIVITY(GO:0016779)	45	0.307118	1.112521	0.184426	0.802071
SMALL_MOLECULE_METABOLIC_PROCESS(GO:0044281)	180	0.221955	1.111784	0.200787	0.802518
REGULATION_OF_BIOLOGICAL_QUALITY(GO:0065008)	75	0.294543	1.10959	0.269618	0.803633
RHO_GTPASE_BINDING(GO:0017048)	53	0.374211	1.10922	0.195122	0.799357
CELLULAR_COMPONENT_ORGANIZATION_OR_BIOGENESIS(GO:0071840)	259	0.208048	1.10695	0.174004	0.805138
BOUNDING_MEMBRANE_OF_ORGANELLE(GO:0098588)	45	0.247355	1.1033	0.202381	0.820937
NEGATIVE_REGULATION_OF_BIOLOGICAL_PROCESS(GO:0048519)	54	0.238085	1.101936	0.1875	0.82204
CATALYTIC_ACTIVITY__ACTING_ON_A_TRNA(GO:0140101)	44	0.285886	1.10126	0.403326	0.819212
MITOCHONDRIAL_MEMBRANE(GO:0031966)	39	0.356025	1.099983	0.289109	0.818871
ENZYME_BINDING(GO:0019899)	103	0.300093	1.096967	0.199584	0.822607
ORGANELLE_PART(GO:0044422)	362	0.211978	1.088969	0.297917	0.852311
LIPID_BIOSYNTHETIC_PROCESS(GO:0008610)	28	0.328541	1.088544	0.338174	0.848278
ORGANELLE_INNER_MEMBRANE(GO:0019866)	25	0.44463	1.087582	0.298569	0.847306
MITOCHONDRIAL_INNER_MEMBRANE(GO:0005743)	25	0.44463	1.087582	0.298569	0.842351
PROTEIN_FOLDING(GO:0006457)	29	0.381457	1.086783	0.494888	0.839936

NAME	SIZE	ES	NES	NOM p-value	FDR q-value
ACTIN_BINDING(GO:0003779)	48	0.275437	1.086268	0.396728	0.837381
NEGATIVE_REGULATION_OF_NITROGEN_COMPOUND_METABOLIC_PROCESS(GO:0051172)	15	0.321364	1.082832	0.280488	0.842762
ELECTRON_TRANSFER_ACTIVITY(GO:0009055)	31	0.315923	1.081782	0.26378	0.842097
INTRACELLULAR_ORGANELLE_PART(GO:0044446)	359	0.210038	1.081437	0.293033	0.83787
OXIDOREDUCTASE_ACTIVITY__ACTING_ON_THE_CH_CH_GROUP_OF_DONORS(GO:0016627)	20	0.404736	1.078724	0.293878	0.844872
ORGANOPHOSPHATE_METABOLIC_PROCESS(GO:0019637)	118	0.209376	1.074232	0.275154	0.858876
NEGATIVE_REGULATION_OF_METABOLIC_PROCESS(GO:0009892)	22	0.307899	1.072682	0.199584	0.858118
NEGATIVE_REGULATION_OF_CELLULAR_METABOLIC_PROCESS(GO:0031324)	16	0.299621	1.071832	0.280488	0.857936
RHO_PROTEIN_SIGNAL_TRANSDUCTION(GO:0007266)	45	0.383541	1.067256	0.298781	0.871131
REGULATION_OF_RHO_PROTEIN_SIGNAL_TRANSDUCTION(GO:0035023)	45	0.383541	1.067256	0.298781	0.866344
RAS_GUANYL_NUCLEOTIDE_EXCHANGE_FACTOR_ACTIVITY(GO:0005088)	45	0.383541	1.067256	0.298781	0.86161
RHO_GUANYL_NUCLEOTIDE_EXCHANGE_FACTOR_ACTIVITY(GO:0005089)	45	0.383541	1.067256	0.298781	0.856928
OXIDOREDUCTASE_ACTIVITY__ACTING_ON_PEROXIDE_AS_ACCEPTOR(GO:0016684)	16	0.436517	1.063185	0.403509	0.861948
MITOCHONDRIAL_ENVELOPE(GO:0005740)	42	0.338706	1.062683	0.398773	0.859569
COENZYME_METABOLIC_PROCESS(GO:0006732)	34	0.315595	1.056571	0.293886	0.875088
ATPASE_ACTIVITY__COUPLED(GO:0042623)	38	0.339327	1.055705	0.199584	0.876303
NEGATIVE_REGULATION_OF_CELLULAR_PROCESS(GO:0048523)	47	0.220781	1.049773	0.27686	0.883822
NUCLEUS(GO:0005634)	355	0.18026	1.048087	0.401222	0.883787
TRNA_METABOLIC_PROCESS(GO:0006399)	51	0.223232	1.042312	0.496994	0.891687
PURINE_NUCLEOTIDE_BINDING(GO:0017076)	797	0.237221	1.041804	0.405405	0.888774
ORGANOPHOSPHATE_BIOSYNTHETIC_PROCESS(GO:0090407)	77	0.219457	1.041675	0.203285	0.88531
LIPID_BINDING(GO:0008289)	85	0.275455	1.040821	0.292683	0.88455
COENZYME_BIOSYNTHETIC_PROCESS(GO:0009108)	31	0.333175	1.040806	0.293886	0.880286
PURINE_RIBONUCLEOTIDE_BINDING(GO:0032555)	786	0.23617	1.039897	0.405405	0.879733
RAS_PROTEIN_SIGNAL_TRANSDUCTION(GO:0007265)	53	0.370236	1.038277	0.384146	0.882452
REGULATION_OF_RAS_PROTEIN_SIGNAL_TRANSDUCTION(GO:0046578)	53	0.370236	1.038277	0.384146	0.877995
PURINE_RIBONUCLEOSIDE_TRIPHOSPHATE_BINDING(GO:0035639)	785	0.235208	1.038053	0.405405	0.875495
GLYCEROPHOSPHOLIPID_METABOLIC_PROCESS(GO:0006650)	19	0.314935	1.037784	0.379592	0.872025
GLYCEROLIPID_METABOLIC_PROCESS(GO:0046486)	19	0.314935	1.037784	0.379592	0.867687
PROTEIN_SERINE_THREONINE_KINASE_ACTIVITY(GO:0004674)	47	0.372308	1.037399	0.404908	0.866575
COFACTOR_BIOSYNTHETIC_PROCESS(GO:0051188)	37	0.290632	1.034852	0.293886	0.869746
FLAVIN_ADENINE_DINUCLEOTIDE_BINDING(GO:0050660)	30	0.29048	1.03304	0.493878	0.870996
ZINC_ION_BINDING(GO:0008270)	246	0.24667	1.032882	0.398773	0.86735
TRANSCRIPTION_BY_RNA_POLYMERASE_II(GO:0006366)	41	0.220947	1.032358	0.387879	0.864594
CARBOHYDRATE_DERIVATIVE_BINDING(GO:0097367)	806	0.237005	1.031192	0.405405	0.865345
MEMBRANE_PROTEIN_COMPLEX(GO:0098796)	95	0.226586	1.027821	0.308468	0.872092
CELLULAR_LIPID_METABOLIC_PROCESS(GO:0044255)	48	0.234465	1.023941	0.386694	0.879337
RIBONUCLEOTIDE_BINDING(GO:0032553)	790	0.232928	1.023615	0.405405	0.87646
NUCLEOTIDE_BINDING(GO:0000166)	885	0.230655	1.022424	0.405405	0.875853
NUCLEOSIDE_PHOSPHATE_BINDING(GO:1901265)	885	0.230655	1.022424	0.405405	0.871721
PROTEIN_TYROSINE_SERINE_THREONINE_PHOSPHATASE_ACTIVITY(GO:0008138)	27	0.243532	1.020662	0.272727	0.871355
NEGATIVE_REGULATION_OF_MACROMOLECULE_METABOLIC_PROCESS(GO:0010605)	21	0.309906	1.01873	0.31185	0.872902
REGULATION_OF_SMALL_GTPASE_MEDIATED_SIGNAL_TRANSDUCTION(GO:0051056)	59	0.366718	1.017283	0.384146	0.872705

NAME	SIZE	ES	NES	NOM p-value	FDR q-value
LYASE_ACTIVITY(GO:0016829)	39	0.245771	1.012821	0.402724	0.882784
GTPASE_BINDING(GO:0051020)	87	0.292277	1.01067	0.384146	0.884331
ANION_BINDING(GO:0043168)	920	0.227463	1.009205	0.405405	0.884282
HYDROLASE_ACTIVITY__ACTING_ON_ACID_ANHYDRIDES__IN_PHOSPHORUS_CONTAINING_ANHYDRIDES(GO:0016818)	306	0.232908	1.009135	0.396341	0.880485
PROTON_TRANSMEMBRANE_TRANSPORT(GO:1902600)	23	0.291067	1.005813	0.196787	0.888173
ADENYL_NUCLEOTIDE_BINDING(GO:0030554)	610	0.264783	1.004374	0.484663	0.889801
REPRODUCTIVE_PROCESS(GO:0022414)	25	0.296973	1.003017	0.507071	0.888154
REPRODUCTION(GO:0000003)	25	0.296973	1.003017	0.507071	0.884171
ADENYL_RIBONUCLEOTIDE_BINDING(GO:0032559)	609	0.264797	1.002458	0.484663	0.881858
DNA_PACKAGING_COMPLEX(GO:0044815)	47	0.303561	1.002171	0.404082	0.87916
HYDROLASE_ACTIVITY__ACTING_ON_ACID_ANHYDRIDES(GO:0016817)	311	0.232733	1.001572	0.396341	0.878102
HOMEOSTATIC_PROCESS(GO:0042592)	44	0.283042	1.001256	0.488934	0.875334
UNFOLDED_PROTEIN_BINDING(GO:0051082)	24	0.373968	1.000052	0.606925	0.872475
ATP_BINDING(GO:0005524)	608	0.263472	0.999621	0.484663	0.869095
PYROPHOSPHATASE_ACTIVITY(GO:0016462)	304	0.230023	0.999106	0.396341	0.866309
SMALL_MOLECULE_BINDING(GO:0036094)	926	0.226011	0.998146	0.405405	0.865351
PYRIDINE_CONTAINING_COMPOUND_BIOSYNTHETIC_PROCESS(GO:0072525)	19	0.385495	0.99795	0.299799	0.862585
PYRIDINE_NUCLEOTIDE_BIOSYNTHETIC_PROCESS(GO:0019363)	19	0.385495	0.99795	0.299799	0.858882
OXIDOREDUCTION_COENZYME_METABOLIC_PROCESS(GO:0006733)	21	0.370277	0.997176	0.299799	0.856796
CELL_SURFACE_RECEPTOR_SIGNALING_PATHWAY(GO:0007166)	55	0.305215	0.996019	0.457831	0.858716
NUCLEOSOME(GO:0000786)	46	0.30387	0.994793	0.404082	0.859713
PROTEIN_DNA_COMPLEX(GO:0032993)	46	0.30387	0.994793	0.404082	0.856086
GLYCOPROTEIN_METABOLIC_PROCESS(GO:0009100)	35	0.354905	0.994392	0.513292	0.854158
GLYCOPROTEIN_BIOSYNTHETIC_PROCESS(GO:0009101)	35	0.354905	0.994392	0.513292	0.850584
PROTEIN_GLYCOSYLATION(GO:0006486)	35	0.354905	0.994392	0.513292	0.84704
GLYCOSYLATION(GO:0070085)	35	0.354905	0.994392	0.513292	0.843525
MACROMOLECULE_GLYCOSYLATION(GO:0043413)	35	0.354905	0.994392	0.513292	0.84004
PROTEIN_HETERODIMERIZATION_ACTIVITY(GO:0046982)	60	0.26932	0.992768	0.404427	0.841412
INTRACELLULAR_SIGNAL_TRANSDUCTION(GO:0035556)	174	0.267803	0.99273	0.481707	0.838366
OXIDOREDUCTASE_ACTIVITY__ACTING_ON_CH_OH_GROUP_OF_DONORS(GO:0016614)	34	0.313197	0.991657	0.42126	0.837631
RNA_PROCESSING(GO:0006396)	92	0.167605	0.990679	0.277132	0.83607
PYRIDINE_CONTAINING_COMPOUND_METABOLIC_PROCESS(GO:0072524)	20	0.374675	0.98697	0.299799	0.84504
PYRIDINE_NUCLEOTIDE_METABOLIC_PROCESS(GO:0019362)	20	0.374675	0.98697	0.299799	0.841633
NICOTINAMIDE_NUCLEOTIDE_METABOLIC_PROCESS(GO:0046496)	20	0.374675	0.98697	0.299799	0.838253
NUCLEOSIDE_TRIPHOSPHATASE_ACTIVITY(GO:0017111)	299	0.226416	0.981096	0.481707	0.851593
MICROTUBULE_CYTOSKELETON(GO:0015630)	33	0.351111	0.974671	0.509203	0.863659
CATALYTIC_ACTIVITY__ACTING_ON_RNA(GO:0140098)	78	0.189012	0.972489	0.496994	0.86428
CATALYTIC_COMPLEX(GO:1902494)	98	0.201474	0.9719	0.497917	0.861452
CELL_CYCLE(GO:0007049)	43	0.158916	0.967257	0.496829	0.867
PROTEIN_DNA_COMPLEX_ASSEMBLY(GO:0065004)	28	0.296842	0.967125	0.587755	0.864773
DRUG_BINDING(GO:0008144)	637	0.253084	0.966807	0.60499	0.861602
PROTEIN_DNA_COMPLEX_SUBUNIT_ORGANIZATION(GO:0071824)	29	0.283396	0.966551	0.510246	0.859242
ATPASE_ACTIVITY(GO:0016887)	69	0.262479	0.962656	0.505198	0.866937

NAME	SIZE	ES	NES	NOM p-value	FDR q-value
CELLULAR_AMINO_ACID_METABOLIC_PROCESS(GO:0006520)	54	0.213545	0.959313	0.492986	0.871281
MODIFICATION_DEPENDENT_MACROMOLECULE_CATABOLIC_PROCESS(GO:0043632)	27	0.304534	0.957105	0.507128	0.874702
MODIFICATION_DEPENDENT_PROTEIN_CATABOLIC_PROCESS(GO:0019941)	27	0.304534	0.957105	0.507128	0.871351
UBIQUITIN_DEPENDENT_PROTEIN_CATABOLIC_PROCESS(GO:0006511)	27	0.304534	0.957105	0.507128	0.868025
NUCLEOPLASM_PART(GO:0044451)	51	0.179709	0.951276	0.57115	0.884473
NUCLEOPLASM(GO:0005654)	51	0.179709	0.951276	0.57115	0.881123
OXIDOREDUCTASE_ACTIVITY__ACTING_ON_THE_CH_OH_GROUP_OF_DONORS__NAD_OR_NADP_AS_ACCEPTOR(GO:0016616)	33	0.299527	0.949422	0.42126	0.881359
PHOSPHATIDYLINOSITOL_METABOLIC_PROCESS(GO:0046488)	18	0.28026	0.948232	0.504082	0.88019
DYNEIN_COMPLEX(GO:0030286)	19	0.500232	0.947318	0.478528	0.879661
ION_BINDING(GO:0043167)	1597	0.208642	0.9473	0.517672	0.876576
REGULATION_OF_CELLULAR_COMPONENT_BIOGENESIS(GO:0044087)	15	0.377264	0.945519	0.486538	0.877285
LIPID_METABOLIC_PROCESS(GO:0006629)	103	0.22613	0.945288	0.474012	0.875534
PROTON_TRANSPORTING_TWO_SECTOR_ATPASE_COMPLEX(GO:0016469)	15	0.34993	0.941556	0.281071	0.882337
NUCLEAR_LUMEN(GO:0031981)	70	0.176394	0.937897	0.480885	0.885709
DNA_CONFORMATION_CHANGE(GO:0071103)	29	0.276917	0.936686	0.686071	0.885996
NUCLEOBASE_CONTAINING_COMPOUND_BIOSYNTHETIC_PROCESS(GO:0034654)	610	0.174208	0.933479	0.708087	0.890844
NUCLEIC_ACID_BINDING(GO:0003676)	1196	0.148056	0.932726	0.520325	0.890775
HETEROCYCLE_BIOSYNTHETIC_PROCESS(GO:0018130)	621	0.175452	0.932669	0.708087	0.888139
RIBOSOME_BIOGENESIS(GO:0042254)	25	0.229652	0.932186	0.416499	0.885916
PROTON_TRANSMEMBRANE_TRANSPORTER_ACTIVITY(GO:0015078)	37	0.269618	0.930977	0.386831	0.885998
REGULATION_OF_INTRACELLULAR_SIGNAL_TRANSDUCTION(GO:1902531)	65	0.338266	0.929702	0.384146	0.885508
ORGANIC_CYCLIC_COMPOUND_BINDING(GO:0097159)	2097	0.16817	0.928974	0.591463	0.88502
HETEROCYCLIC_COMPOUND_BINDING(GO:1901363)	2097	0.16817	0.928974	0.591463	0.881871
CELLULAR_PROTEIN_CONTAINING_COMPLEX_ASSEMBLY(GO:0034622)	69	0.252216	0.928145	0.806122	0.88176
AROMATIC_COMPOUND_BIOSYNTHETIC_PROCESS(GO:0019438)	618	0.17432	0.927197	0.708087	0.883654
CELLULAR_METABOLIC_PROCESS(GO:0044237)	1832	0.14432	0.918308	0.708163	0.903393
ORGANIC_CYCLIC_COMPOUND_BIOSYNTHETIC_PROCESS(GO:1901362)	627	0.1737	0.918153	0.708087	0.900409
BINDING(GO:0005488)	4569	0.181407	0.914779	0.60499	0.903469
RNA_METABOLIC_PROCESS(GO:0016070)	686	0.162437	0.913149	0.694391	0.903437
INTRACELLULAR_PART(GO:0044424)	1028	0.159711	0.912114	0.682828	0.904214
NUCLEOBASE_CONTAINING_COMPOUND_METABOLIC_PROCESS(GO:0006139)	867	0.148131	0.911212	0.793037	0.904883
GTPASE_ACTIVITY(GO:0003924)	136	0.179689	0.910504	0.604888	0.903613
HETEROCYCLE_METABOLIC_PROCESS(GO:0046483)	885	0.148075	0.908336	0.694391	0.904599
DNA_PACKAGING(GO:0006323)	25	0.29385	0.907526	0.598753	0.902327
CATALYTIC_ACTIVITY(GO:0003824)	2346	0.182626	0.90601	0.591463	0.905382
TRANSCRIPTION_REGULATOR_ACTIVITY(GO:0140110)	218	0.25297	0.901088	0.493902	0.91271
MITOCHONDRIAL_PROTEIN_COMPLEX(GO:0098798)	27	0.33415	0.898615	0.408998	0.914335
INNER_MITOCHONDRIAL_MEMBRANE_PROTEIN_COMPLEX(GO:0098800)	18	0.386314	0.895048	0.695297	0.923563
CHROMATIN_ASSEMBLY(GO:0031497)	23	0.299082	0.894087	0.68476	0.92428
NUCLEOSOME_ASSEMBLY(GO:0006334)	23	0.299082	0.894087	0.68476	0.921179
TRANSFERASE_ACTIVITY__TRANSFERRING_GLYCOSYL_GROUPS(GO:0016757)	114	0.238131	0.89362	0.678937	0.918881
HYDROLASE_ACTIVITY(GO:0016787)	996	0.174414	0.891943	0.697342	0.918317
NUCLEIC_ACID_TEMPLATED_TRANSCRIPTION(GO:0097659)	543	0.169165	0.889804	0.694391	0.920222

NAME	SIZE	ES	NES	NOM p-value	FDR q-value
RNA_BIOSYNTHETIC_PROCESS(GO:0032774)	543	0.169165	0.889804	0.694391	0.917175
TRANSCRIPTION__DNA_TEMPLATED(GO:0006351)	543	0.169165	0.889804	0.694391	0.914148
CHROMOSOME(GO:0005694)	89	0.204248	0.887395	0.60521	0.915074
ORGANIC_CYCLIC_COMPOUND_METABOLIC_PROCESS(GO:1901360)	896	0.145286	0.886668	0.793037	0.91392
CELLULAR_AROMATIC_COMPOUND_METABOLIC_PROCESS(GO:0006725)	886	0.144218	0.886446	0.793834	0.912354
ION_TRANSMEMBRANE_TRANSPORT(GO:0034220)	39	0.256337	0.879354	0.786139	0.928904
REGULATION_OF_GENE_EXPRESSION(GO:0010468)	497	0.174613	0.872341	0.598425	0.942377
NUCLEIC_ACID_METABOLIC_PROCESS(GO:0090304)	778	0.143387	0.872122	0.897486	0.939768
RNA_BINDING(GO:0003723)	175	0.176874	0.87015	0.693069	0.93974
REGULATION_OF_METABOLIC_PROCESS(GO:0019222)	524	0.169529	0.869686	0.706693	0.937225
CHROMOSOMAL_PART(GO:0044427)	79	0.216838	0.869371	0.683367	0.934554
TRANSITION_METAL_ION_BINDING(GO:0046914)	349	0.212116	0.869066	0.697342	0.931737
PROTEIN_CONTAINING_COMPLEX_ASSEMBLY(GO:0065003)	110	0.194755	0.869058	0.674897	0.928938
DNA_BINDING_TRANSCRIPTION_FACTOR_ACTIVITY(GO:0003700)	186	0.254158	0.868143	0.591463	0.927824
NUCLEOSOME_ORGANIZATION(GO:0034728)	24	0.283628	0.867037	0.68476	0.926223
REGULATION_OF_RNA_BIOSYNTHETIC_PROCESS(GO:2001141)	485	0.173675	0.86647	0.706693	0.926256
REGULATION_OF_NUCLEIC_ACID_TEMPLATED_TRANSCRIPTION(GO:1903506)	485	0.173675	0.86647	0.706693	0.923343
REGULATION_OF_RNA_METABOLIC_PROCESS(GO:0051252)	485	0.173675	0.86647	0.706693	0.920449
REGULATION_OF_TRANSCRIPTION__DNA_TEMPLATED(GO:0006355)	485	0.173675	0.86647	0.706693	0.917572
REGULATION_OF_NUCLEOBASE_CONTAINING_COMPOUND_METABOLIC_PROCESS(GO:0019219)	491	0.171229	0.865956	0.706693	0.915104
CELLULAR_COMPONENT_ASSEMBLY(GO:0022607)	128	0.180941	0.863108	0.687631	0.91787
INTRACELLULAR(GO:0005622)	1235	0.149307	0.862691	0.674847	0.915587
TRANSFERASE_ACTIVITY__TRANSFERRING_ACYL_GROUPS(GO:0016746)	56	0.260549	0.8621	0.704	0.913621
PROTEIN_BINDING(GO:0005515)	2083	0.186111	0.862096	0.576238	0.910974
REGULATION_OF_CELLULAR_METABOLIC_PROCESS(GO:0031323)	517	0.168201	0.859986	0.706693	0.912199
METHYLATION(GO:0032259)	16	0.240224	0.858772	0.514228	0.913698
MOTOR_ACTIVITY(GO:0003774)	79	0.300317	0.858351	0.595092	0.911508
REGULATION_OF_CELLULAR_BIOSYNTHETIC_PROCESS(GO:0031326)	495	0.170606	0.857081	0.706693	0.913317
REGULATION_OF_BIOSYNTHETIC_PROCESS(GO:0009889)	495	0.170606	0.857081	0.706693	0.910549
REGULATION_OF_MACROMOLECULE_BIOSYNTHETIC_PROCESS(GO:0010556)	495	0.170606	0.857081	0.706693	0.907798
REGULATION_OF_CELLULAR_MACROMOLECULE_BIOSYNTHETIC_PROCESS(GO:2000112)	495	0.170606	0.857081	0.706693	0.905064
CHROMATIN(GO:0000785)	54	0.259094	0.856847	0.802041	0.902506
CHROMATIN_ASSEMBLY_OR_DISASSEMBLY(GO:0006333)	25	0.274544	0.854711	0.686071	0.904229
LIGASE_ACTIVITY__FORMING_CARBON_NITROGEN_BONDS(GO:0016879)	17	0.314813	0.854292	0.614173	0.902231
PROTEIN_CONTAINING_COMPLEX_SUBUNIT_ORGANIZATION(GO:0043933)	116	0.184465	0.853547	0.687631	0.900458
CELL(GO:0005623)	1280	0.145783	0.853219	0.893574	0.899087
CELL_PART(GO:0044464)	1280	0.145783	0.853219	0.893574	0.896427
ORGANIC_SUBSTANCE_BIOSYNTHETIC_PROCESS(GO:1901576)	1016	0.146471	0.852978	0.789883	0.89394
REGULATION_OF_MACROMOLECULE_METABOLIC_PROCESS(GO:0060255)	516	0.165954	0.851199	0.78937	0.895547
REGULATION_OF_RESPONSE_TO_STIMULUS(GO:0048583)	98	0.242601	0.85009	0.456	0.894969
METABOLIC_PROCESS(GO:0008152)	2563	0.144639	0.850088	0.891616	0.892506
CELLULAR_COMPONENT_BIOGENESIS(GO:0044085)	152	0.174756	0.850057	0.687631	0.890059
NEGATIVE_REGULATION_OF_GENE_EXPRESSION(GO:0010629)	17	0.305243	0.846752	0.593047	0.896041

NAME	SIZE	ES	NES	NOM p-value	FDR q-value
CELLULAR_BIOSYNTHETIC_PROCESS(GO:0044249)	999	0.144106	0.844735	0.896887	0.898026
REGULATION_OF_NITROGEN_COMPOUND_METABOLIC_PROCESS(GO:0051171)	509	0.164566	0.841959	0.78937	0.900865
REGULATION_OF_PRIMARY_METABOLIC_PROCESS(GO:0080090)	509	0.164566	0.841959	0.78937	0.898269
PRIMARY_METABOLIC_PROCESS(GO:0044238)	2157	0.140649	0.841585	0.811861	0.896924
ORGANIC_SUBSTANCE_METABOLIC_PROCESS(GO:0071704)	2206	0.139937	0.840964	0.811861	0.895667
TRANSFERASE_ACTIVITY(GO:0016740)	749	0.197273	0.840542	0.704	0.893801
OXIDATION_REDUCTION_PROCESS(GO:0055114)	333	0.185613	0.834082	0.892276	0.901497
NITROGEN_COMPOUND_METABOLIC_PROCESS(GO:0006807)	2011	0.137107	0.831593	0.89505	0.903079
BIOSYNTHETIC_PROCESS(GO:0009058)	1038	0.139949	0.828332	0.896887	0.906003
CELLULAR_MACROMOLECULE_METABOLIC_PROCESS(GO:0044260)	1494	0.138677	0.825468	0.894	0.90742
METAL_ION_BINDING(GO:0046872)	709	0.184782	0.818426	0.777096	0.918849
CATION_BINDING(GO:0043169)	711	0.183993	0.817913	0.777096	0.917023
ORGANIC_SUBSTANCE_CATABOLIC_PROCESS(GO:1901575)	102	0.199727	0.817223	0.589537	0.915361
ATPASE_ACTIVITY__COUPLED_TO_TRANSMEMBRANE_MOVEMENT_OF_SUBSTANCES(GO:0042626)	27	0.295157	0.81663	0.681725	0.91466
PRIMARY_ACTIVE_TRANSMEMBRANE_TRANSPORTER_ACTIVITY(GO:0015399)	27	0.295157	0.81663	0.681725	0.912113
P_P_BOND_HYDROLYSIS_DRIVEN_TRANSMEMBRANE_TRANSPORTER_ACTIVITY(GO:0015405)	27	0.295157	0.81663	0.681725	0.909579
ATPASE_ACTIVITY__COUPLED_TO_MOVEMENT_OF_SUBSTANCES(GO:0043492)	27	0.295157	0.81663	0.681725	0.907059
INTRACELLULAR_ORGANELLE(GO:0043229)	884	0.148954	0.815337	0.892929	0.906542
ORGANELLE(GO:0043226)	888	0.148083	0.812906	0.892929	0.906793
MICROTUBULE_ASSOCIATED_COMPLEX(GO:0005875)	23	0.377691	0.811299	0.891616	0.906896
SMALL_GTPASE_MEDIATED_SIGNAL_TRANSDUCTION(GO:0007264)	78	0.239847	0.807726	0.582	0.910156
ENZYME_ACTIVATOR_ACTIVITY(GO:0008047)	31	0.300151	0.803441	0.781186	0.914865
TRANSFERASE_ACTIVITY__TRANSFERRING_PHOSPHORUS_CONTAINING_GROUPS(GO:0016772)	449	0.190035	0.800154	0.582	0.916251
REGULATION_OF_SIGNAL_TRANSDUCTION(GO:0009966)	91	0.241981	0.792228	0.686	0.923141
REGULATION_OF_CELL_COMMUNICATION(GO:0010646)	91	0.241981	0.792228	0.686	0.92064
CELLULAR_PROTEIN_METABOLIC_PROCESS(GO:0044267)	856	0.145124	0.7912	0.891616	0.920578
MULTI_ORGANISM_REPRODUCTIVE_PROCESS(GO:0044703)	15	0.315802	0.786689	0.787524	0.926427
SEXUAL_REPRODUCTION(GO:0019953)	15	0.315802	0.786689	0.787524	0.923937
PROTEIN_MODIFICATION_PROCESS(GO:0036211)	529	0.188416	0.785766	0.694	0.922311
CATABOLIC_PROCESS(GO:0009056)	109	0.193083	0.784203	0.806122	0.922102
MACROMOLECULE_MODIFICATION(GO:0043412)	553	0.181259	0.779431	0.694	0.92563
CELLULAR_PROTEIN_MODIFICATION_PROCESS(GO:0006464)	528	0.185909	0.777984	0.694	0.924551
EXOPEPTIDASE_ACTIVITY(GO:0008238)	32	0.242168	0.776812	0.70101	0.923856
GUANYL_NUCLEOTIDE_EXCHANGE_FACTOR_ACTIVITY(GO:0005085)	70	0.229702	0.774479	0.617886	0.924603
DNA_BINDING(GO:0003677)	493	0.170793	0.772948	0.794715	0.92415
REGULATION_OF_SIGNALING(GO:0023051)	92	0.231214	0.771542	0.894	0.9236
CELLULAR_NITROGEN_COMPOUND_BIOSYNTHETIC_PROCESS(GO:0044271)	906	0.135286	0.77102	0.896887	0.922118
CELLULAR_COMPONENT_DISASSEMBLY(GO:0022411)	17	0.237408	0.768783	0.678068	0.922118
TRANSCRIPTION_FACTOR_COMPLEX(GO:0005667)	17	0.231396	0.76829	0.680233	0.920299
CATALYTIC_ACTIVITY__ACTING_ON_A_PROTEIN(GO:0140096)	817	0.166325	0.766871	0.786139	0.918971
PHOSPHATE_CONTAINING_COMPOUND_METABOLIC_PROCESS(GO:0006796)	507	0.162672	0.760435	0.694	0.925685
PHOSPHORUS_METABOLIC_PROCESS(GO:0006793)	507	0.162672	0.760435	0.694	0.923287
MACROMOLECULE_BIOSYNTHETIC_PROCESS(GO:0009059)	902	0.131095	0.75689	0.895257	0.925305

NAME	SIZE	ES	NES	NOM p-value	FDR q-value
PROTEIN_CATABOLIC_PROCESS(GO:0030163)	42	0.211843	0.755523	0.78672	0.924026
TRANSFERASE_ACTIVITY__TRANSFERRING_HEXOSYL_GROUPS(GO:0016758)	68	0.208939	0.754628	0.894	0.923293
MONOVALENT_INORGANIC_CATION_TRANSPORT(GO:0015672)	48	0.159101	0.753785	0.888421	0.922148
MITOCHONDRIAL_MEMBRANE_PART(GO:0044455)	23	0.292766	0.753518	0.695297	0.919924
CELLULAR_MACROMOLECULE_BIOSYNTHETIC_PROCESS(GO:0034645)	900	0.129685	0.752783	0.898467	0.919436
GENE_EXPRESSION(GO:0010467)	937	0.129032	0.750963	0.893574	0.919885
CYSTEINE_TYPE_ENDOPEPTIDASE_INHIBITOR_ACTIVITY(GO:0004869)	16	0.296907	0.747368	0.70217	0.920209
SEQUENCE_SPECIFIC_DNA_BINDING(GO:0043565)	99	0.224547	0.746744	0.801181	0.918743
DNA_TEMPLATED_TRANSCRIPTION__INITIATION(GO:0006352)	20	0.317908	0.745004	0.78501	0.919874
OXIDOREDUCTASE_ACTIVITY(GO:0016491)	351	0.159454	0.741768	0.892276	0.922309
CELLULAR_PROTEIN_CATABOLIC_PROCESS(GO:0044257)	41	0.209713	0.735295	0.792757	0.929503
PROTEOLYSIS_INVOLVED_IN_CELLULAR_PROTEIN_CATABOLIC_PROCESS(GO:0051603)	41	0.209713	0.735295	0.792757	0.927174
CELLULAR_CATABOLIC_PROCESS(GO:0044248)	97	0.178966	0.734942	0.794769	0.925361
PHOSPHORYLATION(GO:0016310)	358	0.180653	0.730998	0.694	0.927003
UDP_GLYCOSYLTRANSFERASE_ACTIVITY(GO:0008194)	19	0.322306	0.725458	0.674134	0.929626
INORGANIC_ION_TRANSMEMBRANE_TRANSPORT(GO:0098660)	35	0.205591	0.717981	0.786139	0.934116
COPPER_ION_BINDING(GO:0005507)	15	0.265591	0.714749	0.792636	0.935866
METALLOPEPTIDASE_ACTIVITY(GO:0008237)	94	0.210882	0.713848	0.70297	0.934631
GUANYL_NUCLEOTIDE_BINDING(GO:0019001)	185	0.150531	0.708871	0.801556	0.936683
GTPASE_REGULATOR_ACTIVITY(GO:0030695)	33	0.218482	0.707541	0.577002	0.936763
NUCLEOSIDE_TRIPHOSPHATASE_REGULATOR_ACTIVITY(GO:0060589)	36	0.207684	0.698386	0.567677	0.943442
CELLULAR_RESPONSE_TO_STRESS(GO:0033554)	54	0.169352	0.691578	0.795276	0.944289
CARBOXYPEPTIDASE_ACTIVITY(GO:0004180)	18	0.229853	0.690451	0.789474	0.943619
CYTOPLASM(GO:0005737)	486	0.142084	0.687416	0.89336	0.943744
REGULATION_OF_ORGANELLE_ORGANIZATION(GO:0033043)	22	0.231628	0.675991	0.696154	0.952831
PROTEIN_COMPLEX_OLIGOMERIZATION(GO:0051259)	39	0.20289	0.675417	0.78252	0.951449
PHOSPHOTRANSFERASE_ACTIVITY__ALCOHOL_GROUP_AS_ACCEPTOR(GO:0016773)	371	0.174502	0.672944	0.816	0.950295
POSITIVE_REGULATION_OF_BIOLOGICAL_PROCESS(GO:0048518)	31	0.20177	0.660489	0.682879	0.958785
PROTEIN_HOMOOLIGOMERIZATION(GO:0051260)	33	0.212749	0.656809	0.78252	0.959451
KINASE_ACTIVITY(GO:0016301)	375	0.167816	0.652822	0.816	0.962961
PROTEIN_KINASE_ACTIVITY(GO:0004672)	332	0.168395	0.647929	0.816	0.963479
METALLOCARBOXYPEPTIDASE_ACTIVITY(GO:0004181)	16	0.231714	0.646793	0.89336	0.961821
METALLOEXOPEPTIDASE_ACTIVITY(GO:0008235)	16	0.231714	0.646793	0.89336	0.959531
METALLOENDOPEPTIDASE_ACTIVITY(GO:0004222)	64	0.186526	0.644729	0.811881	0.958828
INORGANIC_CATION_TRANSMEMBRANE_TRANSPORT(GO:0098662)	34	0.182207	0.637529	0.89505	0.960618
CATION_TRANSMEMBRANE_TRANSPORT(GO:0098655)	34	0.182207	0.637529	0.89505	0.958347
DNA_REPAIR(GO:0006281)	47	0.161812	0.635875	0.809619	0.959404
GTPASE_ACTIVATOR_ACTIVITY(GO:0005096)	23	0.267633	0.631866	0.787321	0.960583
PLASMA_MEMBRANE_PART(GO:0044459)	34	0.177634	0.626703	0.794715	0.963196
PROTEIN_CONTAINING_COMPLEX(GO:0032991)	624	0.122407	0.618491	0.89336	0.965366
REGULATION_OF_CYTOSKELETON_ORGANIZATION(GO:0051493)	20	0.231026	0.618131	0.696154	0.963235
CELLULAR_RESPONSE_TO_DNA_DAMAGE_STIMULUS(GO:0006974)	53	0.14983	0.612353	0.795276	0.962885
CELL_PERIPHERY(GO:0071944)	47	0.163654	0.609352	0.895257	0.962001

NAME	SIZE	ES	NES	NOM p-value	FDR q-value
ORGANONITROGEN_COMPOUND_BIOSYNTHETIC_PROCESS(GO:1901566)	391	0.129936	0.590416	0.89336	0.972852
TRANSFERASE_COMPLEX(GO:1990234)	29	0.133583	0.579311	0.896686	0.976017
POSITIVE_REGULATION_OF_CELLULAR_PROCESS(GO:0048522)	26	0.176509	0.576552	0.892929	0.974974
NCRNA_PROCESSING(GO:0034470)	31	0.12904	0.573131	0.897087	0.975183
REGULATION_OF_CELLULAR_COMPONENT_ORGANIZATION(GO:0051128)	25	0.181971	0.562171	0.8	0.977136
CYTOPLASMIC_PART(GO:0044444)	411	0.123773	0.558376	0.895669	0.977269
HYDROLASE_ACTIVITY__ACTING_ON_CARBON_NITROGEN_(BUT_NOT_PEPTIDE)_BONDS(GO:0016810)	25	0.161804	0.513036	0.89336	0.985948
