# Supplementary material for: Possible Role of Gut Microbiota in Polypropylene Microplastics-Induced Immunity and Reproductive Dysfunction in Mice
Source: Toxics. 2026 Jul 31;14(8):679. doi: 10.3390/toxics14080679 (PMC13517389; doi:10.3390/toxics14080679)
Supplement: Supplementary file 1 [file toxics-14-00679-s001.zip › Supplementary Information S1.pdf]

Method of Humoral Immunity Assays: KLH was dissolved in 0.05 mol/L Na_2CO_3-$NaHCO_3$ buffer at 0.5 mg/mL to prepare the coating solution. The ELISA plates were coated with 100 μL of the coating solution per well and incubated overnight at 4 °C. The plates were washed three times for 3 minutes each with PBS containing 0.05% Tween-20 (PBS-T). Each well was blocked with 100 μL of 5% skim milk overnight at 4°C to reduce non-specific binding, and washed again with PBS-T three times. The serum was diluted 20-fold with PBS-T. 150μl of diluted serum was added to each well of the ELISA plates, and set two replicates for each sample. Additionally, the positive control samples and negative control samples were included. The positive control sample was serum from KLH-immunized mice, while the negative control sample was serum from KLH-naïve mice. Plates were incubated at 37°C for 3 h, and then washed with PBS-T three times. 100 μL of secondary antibody (alkaline phosphatase-conjugated anti-mouse IgG diluted 1:2000 with PBS-T) was added to each well. Plates were incubated at 37°C for 1 h, and then washed with PBS-T three times. 150 μL of the enzyme substrate p-nitrobenzene phosphate (1mg/ml in diethanolamine substrate buffer) was added to each well, and the plates were incubated at 37 °C in the dark for 20 minutes. Finally, 50 μL of 1.5 mol/L NaOH was added to each well to terminate the reaction. The optical density (OD) at 405 nm was measured using an enzyme-linked immunosorbent assay (ELISA) microplate reader, with each sample analyzed in duplicate. The level of anti-KLH antibody was directly represented by the OD value.
