# Supplementary material for: Possible Role of Gut Microbiota in Polypropylene Microplastics-Induced Immunity and Reproductive Dysfunction in Mice
Source: Toxics. 2026 Jul 31;14(8):679. doi: 10.3390/toxics14080679 (PMC13517389; doi:10.3390/toxics14080679)
Supplement: Supplementary file 1 [file toxics-14-00679-s001.zip › Supplementary Information S8.pdf]

Supplementary Information S8. Statistical results of data processing.

Sample	RawPE	Combined	Qualified	Nochime	Base(nt)	Avglen(nt)	GC	Q20	Q30
CONF1	103064	101254	99107	74101	30851827	416.35	52.60%	98.07%	93.40%
CONF2	104539	103378	101274	58616	24301066	414.58	52.92%	98.38%	94.33%
CONF3	117789	116539	114257	65392	26847625	410.56	54.63%	98.35%	94.29%
CONF4	103192	102082	100172	65336	26760264	409.58	53.07%	98.42%	94.43%
CONF5	108863	107419	105335	64909	27203654	419.1	52.52%	98.23%	93.93%
CONM1	106354	105208	103258	71433	29369076	411.14	54.03%	98.41%	94.43%
CONM2	104226	103027	100861	71370	30044622	420.97	53.44%	98.17%	93.79%
CONM3	102542	101410	99483	57710	23722494	411.06	54.31%	98.32%	94.12%
CONM4	103484	101914	99910	57822	23851527	412.5	54.47%	98.31%	94.27%
CONM5	119735	118173	115906	76369	31413606	411.34	53.83%	98.35%	94.29%
CONM6	117990	116439	114048	67968	28138625	414	54.66%	98.29%	94.19%
CONM7	105333	103804	101482	59041	24319522	411.91	54.05%	98.24%	93.95%
PPF1	103858	102655	100718	64305	26503584	412.15	53.28%	98.41%	94.45%
PPF2	97210	95878	94320	53265	22163492	416.1	53.52%	98.49%	94.74%
PPF3	102719	101046	99296	63567	26543088	417.56	52.88%	98.44%	94.49%
PPF4	106161	104138	102152	48969	20479037	418.2	54.09%	98.20%	94.06%
PPF5	69273	68398	66939	43355	18024179	415.73	53.53%	98.20%	93.96%
PPF6	86355	85236	83567	55381	22823757	412.12	53.98%	98.36%	94.30%
PPM1	110312	109266	107274	62290	25634803	411.54	55.16%	98.53%	94.88%
PPM2	104206	102373	100282	62911	26521235	421.57	53.09%	98.17%	93.84%
PPM3	106348	104876	102757	67877	28098216	413.96	54.25%	98.41%	94.45%
PPM4	115974	114384	112087	71312	29180818	409.2	53.16%	98.40%	94.46%
PPM5	82524	81094	79420	48204	19997938	414.86	54.36%	98.22%	94.01%
PPM6	104649	103257	101398	58037	23913029	412.03	53.71%	98.36%	94.29%
PPM7	93552	92317	90231	56009	23084436	412.16	54.39%	98.21%	93.95%
PPM8	98876	97452	95277	66385	27916637	420.53	52.50%	98.24%	93.93%
